# Tailoring Electronic Properties of Precision Graphene Nanoribbons via Nanopore Engineering

**DOI:** 10.1002/anie.202524299

**Published:** 2026-02-21

**Authors:** Kun Liu, Guanzhao Wen, Gianluca Serra, Nicolás Arisnabarreta, Hongde Yu, Andrea Lucotti, Yarden Peleg Walg, Hartmut Komber, Zhen‐Lin Qiu, Qing‐Song Deng, Ran He, Wenhui Niu, Thomas Heine, Eike Brunner, Mischa Bonn, Steven De Feyter, Matteo Tommasini, Hai I. Wang, Ji Ma, Xinliang Feng

**Affiliations:** ^1^ Center For Advancing Electronics Dresden (cfaed) & Faculty of Chemistry and Food Chemistry Technische Universität Dresden Dresden Germany; ^2^ Max Planck Institute for Polymer Research Mainz Germany; ^3^ Dipartimento Di Chimica Materiali Ed Ingegneria Chimica “G. Natta” Politecnico Di Milano Milano Italy; ^4^ Division of Molecular Imaging and Photonics Department of Chemistry KU Leuven Leuven Belgium; ^5^ Leibniz‐Institut Für Polymerforschung Dresden e. V. Dresden Germany; ^6^ Max Planck Institute of Microstructure Physics Halle Germany; ^7^ State Key Laboratory for Physical Chemistry of Solid Surfaces and Department of Chemistry College of Chemistry and Chemical Engineering Xiamen University Xiamen P. R. China; ^8^ Institute For Metallic Materials Leibniz Institute of Solid State and Materials Science Dresden Germany; ^9^ Nanophotonics Debye Institute for Nanomaterials Science Utrecht University Utrecht the Netherlands; ^10^ College of Materials Science and Optoelectronic Technology and Center of Materials Science and Optoelectronics Engineering University of Chinese Academy of Sciences Beijing P. R. China

**Keywords:** bandgap engineering, graphene nanoribbons, nanopore, precision synthesis

## Abstract

The precise incorporation of nanopores into graphene nanoribbons (GNRs) offers a complementary strategy for modulating their opto‐electronic properties beyond conventional width and edge engineering. However, a systematic understanding of the relationship between the structure and electronic properties of porous GNRs (**pGNRs**) remains experimentally unexplored due to the lack of rational synthetic strategies. Herein, we report two novel porous GNRs (**pGNR 1** and **pGNR 2**) synthesized via solution‐phase methods, featuring periodically arranged [18]annulene nanopores and gulf‐edged architectures, along with a nonporous GNR (**npGNR**) as a counterpart. Utilizing efficient Diels‐Alder polymerization and Scholl‐type cyclization, these GNRs attain average lengths of up to 60 nm. The chemical identities of the synthesized GNRs were comprehensively characterized by IR, Raman, and solid‐state NMR spectroscopy, complemented by theoretical calculations. To further elucidate the structural features underlying the observed properties, three representative model compounds (**1**, **2**, and **3**) corresponding to segments of the respective GNRs were synthesized and analyzed. UV–vis and THz spectroscopic analyses demonstrate that **npGNR** exhibits a relatively narrow optical bandgap of 1.63 eV and a high intrinsic charge carrier mobility of ∼40 cm^2^ V^−1^ s^−1^, whereas **pGNR 2** displays a wider bandgap of 1.91 eV with a reduced mobility of ∼27 cm^2^ V^−1^ s^−1^. This study systematically elucidates the effects of nanopore incorporation on the electronic structure and charge transport properties of GNRs, offering a rational design framework for the design of nanopore‐engineered carbon‐based electronic materials.

## Graphical abstract

Novel porous graphene nanoribbons incorporating [18]annulene nanopores, together with a nonporous GNR of identical width, have been synthesized. Spectroscopic and theoretical analyses reveal that nanopore incorporation effectively tunes their electronic properties by enlarging the bandgap, reducing charge‐carrier mobility, and modulating exciton dynamics, thereby establishing a versatile strategy for the design of porous graphene nanostructures with tailored optoelectronic characteristics.

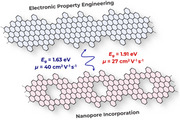

## 1 Introduction

Precision graphene nanoribbons (GNRs) have attracted considerable interest as promising materials for nanoscale electronic devices due to their highly tunable electronic properties [1, 2, 3, 4, 5]. Utilizing bottom‐up synthesis, via either surface‐assisted or solution‐based precision chemistry, the opto‐electronic and physicochemical properties of GNRs can be meticulously customized by modulating their width and edge structures [6, 7, 8, 9, 10, 11, 12]. Moreover, the deliberate introduction of well‐defined nanopores into graphene nanostructures not only alters their topological geometry but also enables modulation of their physical characteristics, broadening their potential applications in optical modulation [13, 14, 15, 16], thermoelectric devices [17, 18], and ion permeation [19]. Building on this concept, the incorporation of nanopores into GNRs brings a new dimension for engineering their opto‐electronic properties, with the effects primarily dictated by the shape, size, and distribution of the nanopores [20, 21, 22]. To date, experimental access to porous GNRs (**pGNRs**) remains limited, with most reported cases derived from on‐surface synthesis under ultrahigh‐vacuum conditions [23, 24, 25, 26, 27, 28]. However, this approach is hindered by low yield, undesired formation of cyclic oligomers, and restricted coherent π‐conjugation due to the presence of C–C single bonds along the GNR edges [29]. Recently, our group reported the first example of solution‐synthesized porous GNR via A_2_B_2_ Suzuki polymerization between a bis‐boronic ester macrocyclic monomer and a dihalogenated monomer, followed by the Scholl cyclization [30]. However, the longitudinal extension of these GNRs was limited by the low efficiency of the Suzuki polymerization step, primarily due to the high steric hindrance between the coupling monomers and the heterogeneous biphasic conditions. Furthermore, the structure–property relationship in GNRs associated with the nanopores remains largely unexplored.

In this work, we demonstrate two novel porous GNRs (**pGNR 1** and **pGNR 2**), accompanied by a nonporous extended gulf‐edged GNR (**npGNR**) featuring the same width via A_2_B_2_ Diels‐Alder polymerization of a diethynyl monomer and cyclopentadienone derivative, followed by cyclodehydrogenation reaction. As representative segments of **pGNR 1**, **pGNR 2**, and **npGNR**, three model compounds (**1**, **2**, and **3**) were synthesized in excellent yields to manifest the high efficiency of the Scholl reaction. Crystallographic analysis of compound **1** reveals the characteristic porous topology, featuring a [18]annulene inner cavity with a diameter of ∼6 Å. The successful formation of the targeted GNRs was verified through a combination of infrared (IR), Raman, and solid‐state NMR spectroscopies. **pGNR 2** exhibits a broader optical bandgap of 1.91 eV, representing an increase of 0.28 eV compared to the **npGNR** of the same width, consistent with the calculated trend in bandgap values. Furthermore, time‐resolved terahertz (THz) spectroscopy reveals that intrinsic charge carrier mobility decreases from ∼40 cm^2^ V^−^^1^ s^−^^1^ for **npGNR** to ∼27 cm^2^ V^−^^1^ s^−^^1^ for **pGNR 2**. Further analysis of frequency‐resolved photoconductivity unveils that the incorporation of nanopores does not increase the charge scattering effects in GNRs. Instead, the reduced photoconductivity observed in **pGNR 2** stems from an increased effective mass arising from its porous structure. This study experimentally demonstrates the impact of periodic nanopores on the charge transport properties of GNRs within a well‐defined model system, offering valuable insights for the rational design and implementation of GNR‐based nanoelectronics.

## 2 Results and Discussion

**SCHEME 1:**
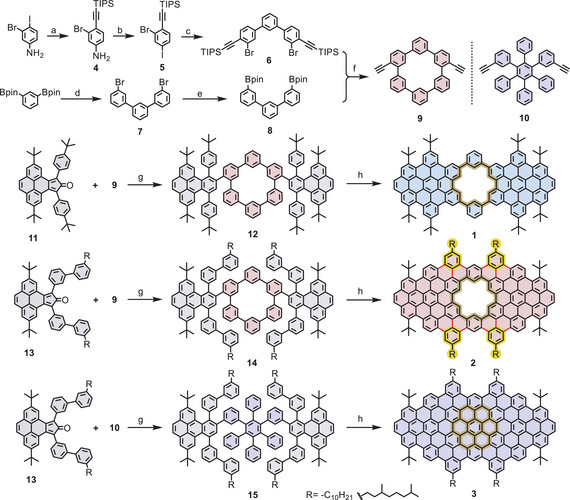
Synthetic route toward model compounds **1**, **2**, and **3**. Reagents and conditions: (a) (triisopropylsilyl)acetylene, CuI, PdCl_2_(PPh_3_)_2_, TEA, THF, r.t., 24 h, 95%; (b) NaNO_2_, KI, HCl/H_2_O/MeCN, 50–80°C, 30 min, 57%; (c) 1,3‐bis(4,4,5,5‐tetramethyl‐1,3,2‐dioxaborolan‐2‐yl)benzene, Pd(PPh_3_)_4_, K_2_CO_3_, THF/EtOH/H_2_O, 60°C, 48 h, 73%; (d) 1‐bromo‐3‐iodobenzene, Pd(PPh_3_)_4_, K_2_CO_3_, toluene/EtOH/H_2_O, 60°C, 24 h, 90%; (e) B_2_pin_2_, Pd(dppf)Cl_2_, KOAc, 1,4‐dioxane, 100°C, 12 h, 95%; (f) i. Pd_2_(dba)_3_, [(*tert*‐Bu)_3_PH]BF_4_, NaHCO_3_, THF/H_2_O, 80 °C, 3 days, 12%; ii. TBAF, THF, r.t., 20 min, 86%; (g) Ph_2_O, 260°C, 24 h, 92% for compound **12**, 90% for compound **14** and 87% for compound **15**; (h) FeCl_3_, CH_3_NO_2_, CH_2_Cl_2_, r.t., 1 h, 90% for compound **1**, and 2 h, 94% for compound **2**, and 2 h, 92% for compound **3**.

As illustrated in Scheme 1, model compounds **1**, **2**, and **3**, representing well‐defined segments of **pGNR 1**, **pGNR 2**, and **npGNR**, respectively, were synthesized. These model systems serve as molecular references for understanding the electronic structure and provide strong validation of the efficiency of GNR formation. First, 3‐bromo‐4‐((triisopropylsilyl)ethynyl)aniline (**4**) was prepared in 95% yield by the selective Sonogashira coupling of 3‐bromo‐4‐iodobenzenamine with (triisopropylsilyl)acetylene. Subsequently, compound **4** was converted into ((2‐bromo‐4‐iodophenyl)ethynyl)triisopropylsilane (**5**) by Sandmeyer reaction in 57% yield. Afterwards, compound **6** was prepared via a twofold Suzuki coupling of 1,3‐bis(4,4,5,5‐tetramethyl‐1,3,2‐dioxaborolan‐2‐yl)benzene with compound **5** in 73% yield. Meanwhile, 3,3′′‐dibromo‐1,1′:3′,1′′‐terphenyl (**7**) was synthesized through a Suzuki coupling between 1,3‐bis(4,4,5,5‐tetramethyl‐1,3,2‐dioxaborolan‐2‐yl)benzene and 1‐bromo‐3‐iodobenzene in 90% yield. Subsequent Miyaura borylation gave 3,3''‐bis(4,4,5,5‐tetramethyl‐1,3,2‐dioxaborolan‐2‐yl)‐1,1':3',1''‐terphenyl (**8**) in 95% yield. Afterward, the Suzuki coupling of **6** and **8** at a dilute concentration, followed by treatment with tetrabutylammonium fluoride (TBAF), provided diethynyl‐[6]cyclo‐meta‐phenylene macrocycle **9** with a yield of 10 % over two steps. For the synthesis of a nonporous model compound as a counterpart, diethynyl‐hexaphenylbenzene **10** was prepared following a previously reported procedure with slight modifications [9]. With macrocyclic building block **9** in hand, a subsequent Diels–Alder reaction with cyclopentadienone **11** afforded the corresponding oligophenylene precursor **12** in 92% yield. Finally, the Scholl reaction of **12**, carried out using iron chloride as the Lewis acid and oxidant, yielded model compound **1** in 90% yield. Following a similar synthetic strategy to that of compound **1**, the π‐expanded porous model compound **2** and the corresponding nonporous model compound **3** with the same width, were obtained in 94% and 92% yields, respectively from precursors **14** and **15** under the same Scholl reaction condition.

**FIGURE 1:**
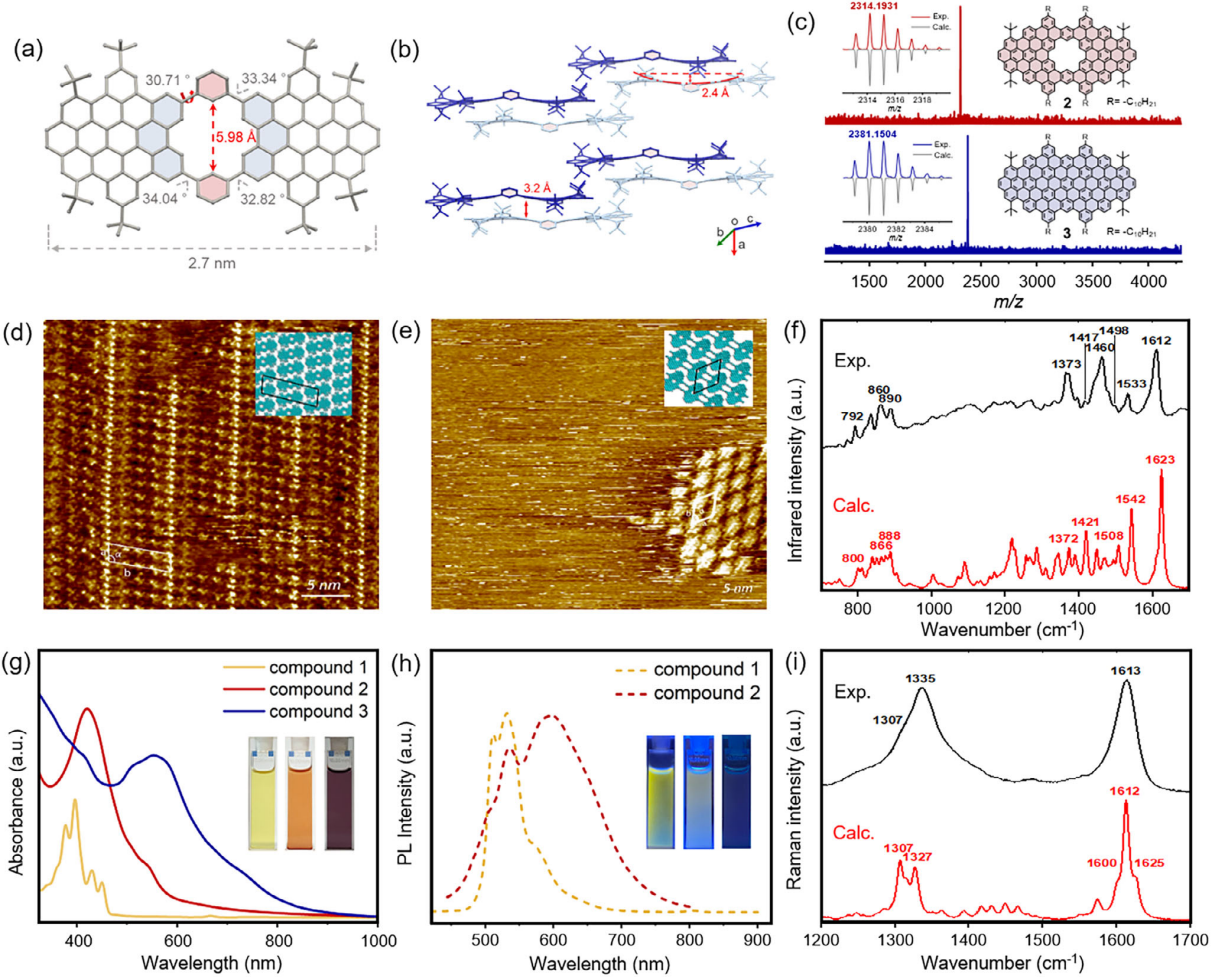
(a, b) X‐ray crystallographic structure of **1** from the top and side views. Hydrogen atoms and solvent molecules are omitted for clarity. (c) HR MALDI‐TOF MS spectra of **2** and **3**. (d) STM image of **1** self‐assembled at the HOPG/TCB interface; the inset shows a tentative model based on the experimentally extracted unit‐cell parameters: a=1.6 ± 0.2 nm, b=6.2 ± 0.4 nm, and α=109 ± 2°. *U*_bias_=‐0.5 V, *I*_set_=0.18 nA. (e) STM image of **2** self‐assembled at the HOPG/TCB interface; the inset shows a tentative model based on the experimentally extracted unit‐cell parameters: *a*=2.5 ± 0.1 nm, *b*=2.7 ± 0.1 nm, and *α*=63 ± 2°. *U*_bias_=‐0.75 V, *I*_set_=0.12 nA. (f) Normalized experimental and calculated IR spectra of **2**. (g) UV–vis absorption spectra of **1**, **2**, and **3** in THF (10^−5^ M); the inset shows the photographic images of THF solutions of **1**, **2**, and **3** under ambient light. (h) PL spectra of **1** and **2** in THF (10^−5^ M); the inset shows the photographic images of THF solutions of **1**, **2**, and **3** under ultraviolet light at 365 nm. (i) Normalized experimental and calculated Raman spectra of **2**.

The successful formation of **1**‐**3** was first confirmed by MALDI‐TOF MS analysis, in which the observed spectra were in good agreement with the simulated isotopic distribution patterns (Figure 1c and Figure S1). Thanks to its good solubility, the chemical structure of **1** was validated by ^1^H NMR spectroscopy and corresponding 2D NMR measurements (Figure S57). Moreover, a single crystal of **1** was obtained by slow vapor diffusion of methanol into a solution of compound **1** in 1,2‐dichlorobenzene, allowing for single‐crystal X‐ray diffraction measurement. Compound **1** crystallized along with 1,2‐dichlorobenzene molecules in a triclinic space group *P*1̅. As depicted in Figure 1a, 1 features a characteristic porous structure with an [18]annulene nanopore with a diameter of 5.98 Å. Additionally, the structure exhibited a bowl‐shaped geometry with a depth of 2.4 Å, attributed to steric repulsion between the phenanthrene units and the adjacent, tilted phenyl rings, which exhibited dihedral angles ranging from 30.71° to 34.04°. Furthermore, compound **1** forms racemic dimers via face‐to‐face *π*–*π* interactions with an interlayer distance of 3.2 Å (Figure 1b). These dimers further exhibit a brick‐layer stacking along the *a*‐axis by aligning with another pair of racemic dimers via *π*–*π* interactions. The structural studies of model compounds **1** and **2** were further carried out via scanning tunnelling microscopy (STM) at the highly oriented pyrolytic graphite (HOPG)/1,2,4‐trichlorobenzene (TCB) interface (Figure 1d and e). Accordingly, Figures 1d and S22 present STM images of an ordered network of bright units arranged in rows of alternating contrast for **1**, presumably bearing the tert‐butyl groups in the upright position. Similar STM experiments were performed for **2,** as observed in the STM images presented in Figures 1e and S23. These images exhibit an oblique arrangement of high‐contrast units. It is suggested that the peripheral decyl alkyl chains (represented as propyl) are pointing upwards into the supernatant solution and without interdigitation [31, 32]. Furthermore, the poor stability of **2** at the surface may result from the lack of strong intermolecular interactions, as only small domains are visualized. In contrast to **1** and **2**, compound **3** exhibited low affinity for the HOPG surface and a strong tendency to aggregate, which consistently resulted in only large, high‐contrast blobs in STM measurements, despite our efforts under various conditions. Single‐crystal analysis and solution NMR characterizations of **2** and **3** were also not possible due to their poor solubility, which is attributed to the planar skeleton and pronounced aggregation in common organic solvents. Nevertheless, to validate the chemical structure of model compounds **2** and **3**, we conducted IR and Raman measurements, and compared the obtained results with DFT‐calculated spectra (Figure S9). Concerning the IR spectrum of **2** (Figure 1f), we assign the two features measured at 792 and 890 cm^−1^ to the DUO and SOLO vibrations [33], respectively, which are computed at 800 and 888 cm^−1^. The relatively intense bands at 860 and 1373 cm^−1^ are vibrational markers of the nanopore, computed at 866 and 1372 cm^−1^ and assigned to out‐of‐plane and in‐plane CH bending in the pore, respectively (Table S5). The intense band observed at 1460 cm^−1^ does not have a clear computational counterpart. We tentatively assign this feature to the CH_2_ scissoring mode of the C_10_H_21_ alkyl chains, which are shorter in our model, and therefore provide a weaker transition. On the other hand, the Raman spectrum (Figure 1i) of **2** shows the two characteristic D‐ and G‐bands as expected for molecular graphene, at 1335 and 1613 cm^−1^, which are assigned to ring‐breathing and CC stretching modes, respectively.

To evaluate the influence of nanopore defects on optical properties, model compounds **1**‐**3** were further investigated using UV–vis absorption and fluorescence spectroscopy in anhydrous tetrahydrofuran. The UV‐vis spectrum of the nonporous compound **3** exhibited a main absorption at 556 nm, corresponding to its intense violet color (Figure 1g). In contrast, the porous compounds **1** and **2** displayed significantly blue‐shifted main absorption peaks at 396 nm and 420 nm, respectively, aligning well with the DFT‐calculated spectra (Figure S7a). The optical energy gaps (*E*_gap_) of compounds **1**‐**3** were estimated from the onset wavelengths of their absorption spectra, revealing the narrowest gap for **3** (1.69 eV), followed by those of **2** (2.06 eV) and **1** (2.66 eV). The nonporous model compound **3** exhibited no detectable luminescence due to the aggregation‐caused quenching (ACQ) effect, which is commonly observed in polycyclic aromatic hydrocarbons (PAHs) without structural defects [14, 34]. In contrast, as its porous counterpart with the same size, compound **2** emitted orange fluorescence at 597 nm (Figure 1h). These findings underscore the crucial role of engineered nanopores in modulating the bandgap and enhancing photoluminescence (PL) in nanographenes.

**SCHEME 2:**
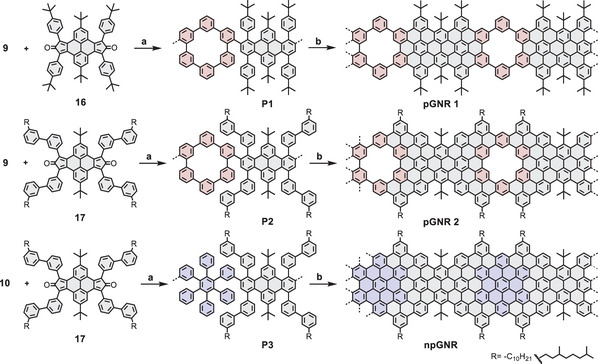
Synthesis of **pGNR 1** and **pGNR 2** as well as **npGNR**. Reagents and conditions: (a) Ph_2_O, 260°C, 24 h, 96% for **P1**, 93% for **P2**, and 95% for **P3**; (b) FeCl_3_, CH_3_NO_2_, CH_2_Cl_2_, r.t., 3 days, 95% for **pGNR 1**, 91% for **pGNR 2**, and 94% for **npGNR**.

**FIGURE 2:**
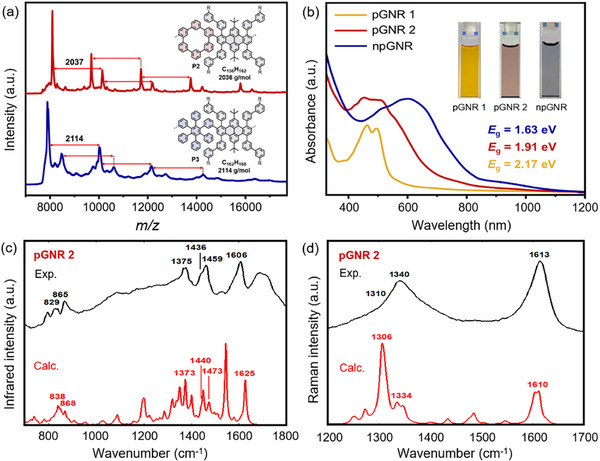
Spectroscopic characterizations of polymer precursors and GNRs. (a) MALDI‐TOF MS analysis of polymer **P2** and **P3** (matrix: DCTB, linear mode). (b) UV‐vis absorption spectra of **pGNR 1**, **pGNR 2**, and **npGNR** in NMP (0.1 mg mL^−1^). (c) Normalized experimental (black line) and calculated (red line) IR spectra of **pGNR 2**. (d) Normalized experimental (black line) and calculated (red line) Raman spectra of **pGNR 2**.

Encouraged by the successful synthesis of model compounds, the synthesis of the corresponding GNRs was further investigated. With building blocks **9** and **10** in hand, a subsequent Diels–Alder reaction with cyclopentadienones (**16** or **17**) afforded polyphenylene precursors **P1**, **P2**, and **P3** in yields of 96%, 93%, and 95%, respectively (Scheme 2). Linear‐mode MALDI‐TOF MS analysis of the crude polymers revealed a family of signals spaced by 1396, 2037, and 2114 g mol^−1^ for **P1**, **P2**, and **P3**, respectively, corresponding to the molecular weight of their repeat units (Figures 2a and S3). Subsequently, the high‐molecular‐weight polyphenylene precursors were fractionated using recycling gel permeation chromatography (GPC), followed by analytical size‐exclusion chromatography (SEC) analysis against linear polystyrene (PS) standards. The analysis of **P1** revealed a number‐average molar mass (*M*_n_) of ∼ 39.3 kDa with a narrow polydispersity (*Đ*) of ∼1.07 (Figure S2), whereas **P2** and **P3** exhibited higher *M*_n_ of ∼ 48.5 kDa and 56.4 kDa, along with broader dispersities of ∼1.23 and 1.55, respectively (Figures S4 and S5). Finally, cyclodehydrogenation of the obtained fractions of **P1**‐**P3** with the highest molecular weight via the Scholl reaction using FeCl_3_ yielded the targeted **pGNR 1**, **pGNR 2**, and **npGNR** with yields of 95%, 91%, and 94%, respectively. The average lengths of the resulting **pGNR 1**, **pGNR 2**, and **npGNR** were estimated to be approximately 60 nm, 50 nm, and 55 nm, based on their number‐average degree of polymerization ($\bar{DP}$ *_n_* ≈ 28 for **P1**, $\bar{DP}$ *_n_* ≈ 24 for **P2**, and $\bar{DP}$ *_n_* ≈ 26 for **P3**) and the length of the corresponding repeat unit. Owing to the presence of bulky *tert*‐butyl substituents and branched 3,7‐dimethyloctyl chains at the ribbon edges, the obtained GNRs exhibited good dispersibility in common organic solvents (0.1 mg mL^−1^), such as *N*‐methyl‐2‐pyrrolidone (NMP), chlorobenzene, and 1,2,4‐trichlorobenzene.

The successful conversion of **P1**‐**P3** into GNRs was initially corroborated through a comprehensive analysis involving IR and Raman spectroscopies, complemented by DFT simulations. In the experimental IR spectrum of **pGNR 2** (Figure 2c), a prominent band appears at 865 cm^−1^, which closely aligns with the DFT‐predicted peak at 868 cm^−^^1^ and is attributed to the out‐of‐plane CH bending vibration localized at the nanopore. Another vibrational marker of the nanopore is a shoulder at 1436 cm^−1^ (computed at 1440 cm^−1^), corresponding to in‐plane CH bending at the pore. Additionally, a broad band observed at around 1695 cm^−1^ can be assigned to the CO stretching of a carbonyl group attached to a sp^2^ carbon at the GNR terminus, as well as infrared combination bands observed in aromatic systems [35, 36]. Furthermore, the unexpectedly weak intensity of the band predicted at 1542 cm^−1^, which corresponds to in‐plane CH bending within the pore, can be attributed to solid‐state effects that are not considered in our gas‐phase model. The Raman spectrum of **pGNR 2** (Figure 2d), supported by DFT calculations, reveals the characteristic D‐ and G‐bands of molecular graphene, corresponding to collective ring‐breathing and C–C stretching vibrations, respectively. Moreover, solid‐state magic‐angle spinning (MAS) NMR measurements further support the successful graphitization of polymer **P2** and **P3** into the corresponding **pGNR 2** and **npGNR**. The ^1^H MAS NMR of both **pGNR 2** and **npGNR** exhibits slightly decreased chemical shifts, suggesting the formation of planarized structures with less agglomeration and reduced ring‐current shifts (Figure S16). In addition, the ^13^C cross‐polarization (CP) and directly excited (DE) MAS NMR spectra further corroborate the suggested structures (Figure S17).

**FIGURE 3:**
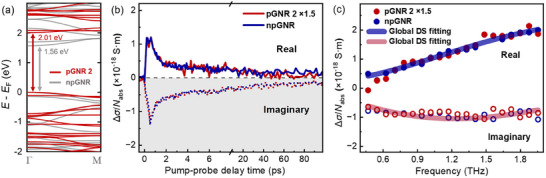
Band structures and charge transport properties of **pGNR 2** and **npGNR**. (a) Calculated band structures of **pGNR 2** (red) and **npGNR** (gray). (b) Time‐resolved and (c) frequency‐resolved complex terahertz photoconductivity normalized to the absorbed photon density (Δ*σ*/*N*_abs_) of both **pGNR 2** and **npGNR** after photoexcitation at 3.1 eV. The solid lines in (c) correspond to a global fitting by the Drude–Smith model to **pGNR 2** and **npGNR**.

Furthermore, the UV‐vis absorption spectra of the three GNRs dispersed in NMP are presented in Figure 2b. For **pGNR 1**, a distinct absorption peak with the longest wavelength is observed at 495 nm, whereas **pGNR 2** exhibits a significantly red‐shifted absorption peak at 510 nm, attributed to π‐extension via the fusion of four additional phenyl rings along the gulf edges. In contrast to **pGNR 2**, the nonporous counterpart, **npGNR**, displays a pronounced bathochromic shift, with an absorption maximum at 601 nm, originating from extended electronic conjugation along the GNR backbone. Based on the Tauc plot method (Figure S6), **pGNR 1** exhibits a broad optical bandgap of 2.17 eV, whereas **pGNR 2** shows a narrower bandgap of 1.91 eV, being 0.28 eV larger than that of **npGNR** (1.63 eV). To elucidate the electronic effect of nanopore incorporation in the GNR, the band structure of **pGNR 2** and its nonporous counterpart (**npGNR**) were calculated using the projector augmented‐wave (PAW) method in conjunction with the Perdew–Burke–Ernzerhof (PBE) exchange‐correlation functional (Figure 3a). These results reveal that the introduction of nanopores into the GNR backbone significantly alters the electronic structure, impacting both the bandgap and band dispersion. Specifically, **pGNR 2** exhibits a near‐flat dispersion near the valence band maximum (VBM) and conduction band minimum (CBM), with bandwidths of ∼0.18 eV (VB) and ∼0.15 eV (CB), respectively, resulting in a wider bandgap of 2.01 eV. In contrast, **npGNR** exhibits more pronounced band dispersion, with wider VB and CB bandwidths of ∼0.26 eV and ∼0.29 eV, respectively, and a narrower bandgap of 1.56 eV, corresponding to a smaller effective mass (m^*^) and more delocalized charge carriers.

To further assess the influence of periodic nanopores on the carrier transport properties in GNRs, we performed the optical pump‐terahertz probe (OPTP) spectroscopy [37, 38] on **pGNR 1**, **pGNR 2**, and **npGNR** films following excitations at 3.1 eV. For **pGNR 1**, light excitation results in zero real conductivity as shown in Figure S24, indicating complete exciton formation within the sample [39]. We discuss the plausible reasons for this purely excitonic response in **pGNR 1** in the supporting information. Figure 3b shows the time‐resolved photoconductivity normalized to the absorbed photon density (Δ*σ*/*N*_abs_) of **pGNR 2** and **npGNR**. Both exhibit a sub‐picosecond rise in photoconductivity, consistent with the rapid generation of free carriers after photoexcitation. The subsequent decay, with a weighted average lifetime of ∼ 3 ps (fitting by exponential functions), corresponds to the carrier trapping and/or exciton formation [39, 40]. Remarkably, when the photoconductivity signals of **pGNR 2** are scaled by a factor of 1.5, the overall dynamics closely match those of **npGNR**, indicating comparable carrier lifetimes but a reduced intrinsic photoconductivity in **pGNR 2** after introducing the pore structure into GNRs.

To investigate the origin of the lower photoconductivity of **pGNR 2**, we recorded the frequency‐resolved photoconductivity at ∼0.8 ps after excitation. As shown in Figure 3c, after rescaling the photoconductivity of **pGNR 2** by a factor of 1.5, the photoconductivity of both GNRs exhibits an indistinguishable frequency dependence. This result suggests that both GNRs share a similar scattering mechanism. To quantitatively describe the transport properties, the frequency‐resolved photoconductivity spectra are globally fitted using the phenomenological Drude‐Smith model [39, 41]. This model provides scattering times (*τ*_DS_) as 52 ± 2 fs and backscattering parameters (*c*) as ‐0.95 ± 0.01. These results indicate that introducing nanopores does not necessarily increase the charge scattering. Rather, the relatively low photoconductivity observed in **pGNR 2** can be attributed solely to an increased effective mass (*m* ^*^) caused by its porous architecture, as supported by DFT calculations (Table S15): specifically, **npGNR** exhibits a significantly lower m^*^ value (0.115 *m*_0_) compared to **pGNR 2** (0.169 *m*_0_). This effect may be attributed to the fact that the periodic nanopores interrupt the conjugation pathway, reducing the curvature of the electronic bands and thus increasing the carrier inertia. Based on the obtained microscopic parameters for transport, we can further estimate the charge carrier mobility in **npGNR** and **pGNR 2**. The intrinsic mobility (*µ*), calculated as *µ* = *eτ*_DS_/*m* ^*^, is high for both **npGNR** and **pGNR 2** (795 ± 30 vs. 541 ± 21 cm^2^ V^−^^1^ s^−^^1^, respectively). Similarly, the *dc*‐limit mobility (*µ*_dc_), estimated via *µ*_dc_ = *µ*(1 + c), is 40 ± 8 cm^2^ V^−1^s^−^^1^ for **npGNR** and 27 ± 5 cm^2^ V^−^^1^ s^−^^1^ for **pGNR 2**. Our results highlight the critical role of backbone continuity in controlling exciton dynamics and achieving high charge mobility in GNRs.

## 3 Conclusion

In summary, we established a model system to experimentally investigate the influence of nanopores on the opto‐electronic properties of precision GNRs. Through an efficient Diels–Alder polymerization strategy and Scholl‐type cyclization, a novel class of porous GNRs, accompanied by a nonporous GNR were synthesized with average lengths of up to 60 nm. To elucidate the structural features of the corresponding GNRs, three representative model compounds (**1**, **2**, and **3**) were synthesized in high yields. Single‐crystal X‐ray analysis of compound **1** confirmed a well‐defined porous structure, characterized by a [18]annulene cavity with a diameter of 5.98 Å. The obtained GNRs were thoroughly characterized by IR, Raman, and solid‐state NMR spectroscopies. The incorporation of nanopores induced marked alterations in the opto‐electronic properties. Both model compound **2** and **pGNR 2** exhibited pronounced blue shifts in their absorption spectra and improved solubility compared to the nonporous counterpart. Optical measurements indicated that incorporating nanopores serves as an effective strategy for modulating the electronic bandgap of GNRs, as evidenced by **pGNR 2**, which exhibited an optical bandgap of 1.91 eV—0.28 eV wider than that of its nonporous counterpart (**npGNR**, 1.63 eV). THz spectroscopy further revealed that introducing nanopores does not increase charge scattering but instead flattens the band dispersion, leading to an increased effective mass and a consequent reduction of charge carrier mobility from nearly 40 cm^2^ V^−^^1^ s^−^^1^ for **npGNR** to around 27 cm^2^ V^−^^1^ s^−^^1^ for **pGNR 2**. This study presents an effective strategy for engineering the electronic properties of GNRs through nanopore incorporation and provides valuable insights for the rational design of porous graphene nanostructures for applications in optoelectronic devices, with potential extensions to thermal management, ion transport, and DNA sequencing.

## Conflicts of Interest

The authors declare no conflicts of interest.

## Supporting information




**Supporting File 1**: anie71508‐sup‐0001‐SuppMat.pdf.

## Data Availability

The data that support the findings of this study are available from the corresponding author upon reasonable request.
