# Supplementary material for: Tailoring Electronic Properties of Precision Graphene Nanoribbons via Nanopore Engineering
Source: Angew Chem Int Ed Engl. 2026 Feb 21;65(14):e24299. doi: 10.1002/anie.202524299 (PMC13023704; doi:10.1002/anie.202524299)
Supplement: Supplementary file 1 — Supporting File 1: anie71508‐sup‐0001‐SuppMat.pdf. [file ANIE-65-e24299-s001.pdf]

Supporting Information

Tailoring Electronic Properties of Precision Graphene Nanoribbons via Nanopore Engineering

Kun Liu[1], Guanzhao Wen[2], Gianluca Serra[3], Nicolás Arisnabarreta[4], Hongde Yu[1], Andrea Lucotti[3], Yarden Peleg Walg[1], Hartmut Komber[5], Zhen-Lin Qiu[1,10], Qing-Song Deng[6], Ran He[7], Wenhui Niu[1,10], Thomas Heine[1], Eike Brunner[1], Mischa Bonn[2], Steven De Feyter[4], Matteo Tommasini[3], Hai I. Wang[2,8], Ji Ma[9*], Xinliang Feng[1,10*]

[1] Center for Advancing Electronics Dresden (cfaed) & Faculty of Chemistry and Food Chemistry, Technische Universität Dresden, Mommsenstrasse 4, 01062 Dresden, Germany

[2] Department of Molecular Spectroscopy, Max Planck Institute for Polymer Research, Ackermannweg 10, 55128 Mainz, Germany

[3] Dipartimento di Chimica, Materiali ed Ingegneria Chimica "G. Natta", Politecnico di Milano, Piazza Leonardo da Vinci 32, 20133 Milano, Italy

[4] Division of Molecular Imaging and Photonics, Department of Chemistry, KU Leuven, B-3001 Leuven, Belgium

[5] Leibniz-Institut für Polymerforschung Dresden e. V., Hohe Straße 6, 01069 Dresden, Germany

[6] State Key Laboratory for Physical Chemistry of Solid Surfaces and Department of Chemistry, College of Chemistry and Chemical Engineering, Xiamen University, 361005 Xiamen, P. R. China

[7] Institute for Metallic Materials, Leibniz Institute of Solid State and Materials Science, 01069 Dresden, Germany

[8] Nanophotonics, Debye Institute for Nanomaterials Science, Utrecht University, Princetonplein 1, 3584 CC Utrecht, The Netherlands

[9] College of Materials Science and Optoelectronic Technology and Center of Materials Science and Optoelectronics Engineering, University of Chinese Academy of Sciences, 100049 Beijing, P. R. China

[10] Max Planck Institute of Microstructure Physics, Weinberg 2, 06120 Halle, Germany

Contents

1. General methods and materials S3

2. Synthetic procedures and characterization S4

3. Crystallographic data of **1** S18

4. Optical properties of model compounds and GNRs S19

5. IR and Raman characterization S20

6. Solid-state NMR analysis S30

7. Theoretical calculations S33

8. STM experiments at the solid/liquid interface S35

9. Terahertz spectroscopic study of **pGNR 1** S36

10. NMR spectra S37

11. References S56

1. General methods and materials

General remarks: All the reagents were obtained from TCI, BLDpharm, Sigma Aldrich, abcr, Acros organics or Strem. All these chemicals were used as received without further purification. Anhydrous toluene, tetrahydrofuran (THF), dimethylformamide (DMF) and dichloromethane (DCM) were obtained from MBRAUN MB-SPS-5 solvent purification system. All the sensitive reactions were performed using standard vacuum-line and Schlenk techniques. Thin layer chromatography (TLC) was performed on silica-coated aluminum sheets with a fluorescence indicator (TLC silica gel 60 F_{254}, purchased from Merck KGaA). Column chromatography was performed on silica (SiO_2, particle size 0.063–0.200 mm, purchased from VWR).

NMR spectra were recorded on a Bruker AV-II 300 MHz spectrometer operating at 300.13 MHz for ^{1}H and at 75.47 MHz for ^{13}C, on a Bruker Avance III 500 MHz spectrometer operating at 500.13 MHz for ^{1}H and at 125.77 MHz for ^{13}C or on a Bruker Avance III 600 MHz spectrometer operating at 600.16 MHz for ^{1}H and 150.92 MHz for ^{13}C. Chemical shifts (δ) were reported in ppm. Coupling constants (J values) were presented in Hertz (Hz). ^{1}H NMR chemical shifts were referenced to CD_2Cl_2 (5.32 ppm). ^{13}C NMR chemical shifts were referenced to CD_2Cl_2 (53.7 ppm). The following abbreviations are used to describe peak patterns as appropriate: s = singlet, d = doublet, t = triplet, q = quartet, and m = multiplet.

High resolution mass spectrometry (HRMS) was performed on a Bruker Autoflex Speed MALDI TOF MS (Bruker Daltonics, Bremen, Germany) using DCTB (*trans*-2-[3-(4-*tert*-butylphenyl)-2-methyl-2-propenylidene]malononitrile) as matrix. HR-ESI mass spectra were recorded on a Waters Xevo G2-XS QTOF mass spectrometer.

Analytical size-exclusion chromatography (SEC) was performed on gel permeation chromatography (GPC) with an Aligent Technologies 1260 Infinity II LC system equipped with two Resipore columns and RI and UV-vis detection using chloroform as eluent with a flow rate of 1 mL min^{-1} at a temperature of 40 °C. The molar masses were calculated relative to polystyrene standards with low dispersity.

UV-visible spectra were measured on an Agilent Cary 5000 UV-vis-NIR spectrophotometer by using 10 mm optical-path quartz cell at room temperature. Fluorescence spectra were recorded at room temperature on a PerkinElmer Fluorescence Spectrometer LS 55 using a 10 mm fluorescence quartz cell.

2. Synthetic procedures and characterization

Compound **8**[1], **11**[2], **16**[2], **18**[3], **19**[4], **20**[5], **24**[6], **25**[6] and were synthesized according to previous reported procedure with slight modification.

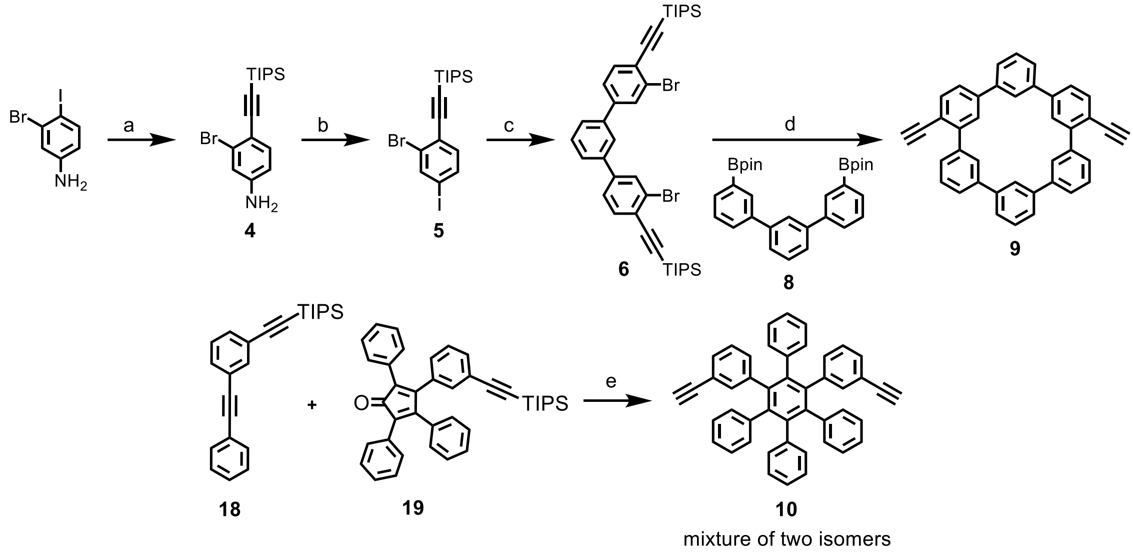

Scheme 1. Synthetic route toward building blocks **9** and **10**. Reagents and conditions: (a) (triisopropylsilyl)acetylene, CuI, $PdCl_2(PPh_3)_2$, TEA, THF, r.t., 24 h, 95%; (b) $NaNO_2$, KI, $HCl/H_2O/MeCN$, 50 °C–80 °C, 30 min, 57%; (c) 1,3-bis(4,4,5,5-tetramethyl-1,3,2-dioxaborolan-2-yl)benzene, $Pd(PPh_3)_4$, K_2CO_3, $THF/EtOH/H_2O$, 60 °C, 48 h, 73%; (d) i. $Pd_2(dba)_3$, [(*tert*-Bu)$_3$PH]BF_4, $NaHCO_3$, THF/H_2O, 80 °C, 3 days, 12%; ii. TBAF, THF, r.t., 20 min, 86%; (e) i. Ph_2O, 260 °C, 24 h, 56%; ii. TBAF, THF, r.t., 20 min, 89%.

3-Bromo-4-((triisopropylsilyl)ethynyl)aniline (4)

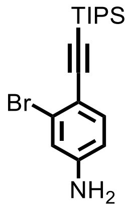

To a degassed solution of 3-bromo-4-iodoaniline (5 g, 16.78 mmol) in 30 mL of anhydrous THF were added $PdCl_2(PPh_3)_2$ (235.6 mg, 335.66 µmol), CuI (127.85 mg, 671.32 µmol) and triethylamine (2.55 g, 25.17 mmol) under argon. After stirring for 5 minutes, (triisopropylsilyl)acetylene (4.59 g, 25.17 mmol) was added dropwise to the reaction mixture (slightly exothermic). After full consumption of the starting material (monitored by TLC), the reaction mixture was diluted with DCM, washed with a saturated aqueous solution of ammonium chloride, brine and dried over $MgSO_4$. The solvent was removed under reduced pressure and the residue was purified by column chromatography on silica gel (eluent: *iso*-hexane:EA = 10:1) to give compound **4** (5.61 g, 95%). HRMS (ESI, m/z): calcd for $[M+H]^+$, 352.1096; observed 352.1087, error = -2.56 ppm. ^{1}H NMR (300 MHz, CD_2Cl_2): 7.31 (d, 8.3 Hz, 1H), 6.87 (d, 2.3 Hz, 1H), 6.54 (dd, 8.3 Hz, 2.3 Hz, 1H), 3.96 (s, 2H), 1.19 (s, 21H). ^{13}C NMR (75 MHz, CD_2Cl_2): 148.4, 135.0, 126.9, 118.3, 114.9, 113.9, 106.1, 93.0, 19.0, 11.9.

((2-Bromo-4-iodophenyl)ethynyl)triisopropylsilane (5)

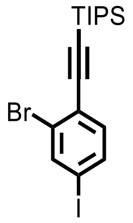

A solution of $NaNO_2$ (775.3 mg, 11.24 mmol) in water (6 mL) was added dropwise to a mixture of compound **4** (3.6 g, 10.22 mmol) in water (50 mL), MeCN (50 mL) and concentrated hydrochloric acid (3.96 mL) below 5 °C. After stirring for 10 min, a solution of potassium iodide (2.54 g, 15.32 mmol) in water (6 mL) was added dropwise. Then the reaction mixture was stirred at r.t. for 15 min, at 50 °C for 15 min and at 80 °C for 15 min. After reaction, the mixture was cooled to 0 °C, and a solution of saturated $Na_2S_2O_5$ was added. Afterward, the reaction mixture was extracted three times with DCM, washed with brine and dried over $MgSO_4$. The solvent was removed under reduced pressure and the residue was purified by column chromatography on silica gel with *iso*-hexane to afford compound **5** (2.46 g, 57%). MS (ESI, m/z): calcd for $[M+CH_2OH]^+$, 493.0059; observed 493.1201. ^1H NMR (300 MHz, CD_2Cl_2): 7.97 (d, 1.7 Hz, 1H), 7.61 (dd, 8.2 Hz, 1.7 Hz, 1H), 7.23 (d, 8.2 Hz, 1H), 1.17 (s, 21H). ^{13}C NMR (75 MHz, CD_2Cl_2): 140.9, 136.6, 135.0, 126.6, 125.6, 104.4, 98.5, 94.3, 19.0, 11.7.

((3,3''-Dibromo-[1,1':3',1''-terphenyl]-4,4''-diyl)bis(ethyne-2,1-diyl))bis(triisopropylsilane) (6)

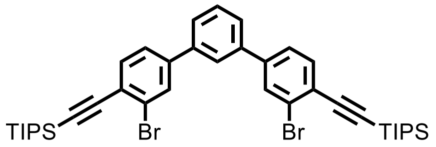

A solution of compound **5** (633.07 mg, 1.37 mmol), 1,3-bis(4,4,5,5-tetramethyl-1,3,2-dioxaborolan-2-yl)benzene (205 mg, 621.14 µmol) and K_2CO_3 (515.07 mg, 3.73 mmol) in THF (20 mL), EtOH (5 mL) and water (5 mL) was purged with argon for 20 min. Then to this solution was added $Pd(PPh_3)_4$ (71.78 mg, 62.11 µmol). The reaction mixture was stirred at 60 °C for 2 days. Afterward, the reaction mixture was extracted three times with DCM, washed with brine and dried over $MgSO_4$. The solvent was removed under reduced pressure and the residue was purified by column chromatography on silica gel with *iso*-hexane to afford compound **6** (338.71 mg, 73%). HRMS (APCI, m/z): calcd for $[M+H]^+$, 749.2032; observed 749.2019, error = -1.74 ppm. ^1H NMR (300 MHz, CD_2Cl_2): 7.90 (d, 1.6 Hz, 2H), 7.76–7.75 (m, 1H), 7.63–7.54 (7H), 1.20 (s, 42H). ^{13}C NMR (75 MHz, CD_2Cl_2): 142.5, 140.1, 134.5, 131.3, 130.1, 127.2, 126.5, 126.2, 126.0, 125.0, 105.1, 97.6, 18.9, 11.8.

14,34-Bis((triisopropylsilyl)ethynyl)-1,2,3,4,5,6(1,3)-hexabenzenacyclohexaphane (9-1)

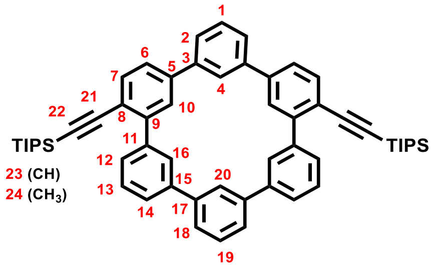

A solution of compound **6** (157 mg, 209.66 µmol), compound **8** (101.1 mg, 209.66 µmol) and $NaHCO_3$ (570.37 mg, 6.71 mmol) in THF (380 mL) and water (20 mL) was purged with argon for 40 min. Then to this solution were added $Pd_2(dba)_3$ (57.6 mg, 62.9 µmol) and [(*t*-Bu)$_3$PH]BF_4 (73.5 mg, 251.59 µmol). The reaction mixture was stirred at 80 °C for 3 days. Afterward, the organic solvent was removed under reduced pressure and the reaction mixture was extracted three times with DCM, washed with brine and dried over $MgSO_4$. The solvent was removed under reduced pressure and the residue was purified by column chromatography on silica gel with eluent (*iso*-hexane:DCM = 10:1). The crude product was further purified by recycling GPC to afford compound **9-1** as a white solid (20.55 mg, 12%). HRMS (APCI, m/z): calcd for $[M+H]^+$, 817.4625; observed 817.4596, error = -3.55 ppm. ^1H NMR (500 MHz, CD_2Cl_2): 8.25 (m, 2H; 12), 8.23 (m, 1H; 4), 8.22 (m, 1H; 20), 8.15 (m, 2H; 16), 8.14 (d, 1.8 Hz, 2H; 10), 7.77 (dd, 7.7 Hz, 1.7 Hz, 2H; 18), 7.75 (d, 8.0 Hz, 2H; 7), 7.75 (dd, 7.7 Hz, 1.7 Hz, 2H; 2), 7.71 (m, 2H; 14), 7.65 (dd, 8.0 Hz, 1.8 Hz, 2H; 6), 7.61 (t, 7.7 Hz, 1H; 19), 7.59 (t, 7.7 Hz, 1H; 1), 7.51 (t, 7.6 Hz, 2H; 13), 1.15 (42H; 23, 24). ^{13}C NMR (75 MHz, CD_2Cl_2): 143.5 (9), 141.7 (17), 141.3 (15), 141.2 (5), 140.9 (3), 140.7 (11), 135.4 (2), 130.0 (1), 129.9 (19), 129.1 (12), 129.0 (10), 128.5 (13), 128.3 (16), 127.7 (20), 127.0 (4), 126.4 (14), 126.2 (7), 125.7 (6, 18), 120.8 (8), 106.7 (21), 95.8 (22), 11.8 (23), 18.8 (24).

14,34-Diethynyl-1,2,3,4,5,6(1,3)-hexabenzenacyclohexaphane (9)

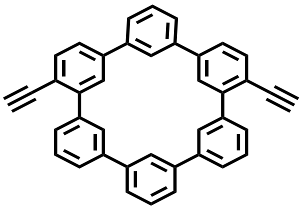

A solution of THF (10 ml) and compound **9-1** (16 mg, 19.58 µmol) was bubbled with argon for 5 min, then 0.1 mL of TBAF (1 M in the THF) was added dropwise. After 20 min, the reaction was quenched with methanol and the solvent was removed under reduced pressure. The residue was precipitated from DCM/MeOH to afford compound **9** as a white solid (8.5 mg, 86%). The poor solubility of this compound prohibited NMR and mass characterizations.

((2',4',5',6'-Tetraphenyl-[1,1':3',1''-terphenyl]-3,3''-diyl)bis(ethyne-2,1-diyl))bis(triisopropylsilane)/((2',3',5',6'-tetraphenyl-[1,1':4',1''-terphenyl]-3,3''-diyl)bis(ethyne-2,1-diyl))bis(triisopropylsilane) (10-1, isomeric mixture)

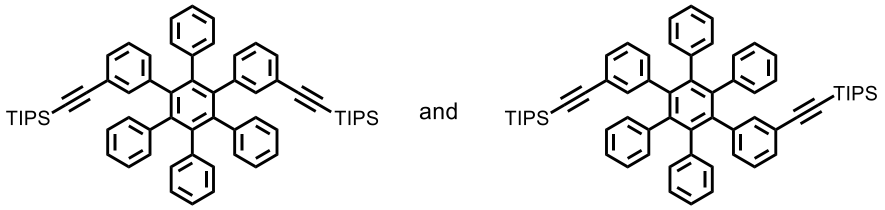

In a 20 mL Schleck tube, a degassed solution of compound **18** (401.7 mg, 711.17 μmol) and compound **19** (637.56 mg, 1.18 mmol) in diphenyl ether (5 mL) was refluxed for 24 h. After cooling to r.t., MeOH was added. The resulting crude product was collected by filtration and further purified by column chromatography on silica gel with eluent (*iso*-hexane:DCM = 10:1) to afford compound **10-1** (355.1 mg, 56%). HRMS (ESI, m/z): calcd for $[M+Na]^+$, 917.4914; observed 917.4893, error = -2.29 ppm. ^{1}H NMR (300 MHz, CD_2Cl_2): 7.71–6.82 (28H), 1.17 (s, 42H). ^{13}C NMR (75 MHz, CD_2Cl_2): 141.1, 141.1, 141.0, 140.9, 140.8, 140.0, 139.9, 136.0, 131.9, 131.7, 128.9, 127.3, 127.2, 127.0, 126.0, 122.2, 107.7, 90.2, 18.9, 11.8.

3-Ethynyl-3'-(3-ethynylphenyl)-4',5',6'-triphenyl-1,1':2',1''-terphenyl/3-ethynyl-4'-(3-ethynylphenyl)-3',5',6'-triphenyl-1,1':2',1''-terphenyl (10, isomeric mixture)

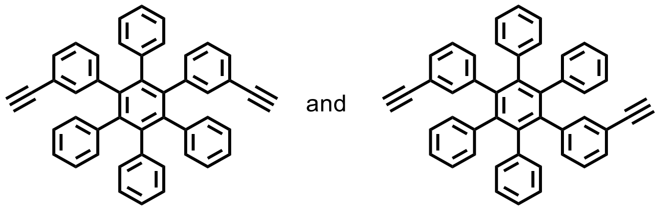

A solution of THF (40 ml) and compound **10-1** (448 mg, 500.32 μmol) was bubbled with argon for 30 min, then 1.5 mL of TBAF (1 M in the THF) was added dropwise. After 20 min, the reaction was quenched with methanol and the solvent was removed under reduced pressure. The crude product was diluted with DCM and washed with water and brine, then dried over Mg_2SO_4. The organic solvent was concentrated under reduced pressure and the residue was purified by column chromatography on silica gel with eluent (*iso*-hex:DCM = 1:1) to afford compound **10** as a white solid (256.6 mg, 88%). HRMS (ESI, m/z): calcd for $[M+H]^+$, 583.2426; observed 583.2424, error = -0.34 ppm. ^{1}H NMR (300 MHz, CD_2Cl_2): 7.05–6.84 (28H), 2.97 (s, 2H). ^{13}C NMR (75 MHz, CD_2Cl_2): 141.4, 141.3, 141.0, 140.9, 140.8, 140.7, 140.7, 140.6, 140.6, 140.4, 139.9, 139.7, 135.2, 135.1, 132.4, 131.7, 131.6, 131.6, 129.6, 127.2, 127.1, 127.0, 126.0, 125.9, 125.7, 120.8, 83.8, 76.9.

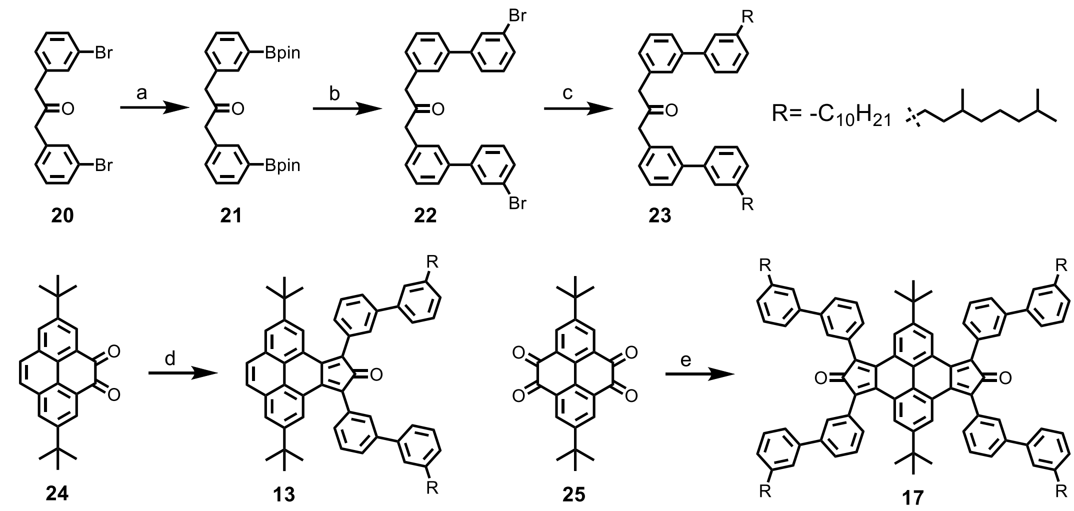

Scheme 2. Synthetic route toward compounds **13** and **17**. Reagents and conditions: (a) B_2pin_2, $Pd(dppf)Cl_2{\cdot}CH_2Cl_2$, KOAc, dioxane, 90 °C, 24 h, 81%; (b) 3-bromoiodobenzene, $Pd(PPh_3)_4$, K_2CO_3, toluene/EtOH/H_2O, 90 °C, 24 h, 80%; (c) 1-bromo-3,7-dimethyloctane, Zn, I_2, 80 °C, 24 h, $Pd(dppf)Cl_2$, r.t., 24 h, 85%; (d) **23**, KOH, EtOH, 78 °C, 15 min, 49%; (e) **23**, DBU, EtOH, 78 °C, 50 min, 12%.

1,3-Bis(3-(4,4,5,5-tetramethyl-1,3,2-dioxaborolan-2-yl)phenyl)propan-2-one (21)

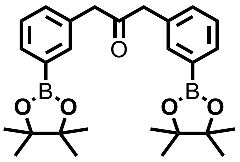

To a mixture of compound **20** (4 g, 10.87 mmol), B_2pin_2 (8.28 g, 32.6 mmol), KOAc (6.4 g, 65.21 mmol), and $Pd(dppf)Cl_2{\cdot}CH_2Cl_2$ (887.49 mg, 1.09 mmol) was added degassed anhydrous dioxane (100 mL). The reaction mixture was stirred at 90 °C for 24 h under argon atmosphere. After cooling to r.t., the reaction mixture was passed through glass filter to remove insoluble moiety and the solvent was removed under reduced pressure. Afterward, the residue was diluted, extracted three times with DCM, washed with brine and dried over $MgSO_4$. The solvent was removed under reduced pressure and the residue was purified by column chromatography on silica gel with eluent (*iso*-hexane:EA = 20:1–10:1) to afford compound **21** (4.07 g, 81%). HRMS (ESI, m/z): calcd for $[M+Na]^+$, 485.2647; observed 485.2646, error = -0.21 ppm. ^1H NMR (300 MHz, CD_2Cl_2): 7.67 (d, 7.2 Hz, 1H), 7.57 (s, 1H), 7.34 (m, 1H), 7.26 (d, 7.7 Hz, 1H), 3.78 (s, 2H), 1.34 (s, 12H). ^{13}C NMR (75 MHz, CD_2Cl_2): 205.8, 136.3, 134.0, 133.6, 132.8, 128.4, 84.3, 49.5, 25.1.

1,3-Bis(3'-bromo-[1,1'-biphenyl]-3-yl)propan-2-one (22)

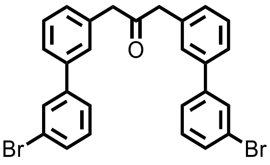

A solution of compound **21** (847.5 mg, 1.83 mmol), 3-bromoiodobenzene (1.82 g, 6.42 mmol) and K_2CO_3 (1.39 g, 10.08 mmol) in toluene (60 mL), EtOH (15 mL) and water (15 mL) was purged with argon for 30 min. Then to this solution was added $Pd(PPh_3)_4$ (211.89 mg, 183.36 µmol). The reaction mixture was stirred at 90 °C for 20 h. Afterward, the reaction mixture was extracted three times with DCM, washed with brine and dried over $MgSO_4$. The solvent was

removed under reduced pressure and the residue was purified by column chromatography on silica gel with eluent (*iso*-hexane:DCM = 1:1) to afford compound **22** (767.6 mg, 80%). HRMS (ESI, m/z): calcd for $[M+Na]^+$, 542.9758; observed 542.9753, error = -0.92 ppm. ^{1}H NMR (300 MHz, CD_2Cl_2): 7.71 (m, 2H), 7.51–7.47 (6H), 7.42 (m, 2H), 7.33–7.28 (4H), 7.21 (d, 7.3 Hz, 2H), 3.86 (s, 4H). ^{13}C NMR (75 MHz, CD_2Cl_2): 205.3, 143.3, 140.3, 135.3, 130.7, 130.7, 130.4, 129.6, 129.6, 128.7, 126.2, 126.1, 123.1, 49.6.

1,3-Bis(3'-(3,7-dimethyloctyl)-[1,1'-biphenyl]-3-yl)propan-2-one (23)

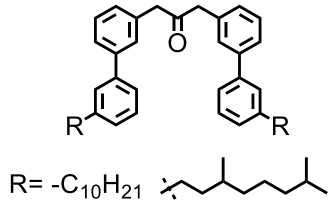

8 mL of DMA was bubbled for 10 min then Zn (3.46 g, 52.9 mmol) and I_2 (755.19 mg, 2.98 mmol) were added (slightly exothermal). The mixture was stirred at r.t. until the red color of I_2 disappeared. Afterwards, 1-bromo-3,7-dimethyloctane (5.85 g, 26.45 mmol) was added and the mixture was stirred at 80 °C for 24 h. After cooling to r.t., the solution of 1-bromo-3,7-dimethyloctanylzinc bromide was added via cannula to a mixture of compound **22** (1.72 g, 3.31 mmol) and $Pd(dppf)Cl_2{\cdot}CH_2Cl_2$ (539.96 mg, 661.2 µmol) in a 25 mL Schlenk flask. The reaction mixture was stirred at the room temperature for 24 h, then quenched by 1 N HCl. Afterward, the reaction mixture was extracted three times with DCM, washed with brine and dried over $MgSO_4$. The solvent was removed under reduced pressure and the residue was purified by column chromatography on silica gel with eluent (*iso*-hexane:DCM = 2:1) to afford compound **23** (1.81 g, 85%). HRMS (ESI, m/z): calcd for $[M+Na]^+$, 665.4698; observed 665.4692, error = -0.90 ppm. ^{1}H NMR (300 MHz, CD_2Cl_2): 7.57–7.55 (m, 1H), 7.47–7.32 (5H), 7.25–7.20 (m, 2H), 3.90 (s, 2H), 2.83–2.64 (m, 2H), 1.81–1.67 (m, 1H), 1.65–1.50 (m, 3H), 1.47–1.32 (m, 3H), 1.29–1.19 (m, 3H), 1.02 (d, 6.2 Hz, 3H), 0.95 (s, 3H), 0.93 (s, 3H). ^{13}C NMR (75 MHz, CD_2Cl_2): 205.6, 144.4, 142.2, 141.2, 135.2, 129.4, 129.1, 128.9, 128.9, 128.0, 127.7, 126.2, 124.8, 49.7, 39.8, 39.6, 37.6, 34.0, 33.1, 28.5, 25.2, 23.0, 22.9, 19.9.

2,7-Di-tert-butyl-9,11-bis(3'-(3,7-dimethyloctyl)-[1,1'-biphenyl]-3-yl)-10H-cyclopenta[e]pyren-10-one (13)

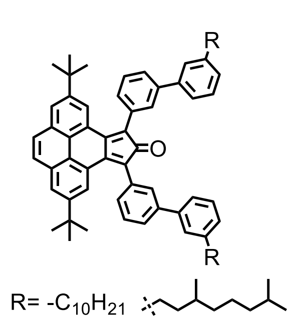

Compound **23** (92.4 mg, 143.71 µmol) and compound **24** (45 mg, 130.64 µmol) were suspended in 8 mL ethanol under an Ar. A solution of KOH (10.21 mg, 182.03 µmol) in 1 mL ethanol was added dropwise at 78 °C and the reaction mixture was stirred for 15 minutes. Afterward, the reaction mixture was cooled down to 0 °C in ice bath, the solvent was removed under reduced pressure and the residue was purified by column chromatography on silica gel with eluent (*iso*-hexane:DCM = 3:1) to afford compound **13** as dark brown solid (60.3 mg, 49%). HRMS (MALDI-TOF, m/z): calcd for $[C_{71}H_{82}O]^+$, 950.6366; observed 950.6367, error = 0.1 ppm. ^{1}H NMR (300 MHz, CD_2Cl_2): 7.94 (d, 1.9 Hz, 1H), 7.71–7.68 (3H), 7.65–7.60 (m, 2H), 7.46–7.43

(m, 3H), 7.34 (t, 7.4 Hz, 1H), 7.18 (d, 7.5 Hz, 1H), 2.71–2.57 (m, 2H), 1.71–1.60 (m, 1H), 1.52–1.44 (3H), 1.31–1.27 (3H), 1.17–1.12 (m, 3H), 1.08 (s, 9H), 0.93 (d, 6.3 Hz, 3H), 0.85 (d, 6.6 Hz, 6H). ^{13}C NMR (75 MHz, CD_2Cl_2): 201.1, 150.2, 149.1, 144.4, 142.6, 141.2, 134.4, 132.5, 129.8, 129.1, 129.0, 128.0, 127.8, 127.7, 127.5, 127.3, 127.1, 127.0, 125.2, 124.8, 124.0, 39.8, 39.6, 37.6, 34.9, 34.0, 33.0, 31.0, 28.4, 25.1, 22.9, 22.8, 19.8.

2,8-Di-tert-butyl-4,6,10,12-tetrakis(3'-(3,7-dimethyloctyl)-[1,1'-biphenyl]-3-yl)dicyclopenta[e,l]pyrene-5,11-dione (17)

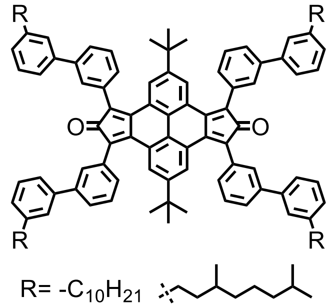

To a suspension of compound **23** (514.67 mg, 800.4 μmol) and compound **25** (135 mg, 360.54 μmol) in degassed ethanol DBU (192.11 mg, 1.26 mmol) was added dropwise under argon atmosphere. The mixture was stirred at 78 °C for 45 min then cooled down to 0 °C in ice bath. The resulted solid was collected by filtration, washed with cold ethanol (5 mL) and dried under vacuum. The crude product was further purified by column chromatography on silica gel with eluent (*iso*-hexane:ethyl acetate = 20:1) to afford compound **17** as a dark green solid (71 mg, 12%). HRMS (MALDI-TOF, m/z): calcd for $[C_{118}H_{138}O_2]^+$, 1588.0730; observed 1588.0771, error = 2.58 ppm. ^{1}H NMR (300 MHz, CD_2Cl_2): 7.65–7.57 (4H), 7.54 (d, 7.7 Hz, 2H), 7.44 (s, 1H), 7.42 (t, 1.7 Hz, 2H), 7.39 (t, 1.6 Hz, 1H), 7.37–7.27 (6H), 7.19 (dt, 7.5 Hz, 1.6 Hz, 2H), 2.74–2.57 (m, 4H), 1.74–1.60 (m, 2H), 1.55–1.45 (6H), 1.37–1.31 (6H), 1.18–1.12 (m, 6H), 0.95 (d, 6.1 Hz, 6H), 0.90–0.83 (21H). ^{13}C NMR (75 MHz, CD_2Cl_2): 200.7, 151.6, 148.2, 144.4, 142.6, 141.0, 133.7, 129.7, 129.6, 129.1, 129.0, 128.9, 128.8, 128.0, 127.6, 127.4, 124.8, 124.0, 39.8, 39.5, 37.6, 34.0, 33.0, 30.2, 30.1, 28.4, 25.1, 22.9, 22.8, 19.8.

HR MALDI-MS spectra of 1, 2, and 3.

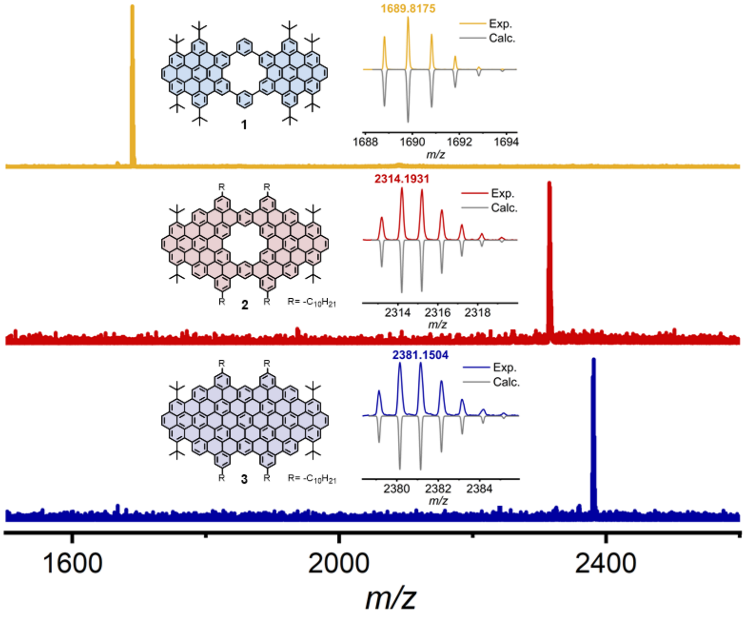

Figure S1. HR MALDI-TOF MS spectra of **1**, **2**, and **3**.

Compound 12

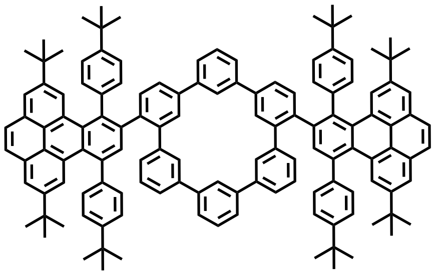

In a 10 mL Schleck tube, a degassed solution of compound **11** (21 mg, 33.29 µmol) and compound **9** (7 mg, 13.87 µmol) in diphenyl ether (1 mL) was refluxed for 24 h. After cooling to r.t., MeOH was added. The resulting residue was purified by column chromatography on silica gel with eluent (*iso*-hexane:DCM = 5:2) to afford compound **12** (21.8 mg, 92%). HRMS (MALDI-TOF, m/z): calcd for $[M]^+$, 1708.9703; observed 1708.9709, error = 0.35 ppm. Compound **12** is a mixture of isomers. The EXSY spectrum shows that exchange processes take place and contribute to the complexity of the spectra (Figure S47). Both the ^{1}H and ^{13}C NMR spectra are characterized by a large number of overlapping signals in both the aromatic and aliphatic protons and carbons region. This makes it difficult to list the ^{1}H and ^{13}C NMR signals in a meaningful way. For this reason, both the overview spectrum and the region of aromatic protons/carbons are shown for reference (Figures S46 and S48). Furthermore, the most intense ^{13}C NMR signals are listed. The HSQC spectrum allows the identification of the aromatic CH carbons (Figure S49). ^{13}C NMR (125 MHz, CD_2Cl_2): 150.3, 149.1, 147.4, 147.0, 142.8, 142.7, 141.8, 141.5, 141.4, 141.2, 140.8, 140.7, 140.5, 140.0, 139.7, 139.5, 139.4, 139.2, 138.1, 133.8, 133.6, 133.1, 133.0, 131.5, 131.3, 131.1, 130.7, 130.6, 129.9, 129.6, 129.5, 128.9, 128.8, 128.7, 128.0, 127.8, 127.3, 127.1, 127.0, 126.6, 126.4, 126.2, 125.5, 125.4, 125.2, 125.1, 124.8, 123.5, 123.3, 122.2, 122.1, 37.4, 35.0, 34.9, 34.8, 34.2, 31.5, 31.4, 30.9, 30.0.

Compound 14

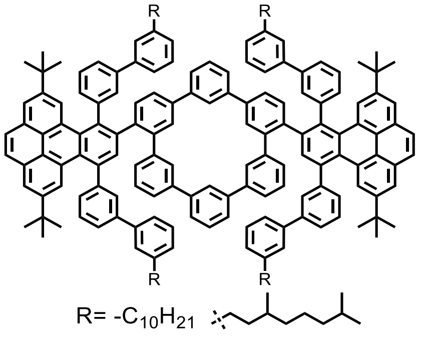

In a 10 mL Schleck tube, a degassed solution of compound **13** (36.97 mg, 38.86 µmol) and compound **9** (5.3 mg, 10.5 µmol) in diphenyl ether (0.5 mL) was refluxed for 24 h. After cooling to r.t., MeOH was added. The resulting residue was purified by column chromatography on silica gel with eluent (*iso*-hexane:DCM = 5:2) to afford compound **14** (22.2 mg, 90%). HRMS (MALDI-TOF, m/z): calcd for $[M]^+$, 2349.4711; observed 2349.4719, error = 0.34 ppm. Compound **14** is a mixture of isomers. Both the ^{1}H and ^{13}C NMR spectra are characterized by a large number of overlapping signals in both the aromatic and aliphatic protons and carbons region. This makes it difficult to list the ^{1}H and ^{13}C NMR signals in a meaningful way. For this reason, both the overview spectrum and the region of aromatic protons / carbons are shown for reference (Figures S50 and S52). The EXSY spectrum shows that exchange processes take

place (rotations about single bonds) and contribute to the complexity of the spectra (Figure S51). The HSQC spectrum allows the identification of the aromatic CH carbons (Figure S53).

Compound 15 (isomeric mixture)

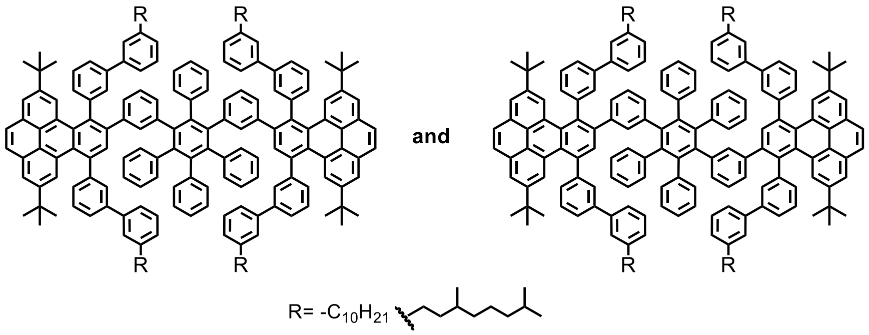

In a 10 mL Schleck tube, a degassed solution of compound **13** (74.71 mg, 76.28 µmol) and compound **10** (12.7 mg, 21.79 µmol) in diphenyl ether (0.5 mL) was refluxed for 24 h. After cooling to r.t., MeOH was added. The resulting residue was purified by column chromatography on silica gel with eluent (*iso*-hexane:DCM = 5:2) to afford compound **15** (46 mg, 87%). HRMS (MALDI-TOF, m/z): calcd for $[C_{186}H_{194}]^+$, 2429.5248; observed 2429.5215, error = -1.36 ppm. Compound **15** is a mixture of isomers. Both the ^{1}H and ^{13}C NMR spectra are characterized by a large number of overlapping signals in both the aromatic and aliphatic protons and carbons region. This makes it difficult to list the ^{1}H and ^{13}C NMR signals in a meaningful way. For this reason, both the overview spectrum and the region of aromatic protons / carbons are shown for reference (Figures S54 and S55).

Model compound 1

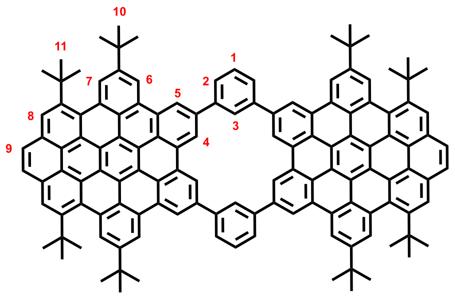

Compound **12** (11.1 mg, 6.49 µmol) was dissolved in degassed dry DCM (10 mL) in a 25 mL Schlenk flask equipped with a magnetic stirrer under Ar atmosphere. To the solution was added a suspension of iron(III) chloride $FeCl_3$ (252.62 mg, 1.56 mmol) in degassed nitromethane (2 mL). After stirring at room temperature for 1 h, the reaction was quenched by addition of MeOH (10 mL) and the precipitation was filtered to get the crude product. Afterward, the residue was purified by column chromatography on silica gel with *iso*-hex:DCM 5:2 to give compound **1** as a yellow solid (9.8 mg, 90%). HRMS (MALDI-TOF, m/z): calcd for $[C_{132}H_{104}]^+$, 1689.8173; observed 1689.8175, error = 0.12 ppm. ^{1}H NMR (500 MHz, CD_2Cl_2/CS_2 1/1 v/v): 10.30 (s, 4H; 4), 9.81 (s, 4H; 5), 9.50 (s, 4H; 6), 9.36 (s, 2H; 3), 9.17 (s, 4H; 8), 9.16 (s, 4H; 7), 8.62 (s, 4H; 9), 8.42 (d, 8.0 Hz, 4H; 2), 8.01 (t, 8.0 Hz, 2H; 1). ^{13}C NMR (75 MHz, CD_2Cl_2/CS_2 1/1 v/v): 145.9, 142.5, 139.3, 132.6, 132.0, 131.7, 130.7, 130.6 (7), 130.5 (1), 130.4, 130.1, 129.7, 129.5, 129.3, 129.1 (8), 128.8 (3), 127.5 (9), 127.4, 126.9 (2), 125.9, 125.6, 124.5, 122.1 (4), 121.8, 121.2, 121.1, 120.6 (5), 120.5, 119.6 (6), 37.7, 36.2, 35.4 (11), 32.5 (10). Note: The assignment of the CH and CH_3 signal is based on the HSQC spectrum.

Model compound 2

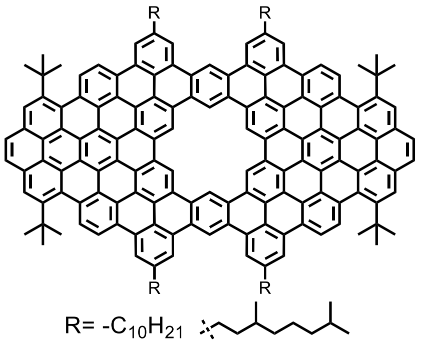

Compound **14** (9.8 mg, 4.17 μmol) was dissolved in degassed dry DCM (10 mL) in a 25 mL Schlenk flask equipped with a magnetic stirrer under Ar atmosphere. To the solution was added a suspension of iron(III) chloride $FeCl_3$ (292.02 mg, 1.8 mmol) in degassed nitromethane (2 mL). After stirring at room temperature for 2 h, the reaction was quenched by addition of MeOH and the solvent was evaporated under reduced pressure. The obtained residue was further washed by excess of water and MeOH for 5 cycles, to afford compound **2** (9.1 mg, 94%) as an orange solid. HRMS (MALDI-TOF, m/z): calcd for $[C_{180}H_{152}]^+$, 2314.1929; observed 2314.1931, error = 0.08 ppm. The proton NMR spectrum of compound **2** could not be obtained, due to its strong aggregation and poor solubility in commonly used chlorinated NMR solvents, even at elevated temperatures (80 °C).

Model compound 3

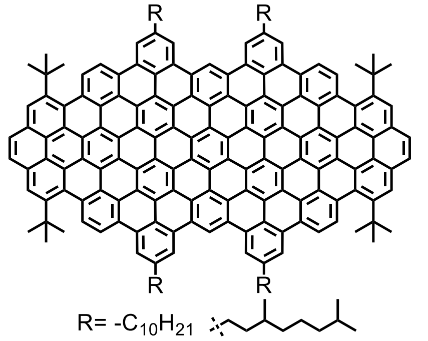

Compound **15** (27 mg, 11.11 μmol) was dissolved in degassed dry DCM (100 mL) in a Schlenk flask equipped with a magnetic stirrer under Ar atmosphere. To the solution was added a suspension of iron(III) chloride $FeCl_3$ (432.59 mg, 2.67 mmol) in degassed nitromethane (4 mL). After stirring at room temperature for 2 h, the reaction was quenched by addition of MeOH and the solvent was evaporated under reduced pressure. The obtained residue was further washed by excess of water and MeOH for 5 cycles, to afford compound **3** (24.2 mg, 92%) as a purple solid. HRMS (MALDI-TOF, m/z): calcd for $[C_{186}H_{146}]^+$, 2381.1492; observed 2381.1504, error = 0.50 ppm. Similar to compound **2**, a proton NMR spectrum of **3** could not be recorded due to its poor solubility and strong aggregation in commonly used chlorinated NMR solvents.

Synthesis of polymer **P1**

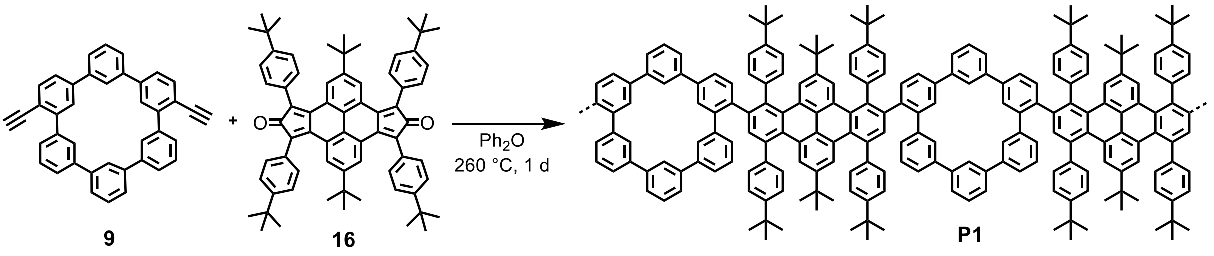

In a 10 mL Schleck tube, a degassed solution of compound **9** (17.1 mg, 33.89 μmol) and compound **16** (32.1 mg, 33.89 μmol) in diphenyl ether (0.4 mL) was refluxed for 24 h. After cooling to r.t., MeOH was added. 46.4 mg of precipitates were collected by filtration to obtain a crude polymer as a pale-yellow solid with the yield of 96%. The crude polymer was fractionalized to remove short polymers by using recycling GPC ($CHCl_3$ as eluent) and the resulted **P1** was characterized with analytical SEC.

(a)

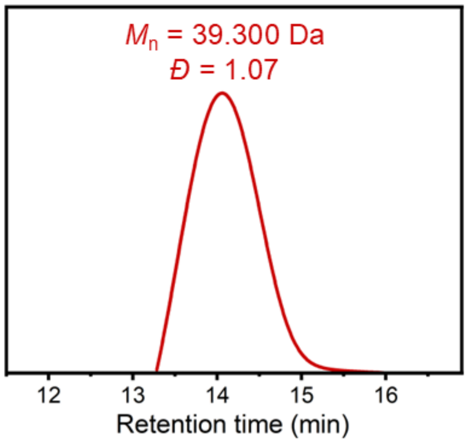

(b)

M_n (Da)	M_w (Da)	*Đ*	$\overline{DP}_n$	Length of GNR (nm)
39.300	42.200	1.07	28	60

Figure S2. SEC fractionation of polymer **P1**. (a) GPC curve of **P1**. (b) SEC results of **P1** (polystyrene calibration). The number-average degree of polymerization $\overline{DP}_n$ was calculated from M_n and the molecular weight of the repeat unit (1396 g/mol). The length of resultant **pGNR 1** was estimated from the number and the length of the repeat unit.

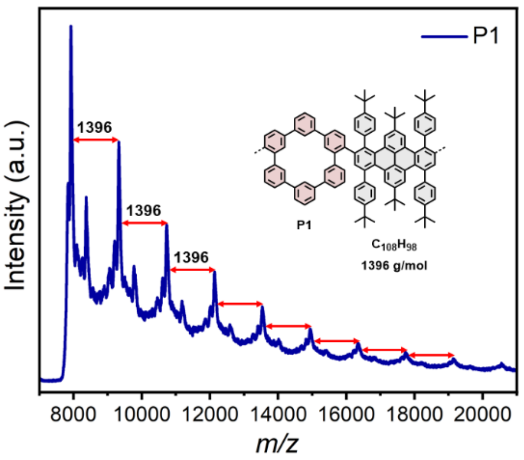

Figure S3. MALDI-TOF MS analysis of polymer **P1** (matrix: DCTB, linear mode).

Synthesis of polymer **P2**

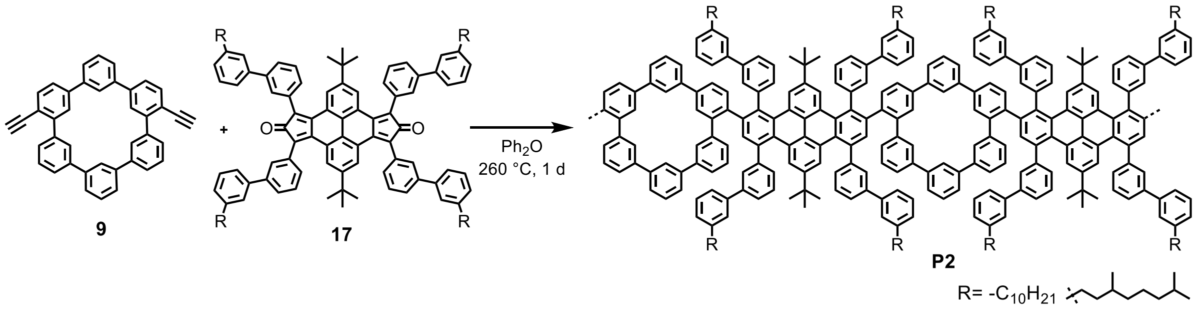

In a 10 mL Schleck tube, a degassed solution of compound **9** (20 mg, 39.63 μmol) and compound **17** (62.9 mg, 39.63 μmol) in diphenyl ether (0.4 mL) was refluxed for 24 h. After cooling to r.t., MeOH was added. 55.4 mg of precipitates were collected by filtration to obtain a crude polymer as a pale-yellow solid with the yield of 93%. The crude polymer was fractionalized to remove short polymers by using recycling GPC ($CHCl_3$ as eluent) and the resulted **P2** was characterized with analytical SEC.

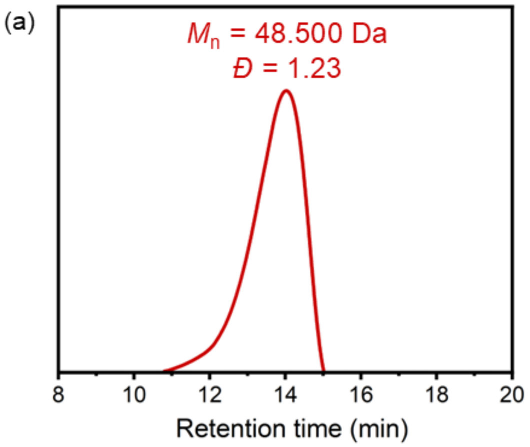

(b)

M_n (Da)	M_w (Da)	$Đ$	$\overline{DP}_n$	Length of GNR (nm)
48.500	59.900	1.23	24	50

Figure S4. SEC fractionation of polymer **P2**. (a) GPC curve of **P2**. (b) SEC results of **P2** (polystyrene calibration). The number-average degree of polymerization $\overline{DP}_n$ was calculated from M_n and the molecular weight of the repeat unit (2037 g/mol). The length of resultant **pGNR 2** was estimated from the number and the length of the repeat unit.

Synthesis of polymer **P3**

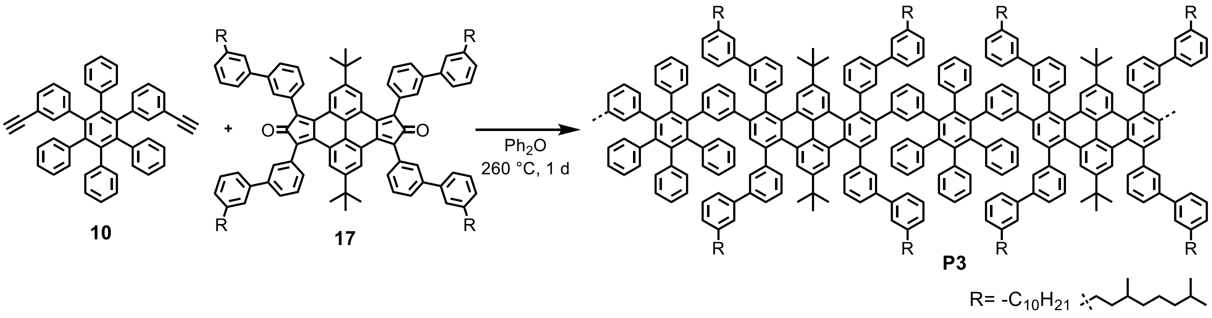

In a 10 mL Schleck tube, a degassed solution of compound **10** (25.5 mg, 43.76 μmol) and compound **17** (69.51 mg, 43.76 μmol) in diphenyl ether (0.4 mL) was refluxed for 24 h. After cooling to r.t., MeOH was added. 65.7 mg of precipitates were collected by filtration to obtain a crude polymer as a pale-yellow solid with the yield of 95%. The crude polymer was fractionalized to remove short polymers by using recycling GPC ($CHCl_3$ as eluent) and the resulted **P3** was characterized with analytical SEC.

(a)

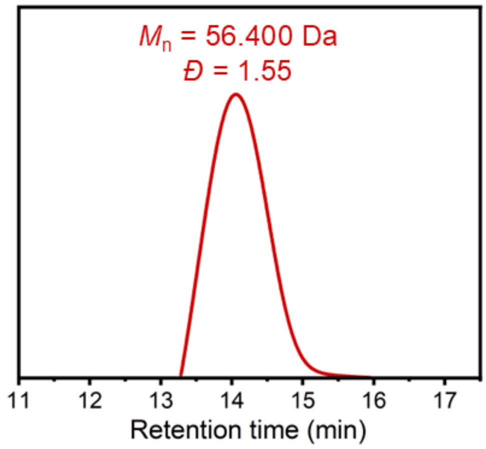

(b)

M_n (Da)	M_w (Da)	$Đ$	$\overline{DP}_n$	Length of GNR (nm)
56.400	87.500	1.55	26	55

Figure S5. SEC fractionation of polymer **P3**. (a) GPC curve of **P3**. (b) SEC results of **P3** (polystyrene calibration). The number-average degree of polymerization $\overline{DP}_n$ was calculated from M_n and the molecular weight of the repeat unit (2114 g/mol). The length of resultant **npGNR** was estimated from the number and the length of the repeat unit.

Synthesis of **pGNR 1**

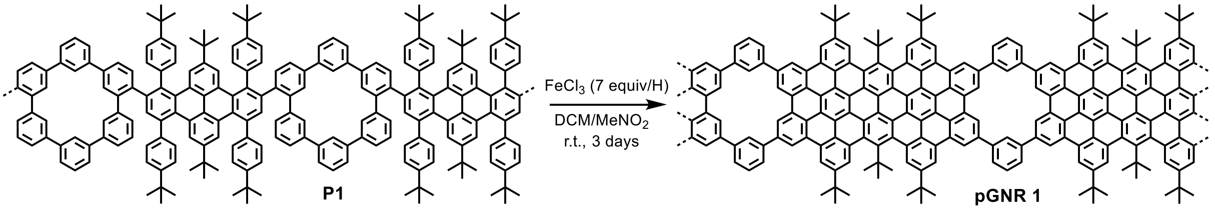

22.1 mg of **P1** was dissolved in 70 mL degassed DCM. Then the solution of iron(III) chloride (358.97 mg, 2.21 mmol, 7.0 equiv. for one hydrogen to be removed) in 5 mL nitromethane was added dropwise. Subsequently, a stream of Ar saturated with dichloromethane was passed through the mixture for 24 h. The mixture was stirred at room temperature for another 2 days. After the reaction, an excess amount of methanol was added to produce precipitate. The precipitate was collected by filtration, washed with water and methanol for 5 cycles. After drying under vacuum, 20.8 mg of **pGNR 1** was obtained as a black solid with the yield of 95%.

Synthesis of **pGNR 2**

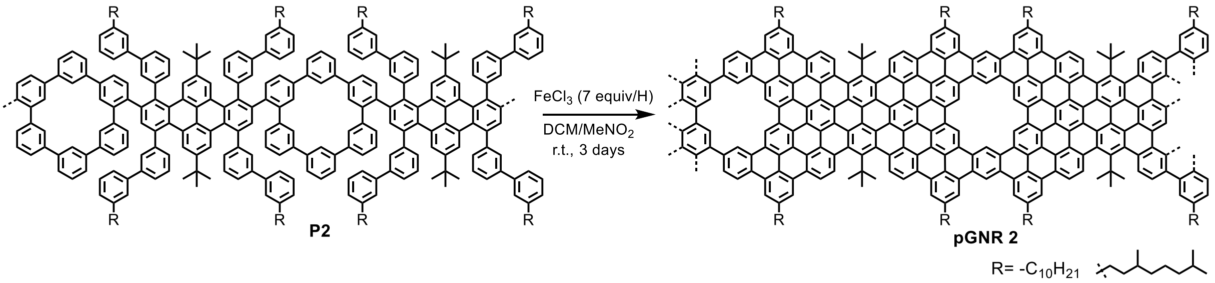

21.7 mg of **P2** was dissolved in 70 mL degassed DCM. Then the solution of iron(III) chloride (422.97 mg, 2.61 mmol, 7.0 equiv. for one hydrogen to be removed) in 4 mL nitromethane was added dropwise. Subsequently, a stream of Ar saturated with dichloromethane was passed through the mixture for 24 h. The mixture was stirred at room temperature for another 2 days. After the reaction, an excess amount of methanol was added to produce precipitate. The precipitate was collected by filtration, washed with water and methanol for 5 cycles. After drying under vacuum, 19.8 mg of **pGNR 2** was obtained as a black solid with the yield of 91%.

Synthesis of **npGNR**

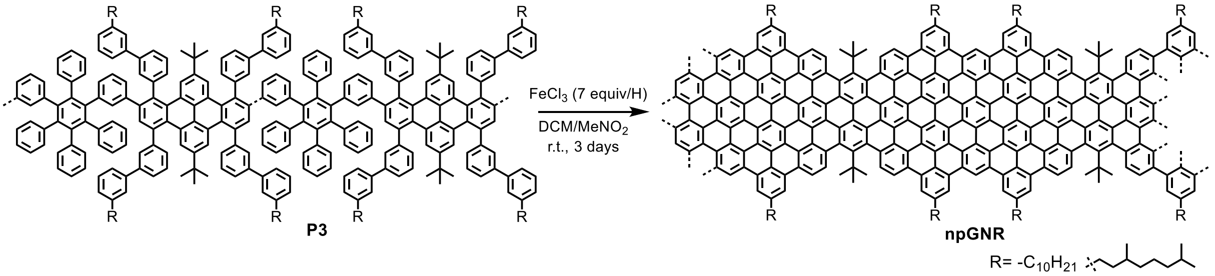

34.1 mg of **P3** was dissolved in 110 mL degassed DCM. Then the solution of iron(III) chloride (877.77 mg, 5.41 mmol, 7.0 equiv. for one hydrogen to be removed) in 8 mL nitromethane was added dropwise. Subsequently, a stream of Ar saturated with dichloromethane was passed through the mixture for 24 h. The mixture was stirred at room temperature for another 2 days. After the reaction, an excess amount of methanol was added to produce precipitate. The precipitate was collected by filtration, washed with water and methanol for 5 cycles. After drying under vacuum, 32.1 mg of **npGNR** was obtained as a black solid with the yield of 94%.

3. Crystallographic data of 1

Single crystals of compound **1** was obtained by slow vapor diffusion of methanol into a solution of compound **1** in 1,2-dichlorobenzene. The X-ray crystallographic coordinates for structure reported in this article have been deposited at the Cambridge Crystallographic Data Centre (CCDC), under deposition number CCDC 2354010. These data can be obtained free of charge from CCDC via https://www.ccdc.cam.ac.uk/structures/?

Table S1. Summary of crystal data and reflection collection parameters for model compounds **1**.

**Crystal**	**1**
**Moiety formula**	$C_{138}H_{108}Cl_2$
**Formula weight**	1837.14
**Crystal size, mm^3**	0.2 × 0.2 × 0.2
**Crystal system**	triclinic
**Space group**	P-1
***a*, Å**	16.9738(7)
***b*, Å**	17.7925(9)
***c*, Å**	24.3088(8)
**α, deg**	96.760(3)
**β, deg**	109.520(3)
**γ, deg**	95.904(4)
**Volume, Å^3**	6791.6(5)
Z	2
**$D_{calcd.}$, *g* cm^{-3}**	0.898
**F_{000}**	1940
***T*, K**	100.00(10)
**Radiation (λ, Å)**	CuKα (λ = 1.54184)
**μ, mm^{-1}**	0.735
**2Θ range (°)**	5.586 to 130.024
**Index ranges**	-19 ≤ h ≤ 19, -20 ≤ k ≤ 20, -28 ≤ l ≤ 26
**no. of collected reflections**	66752
**no. of unique ref. (R_{int})**	22200(0.0760)
**Data/restraints/parameters**	22200/112/1273
**R_1, wR_2 [obs I>2σ (I)]**	0.1137, 0.3033
**R_1, wR_2 (all data)**	0.1705, 0.3403
**residual peak/hole, e. Å^{-3}**	0.54/-0.36
**Goodness-of-fit on F^2**	1.243
**CCDC**	2354010

4. Optical properties of model compounds and GNRs

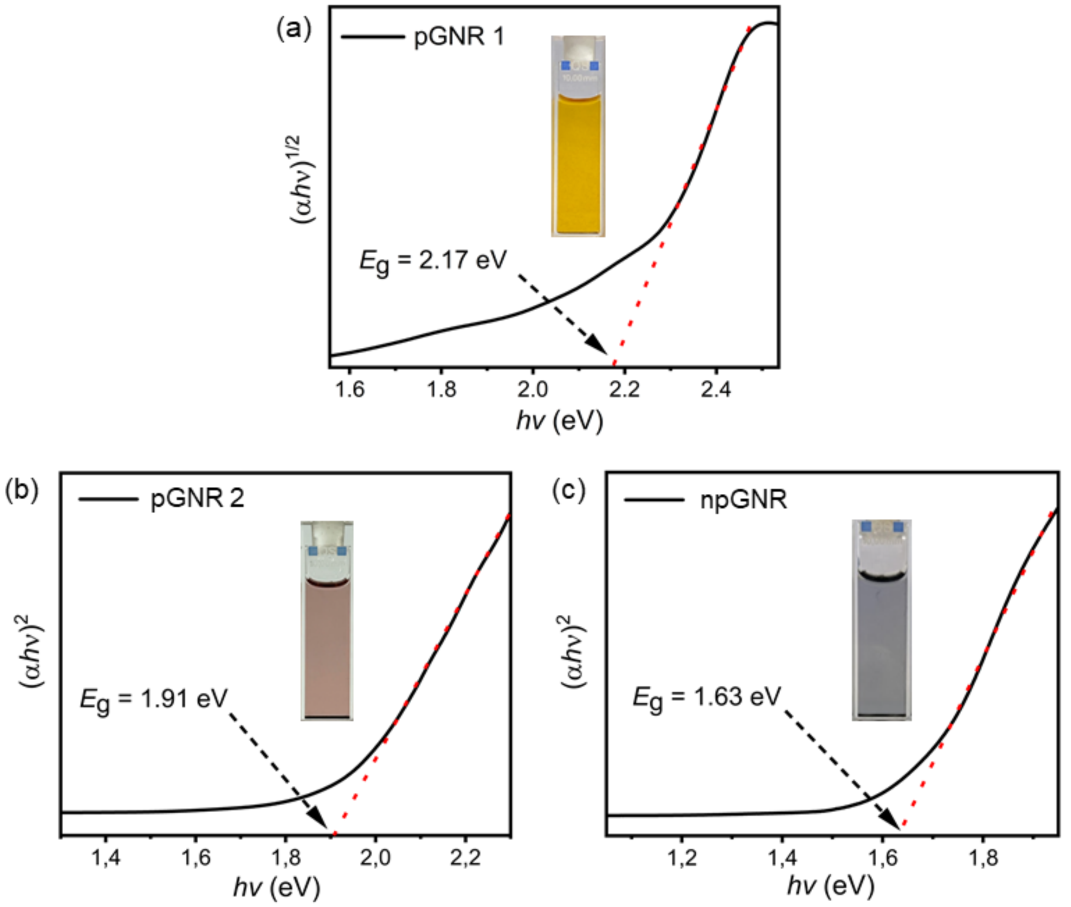

Figure S6. Tauc-plot of the UV-vis data of **pGNR 1**, **pGNR 2** and **npGNR**.

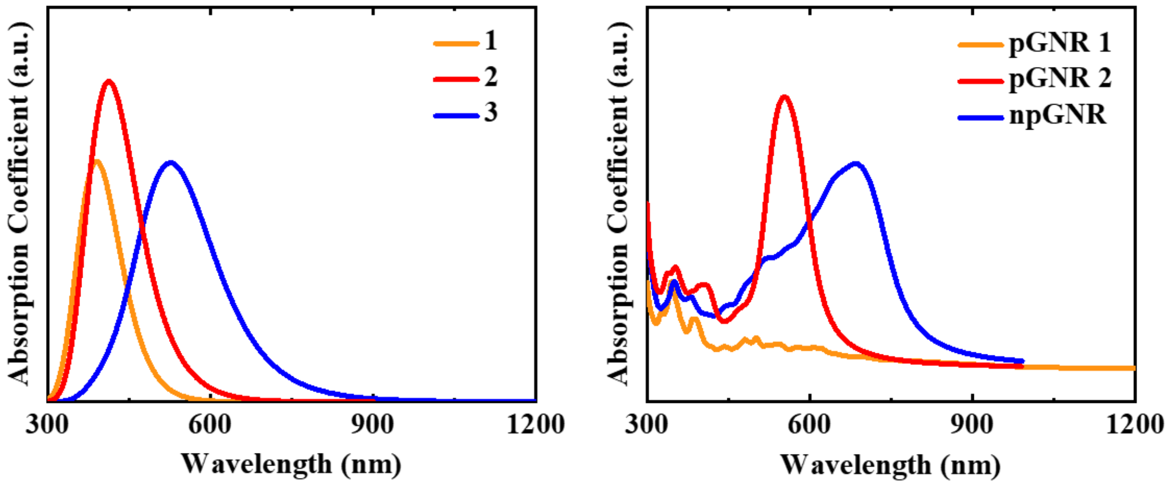

Figure S7. (a) DFT-calculated absorption spectra of compounds **1**, **2**, and **3**. (b) DFT-calculated absorption spectra of **pGNR 1**, **pGNR 2** and **npGNR**.

5. IR and Raman characterization

Experimental details. The reported Raman spectra of **2**, **pGNR 1**, and **pGNR 2** were acquired with the Labram HR800 instrument (Horiba-Jobin-Yvon) equipped with a 405 nm laser excitation. The powder samples of **pGNR 1**, **pGNR 2** and **2** were deposited on a metallic support and analyzed with the micro-Raman setup. The Raman spectra of **3** and **npGNR** were obtained with the same instrument and the 785 nm excitation provided by the Xtra solid-state laser produced by Toptica Photonics. The spectra were collected in backscattering geometry with a laser spot diameter of approximately 1 μm and a power at the sample of approximately 0.02 mW. The FT-IR spectra of compounds **2**, **3**, **pGNR 1**, **pGNR 2**, and **npGNR** were measured with a Nicolet Nexus equipment coupled with a Thermo-Nicolet Continuμm infrared microscope and a cooled MCT detector. The micro-FT-IR spectra were collected in transmission mode by depositing the powder sample obtained from the synthesis on a diamond anvil cell (4 cm^{-1} spectral resolution).

Computational details. Vibrational spectra have been predicted *in vacuo* for molecular models of **2** and **3** using the quantum chemical Gaussian09 code[7], while **pGNR 1**, **pGNR 2** and **npGNR** were modelled as one-dimensional periodic crystals within CRYSTAL17[8]. In all models, we have replaced the alkyl substituents with shorter chains (hexyl, ethyl, propyl, and propyl substituents in **2**, **pGNR 2**, **3**, and **npGNR**, respectively). Density functional theory (DFT) calculations were performed at the B3LYP/6-31G(d,p) level for **pGNR 1**, **2**, **pGNR 2**, and **3**; this is a widespread method for the computation of vibrational spectra of organic molecules, as the optimal trade-off between physical accuracy and computational efficiency. We modeled **npGNR** with a pure GGA functional since, unfortunately, SCF convergence could not be reached at the B3LYP/6-31G(d,p) level (this was probably due to the very small bandgap of **npGNR**). In particular, we adopted the PW91/6-31G(d,p) level for **npGNR**; both exchange and correlation functionals were set as PW91 using the PWGGA keyword of CRYSTAL17. As DFT at the B3LYP/6-31G(d,p) level is known to overestimate the wavenumbers of vibrational normal modes, the computed wavenumbers were scaled by numerical factors of 0.985 (for **2** and **pGNR 2**), and of 0.975 (for **3**), while they were kept as computed by DFT for **npGNR**. The indicated scale factors have been determined by visually adjusting to the observed spectrum the spectrum simulated from scaled DFT frequencies.

Table S2. Optimized DFT structures of **2**, **pGNR 1**, and **pGNR 2**. For **pGNR 1** and **pGNR 2**, which are periodic in one direction, three repeat units are shown.

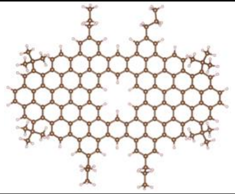	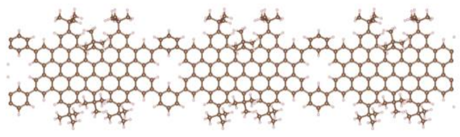	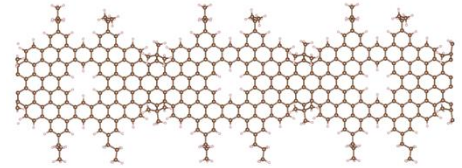
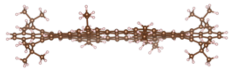	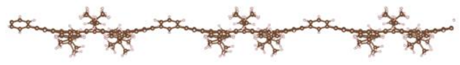	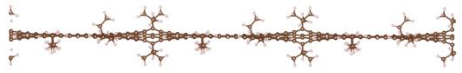
**2**	**pGNR 1**	**pGNR 2**

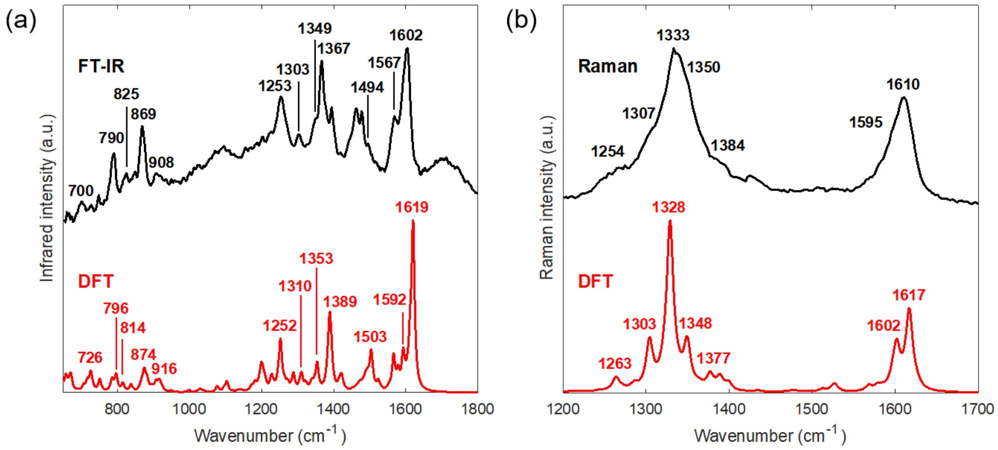

Figure S8. Normalized experimental (black line) and computed (red line) IR and Raman spectra of **pGNR 1**. The peak positions of the features assigned either in the main text or in the Supporting Information are labeled with their peak position (cm^{-1} units).

The experimental IR spectrum of **pGNR 1** shows two relatively intense bands at 790 and 869 cm^{-1} (Figure S8a), which are predicted at 796 and 874 cm^{-1} and assigned to TRIO and SOLO vibrations, respectively.[9] The low-intensity IR features measured at 908 and 1303 cm^{-1} are computed at 915 and 1310 cm^{-1} and are assigned to the out-of-plane and in-plane CH bending in the pore, respectively. Finally, the most intense feature observed at 1602 cm^{-1} (computed at 1619 cm^{-1}) is assigned to a ring-stretching mode of the mobile benzene ring located at the pore. The Raman spectrum of **pGNR 1** shows the characteristic D and G bands of molecular graphene (Figure S8b), and are nicely reproduced by DFT. The D peak of **pGNR 1** is observed at 1333 cm^{-1}, while the G peak is observed at 1610 cm^{-1}. As expected, the DFT calculations show that the D peak is assigned to collective ring-breathing vibrations of the graphene moiety, and the G peak is assigned to collective CC stretching vibrations of the graphene moiety.

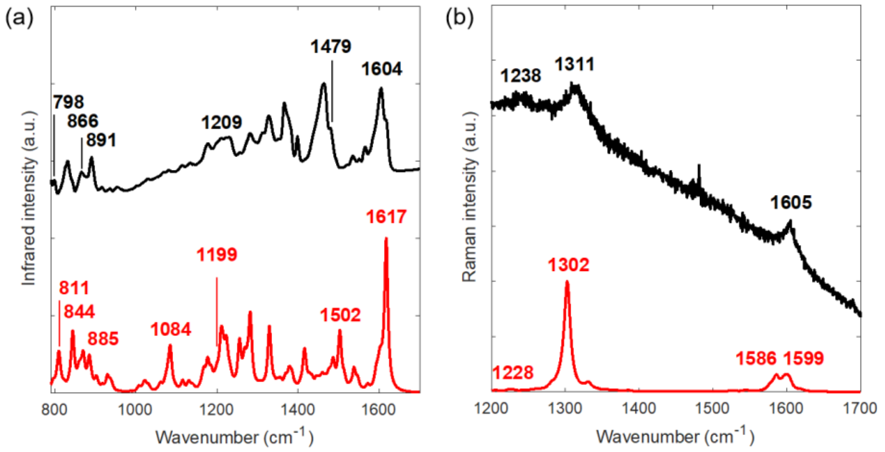

Figure S9. Experimental (black line) and computed (red line) IR and Raman spectra of **3**. Selected bands are labelled with their peak position in cm^{-1}.

Figure S9a shows the experimental and computed IR spectra of **3**. We assign the band measured at 798 cm^{-1} to the DUO vibrational mode, predicted at 811 cm^{-1} (unscaled value: 832 cm^{-1}; see SI). The band observed at 891 cm^{-1}, predicted at 885 cm^{-1} (unscaled value: 908 cm^{-1}), is assigned to the SOLO vibrational mode coupled with DUO vibration. DFT also allows us to assign the band measured at 1209 cm^{-1} to a CC stretching mode localized on the *tert*-butyl groups (predicted at 1199 cm^{-1}, unscaled value: 1230 cm^{-1}), as well as the experimental feature at 1604

cm^{-1} to the ring stretching of the rings functionalized with alkyl chains, predicted at 1617 cm^{-1} (unscaled value: 1658 cm^{-1}). Finally, the experimental band at 1463 cm^{-1} is predicted at 1468 cm^{-1} (unscaled value: 1506 cm^{-1}) and assigned to vibrational modes localized in the alkyl chains; since the alkyl chains in our model are shorter than in the real molecule, DFT underestimates the intensity of such band (see the SI for further details about the simplifications introduced in the computational models). The latter observation also applies to **npGNR**. The measured Raman spectrum of **3** is nicely reproduced by DFT, as shown in Figure S9b and further illustrated in Table S10. The D peak is measured at 1311 cm^{-1} and has its computational counterpart at 1302 cm^{-1} (unscaled value: 1335 cm^{-1}), corresponding to a vibrational mode assigned to collective ring breathing, as expected. On the other hand, the experimental G peak, measured at 1605 cm^{-1}, corresponds to a pair of computed collective CC stretching vibrational modes at 1586 (longitudinal) and 1599 (transversal) cm^{-1} (unscaled values: 1627 and 1640 cm^{-1}). DFT also allows to assign the less intense feature measured at 1238 cm^{-1} to a localized D-like vibrational mode computed at 1228 cm^{-1} (unscaled value: 1259 cm^{-1}).

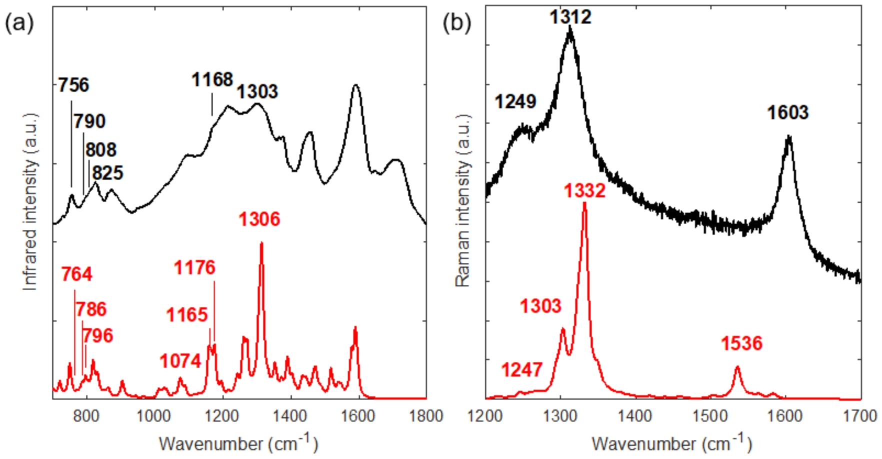

Figure S10. Experimental (black line) and computed (red line) IR and Raman spectra of **npGNR**. Selected bands are labelled with their peak position in cm^{-1}.

Concerning the IR spectrum of **npGNR**, shown in Figure S10a, we assign to DUO vibrational modes the two overlapping bands measured at 790 and 808 cm^{-1} (these bands are respectively computed at 786 and 796 cm^{-1}), and vibrations of the aromatic CH groups of the rings functionalized with alkyl chains to the band measured at 825 and computed at 818 cm^{-1}. Moreover, the feature observed at 1168 cm^{-1} corresponds to vibrations computed at 1165 cm^{-1} and 1176 cm^{-1} and assigned respectively to the CC stretching of *tert*-butyl substituents and to the in-plane vibrations of the CH groups in DUO position. Similar to **3**, the feature measured at 1456 cm^{-1} is assigned to CH bending modes localized in the alkyl chains, not intense in the DFT calculations due to the shorter alkyl chains used in our model to limit the computational effort. The intense band observed at 1303 cm^{-1} is assigned to the collective CC stretching mode, and computed at 1306 cm^{-1}. Finally, the band observed at 1710 cm^{-1} in the experimental spectrum can be explained as originating from the C=O stretching of residual terminal groups of the polymer. The experimental Raman spectrum of **npGNR**, shown in Figure S10b, has a D band and a G band at 1312 and 1603 cm^{-1}, respectively, as expected for GNRs. DFT calculations show that the D peak is a structured band assigned to two different localizations of ring-breathing vibrational modes, and allows to assign the less intense band at 1249 cm^{-1} to another D-like vibrational mode, predicted at 1247 cm^{-1}. It should be noticed that even though the chosen level of theory (see Computational Methods) underestimates the wavenumber of the G

band (computed at 1536 cm^{-1}), the spectral pattern is reasonably reproduced by the DFT calculation.

Table S3. Vibrational assignment of the IR spectrum of **pGNR 1**. The first two columns report the unscaled and scaled wavenumbers of the computed normal modes, respectively; the third column reports the peak positions of the experimental features assigned to the wavenumbers in the first two columns; the fourth column reports the computed IR intensities of the vibrational normal modes; finally, the fifth column reports the description of the vibrational normal modes.

**DFT wavenumber (cm^{-1})**	**Scaled DFT wavenumber (cm^{-1})**	**Experimental peak position (cm^{-1})**	**Computed infrared intensity (km/mol)**	**Computed vibrational mode**
740	725	700	54	Collective out-of-plane ring deformation
801, 812	785, 796	790	31, 45	Collective CC stretching in the graphene moiety and *t*-Bu deformation, TRIO
831	814	825	24	Collective CC stretching in the graphene moiety and *t*-Bu deformation
892	874	869	46	Pore-SOLO coupled with SOLO at molecule edge
924, 924, 926, 934	906, 906, 907, 915	908	9, 8, 7, 17	Out-of-plane CH bending of the bridge ring at the pore
1277	1251	1253	113	Collective in-plane CH bending and CC stretching at the *t*-Bu insertion
1337	1310	1303	46	Collective CC stretching in the graphene moiety and in-plane CH bending in the pore
1381	1353	1349	79	Collective CC stretching in the graphene moiety and in-plane CH bending
1417	1389	1367	172	Methyl umbrella in *t*-Bu
1534, 1534	1503, 1503	1494	20, 57	CH_2 bending in *t*-Bu and CH bending of the bridge ring at the pore
1624	1592	1567	100	G-like mode (transversal)
1653	1620	1602	492	Stretching of the bridge ring at the pore

Table S4. Vibrational assignment of the Raman spectrum of **pGNR 1**. The first two columns report the unscaled and scaled wavenumbers of the computed normal modes, respectively; the third column reports the peak positions of the experimental features assigned to the wavenumbers in the first two columns; the fourth column reports the computed Raman activities of the vibrational normal modes; finally, the fifth column reports the description of the vibrational normal modes.

DFT wavenumber (cm^{-1})	Scaled DFT wavenumber (cm^{-1})	Experimental peak position (cm^{-1})	Computed Raman activity (Å^4/amu)	Computed vibrational mode
1288, 1290	1262, 1264	1254	5258, 2308	Collective CC stretching near *t*-Bu and CH bending
1330	1303	1307	25080	D mode
1355, 1355	1328, 1328	1333	11260, 81110	CC stretching of the bridge ring at the hole, collective D mode
1376	1348	1350	22170	D mode at the hole periphery
1404, 1417	1376, 1389	1384	8448, 6872	Collective CC stretching in the graphene coupled with *t*-Bu methyl umbrella
1634	1601	1595	21980	G mode (transversal)
1649	1616	1610	40020	G mode at the hole periphery

Table S5. Vibrational assignment of the IR spectrum of **2**. The first two columns report the unscaled and scaled wavenumbers of the computed normal modes, respectively; the third column reports the peak positions of the experimental features assigned to the wavenumbers in the first two columns; the fourth column reports the computed IR intensities of the vibrational normal modes; finally, the fifth column reports the description of the vibrational normal modes.

DFT wavenumber (cm^{-1})	Scaled DFT wavenumber (cm^{-1})	Experimental peak position (cm^{-1})	Computed infrared intensity (km/mol)	Computed vibrational mode
817	800	792	25	DUO
884, 885	866, 867	860	4, 7	Out-of-plane CH bending in the pore
907	888	890	43	SOLO
1401	1372	1373	39	In-plane CH bending in the pore coupled with CH_2 wagging in the alkyl chains
1450	1421	1417	71	Methyl umbrella in *t*-Bu
1539, 1539	1508, 1508	1498	29, 32	CH_2 scissoring in *t*-Bu
1574, 1575	1542, 1543	1533	85, 83	Collective in-plane CH bending coupled with CC stretching near the alkyl chains
1657	1623	1612	171	G-like mode localized at the *t*-Bu insertion points

Table S6. Vibrational assignment of the Raman spectrum of **2**. The first two columns report the unscaled and scaled wavenumbers of the computed normal modes, respectively; the third column reports the peak positions of the experimental features assigned to the wavenumbers in the first two columns; the fourth column reports the computed Raman activities of the vibrational normal modes; finally, the fifth column reports the description of the vibrational normal modes.

DFT wavenumber (cm^{-1})	Scaled DFT wavenumber (cm^{-1})	Experimental peak position (cm^{-1})	Computed Raman activity ($Å^4$/amu)	Computed vibrational mode
1333, 1333	1306	1307	7.90×10^9, 2.14×10^{10}	D modes localized at graphene end
1355, 1355	1327	1335	5.08×10^{10}, 4.25×10^{10}	D modes localized nearby the pore
1631, 1633	1598, 1600	1613	9.24×10^{10}, 1.86×10^9	G mode
1643	1610	1613	6.14×10^9	G mode
1657, 1658, 1660, 1660, 1664, 1665	1623, 1624, 1626, 1626, 1630, 1631	1613	1.20×10^8, 1.07×10^{10}, 4.11×10^8, 5.24×10^9, 1.39×10^8, 1.25×10^8	G mode localized at the alkyl chains and *t*-Bu insertion points

Table S7. Vibrational assignment of the IR spectrum of **pGNR 2**. The first two columns report the unscaled and scaled wavenumbers of the computed normal modes, respectively; the third column reports the peak positions of the experimental features assigned to the wavenumbers in the first two columns; the fourth column reports the computed IR intensities of the vibrational normal modes; finally, the fifth column reports the description of the vibrational normal modes.

DFT wavenumber (cm^{-1})	Scaled DFT wavenumber (cm^{-1})	Experimental peak position (cm^{-1})	Computed infrared intensity (km/mol)	Computed vibrational mode
825, 826, 828, 830, 834	808, 809, 811, 813, 817	Broad feature between 809 and 848 cm^{-1}	5, 5, 2, 5, 14	DUO
855	837	Broad feature between 809 and 848 cm^{-1}	33	SOLO
855, 865	847, 847	829	19, 19	Out-of-plane CH bending in the pore
887	869	865	34	Out-of-plane CH bending in the pore
1402	1373	1375	119	CH_2 wagging in the alkyl chains
1468	1438	1436	45	In-plane CH bending in the pore
1502	1471	1459	36	CH bending in *t*-Bu
1659	1625	1606	66	G-like mode localized at the alkyl chains insertion points

Table S8. Vibrational assignment of the Raman spectrum of **pGNR 2**. The first two columns report the unscaled and scaled wavenumbers of the computed normal modes, respectively; the third column reports the peak positions of the experimental features assigned to the wavenumbers in the first two columns; the fourth column reports the computed Raman activities of the vibrational normal modes; finally, the fifth column reports the description of the vibrational normal modes.

**DFT wavenumber (cm^{-1})**	**Scaled DFT wavenumber (cm^{-1})**	**Experimental peak position (cm^{-1})**	**Computed Raman activity (Å^4/amu)**	**Computed vibrational mode**
1331	1304	1310	256600	D mode
1363	1335	1340	33240	Collective CC stretching in the graphene moiety
1643	1610	1613	28270	Transversal G mode

Table S9. Assignment of selected infrared bands of **3**. The first column reports the wavenumber of the vibrational mode as calculated by DFT, the second column reports the wavenumber multiplied by the scale factor (0.975), the third column reports the position of the experimental peak to which that mode is assigned, the fourth column reports the infrared intensity as calculated by DFT, and the fifth column reports a short description of the vibrational mode. See Figure S11 for graphical representations of the vibrational normal modes.

**Computed wavenumber (cm^{-1})**	**Computed and then scaled frequency (SF = 0.975)**	**Experimental peak position (cm^{-1})**	**Computed infrared intensity (km/mol)**	**Computed vibrational mode**
831	810	798	52	DUO
866	844	866	94	OPLA CH bending at the ortho positions of the rings carrying the alkyl chains coupled with SOLO and DUO
908	885	891	31	SOLO coupled with DUO
1230	1199	1209	13	*t-Bu* CC stretching
1541	1502	1479	33	delocalized CC stretching
1659	1617	1604	236	ring stretching of the rings functionalized with alkyl chains

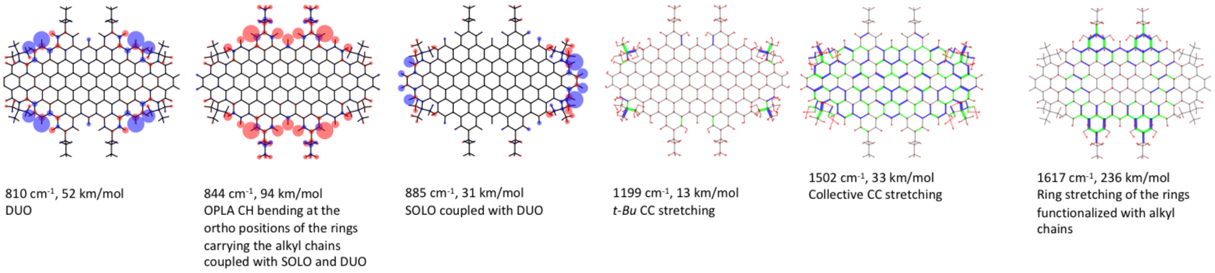

Figure S11. Representation of the vibrational normal modes of **3** mentioned in TableS9. Each cartoon shows the chemical structure of **3**, with blue (green) lines representing contracting (elongating) bonds, red arrows in-plane nuclear displacements, and red and blue circles out-of-plane nuclear displacements with opposite phases. Each cartoon is complemented with the scaled wavenumber, computed infrared intensity, and description of the vibrational normal mode.

Table S10. Assignment of selected Raman bands of **3**. The first column reports the wavenumber of the vibrational mode as calculated by DFT, the second column reports the wavenumber multiplied by the scale factor of 0.975, the third column reports the position of the experimental peak to which that mode is assigned, the fourth column reports the Raman activity computed by DFT, and the fifth column reports a short description of the vibrational mode. See Figure S12 for the graphical representations of the vibrational normal modes. Each cartoon is complemented with the scaled wavenumber, the computed Raman activity, and the description of the vibrational normal mode.

**Computed wavenumber (cm^{-1})**	**Computed and then scaled frequency (SF = 0.975)**	**Experimental peak position (cm^{-1})**	**Computed Raman activity ($Å^4$/amu)**	**Computed vibrational mode**
1260	1229	1238	1935	localized D-like mode
1336	1303	1311	148595	D mode
1626	1585	1605	18823	longitudinal G mode
1644	1603		9958	transversal G mode

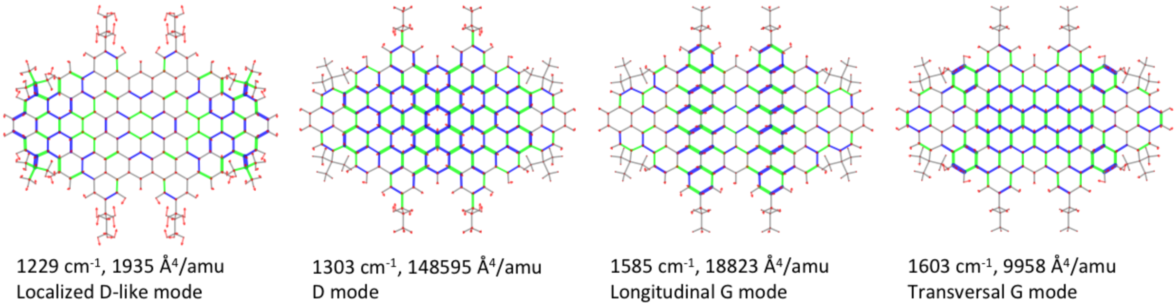

Figure S12. Representation of the vibrational normal modes of **3** mentioned in TableS10. Each cartoon shows the chemical structure of **3**, with blue (green) lines representing contracting (elongating) bonds and red arrows in-plane nuclear displacements. Each cartoon is complemented with scaled wavenumber, computed Raman activity, and description of the vibrational normal mode.

Table S11. Assignment of selected infrared bands of **npGNR**. The first column reports the wavenumber of the vibrational mode as calculated by DFT, the second column reports the wavenumber multiplied by the scale factor of 0.975, the third column reports the position of the experimental peak to which that mode is assigned, the fourth column reports the infrared intensity as calculated by DFT, and the fifth column reports a short description of the vibrational mode. See Figure S13 for graphical representations of the vibrational normal modes.

**Computed wavenumber (cm^{-1})**	**Computed and then scaled frequency (SF = 0.975)**	**Experimental peak position (cm^{-1})**	**Computed infrared intensity (km/mol)**	**Computed vibrational mode**
764	764	756	1	OPLA of the bay region
784	784	790	12	DUO
797	797	808	14	DUO
1165	1165	1168	26	*t-Bu* CC stretching
1306	1306	1303	81	collective CC stretching

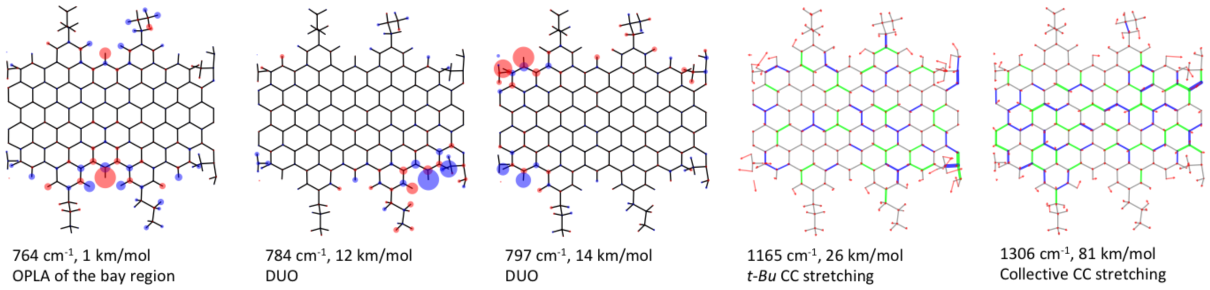

Figure S13. Representation of the vibrational normal modes of **npGNR** mentioned in TableS11. Each cartoon shows the chemical structure of the repeat unit of **npGNR**, with blue (green) lines representing contracting (elongating) bonds, red arrows in-plane nuclear displacements, and red and blue circles out-of-plane nuclear displacements with opposite phases. Each cartoon is complemented with scaled wavenumber, computed infrared intensity, and description of the vibrational normal mode.

Table S12. Assignment of selected Raman bands of **npGNR**. The first column reports the wavenumber of the vibrational mode as calculated by DFT, the second column reports the wavenumber multiplied by the scale factor of 0.975, the third column reports the position of the experimental peak to which that mode is assigned, the fourth column reports the Raman activity as calculated by DFT, and the fifth column reports a short description of the vibrational mode. See Figure S14 for graphical representations of the vibrational normal modes.

**Computed wavenumber (cm^{-1})**	**Computed and then scaled frequency (SF = 0.975)**	**Experimental peak position (cm^{-1})**	**Computed Raman activity ($Å^4$/amu)**	**Computed vibrational mode**
1246	1246	1249	153856	D-like mode
1303	1303	1312	1432195	D-like mode
1328	1328		1230689	D-like mode
1531	1531	1603	67655	longitudinal G mode
1533	1533		473661	transversal G mode

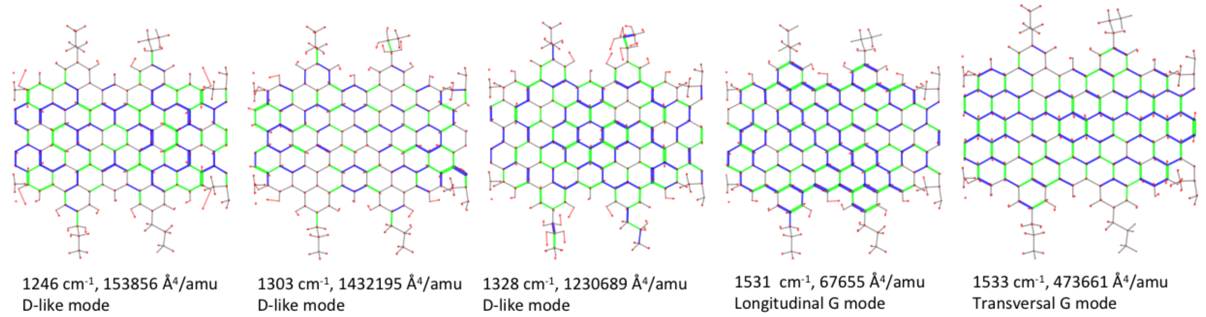

Figure S14. Representation of the vibrational normal modes of **npGNR** mentioned in TableS12. Each cartoon shows the chemical structure of the repeat unit of **npGNR**, with blue (green) lines representing contracting (elongating) bonds and red arrows in-plane nuclear displacements. Each cartoon is complemented with scaled wavenumber, computed Raman activity, and description of the vibrational normal mode.

Details on the resonant Raman spectra of 2. Figure S15 shows the resonance Raman spectra computed for **2** using a range of excitation wavelength (380-460 nm). The simulated spectra are compared with the observed spectrum recorded with a 405 nm laser. In the main text, we choose the spectrum computed at 400 nm as the best match. Due to the simplifications involved in the model of **2**, and the lack of solid-state intermolecular effects, this optimal wavelength slightly differs from the experimental one (405 nm) and the agreement can be considered acceptable.

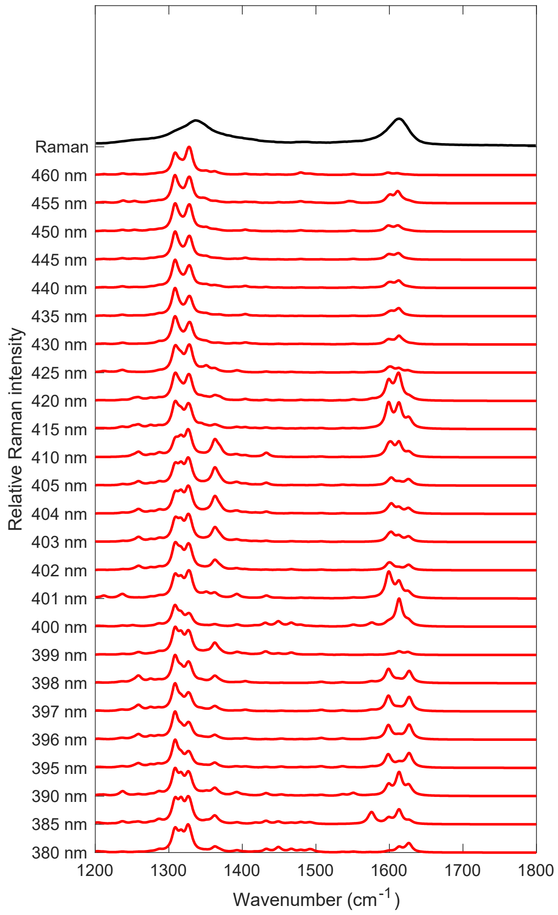

Figure S15. Normalized experimental (black line) and computed (red lines) Raman spectra of **2**. Red spectra are computed at several excitation wavelengths, indicated on the left.

6. Solid-state NMR analysis

Solid-state NMR experiments were carried out on a 300 MHz Bruker Ascend spectrometer equipped with a 2.5 mm double-resonance magic angle spinning (MAS) probe at 15 kHz sample spinning rate. ^{1}H NMR spectra were recorded at 300.13 MHz resonance frequency using direct single-pulse excitation (3.6 µs pulse length) with a recycle delay of 3 s between subsequent scans. ^{13}C cross polarization (CP) MAS NMR and directly excited (DE) MAS NMR spectra were recorded at a 75.47 MHz resonance frequency using ramped CP with ^{1}H decoupling (SPINAL-64), 4 ms CP contact time, and 3 s recycle delay. The spectra were referenced relative to TMS using adamantane as secondary reference. Background spectra were subtracted from the ^{1}H NMR spectra, leaving only the signals due to the samples. Expected intensity-weighted average chemical shifts $\bar{\delta}$ were calculated from predicted spectra for the theoretical structures using the ACD/Labs NMR Predictor program.

Results and Discussion

^{1}H MAS NMR

The solid-state ^{1}H MAS NMR spectra of the polymer precursors **P1**, **P2** and **P3**, as well as their corresponding synthesized GNRs, **pGNR 1**, **pGNR 2** and **npGNR**, are shown in Figure S16. The signals span from the aliphatic region (centered between ca. 0.2 and 1.6 ppm; see Table S13) to the aromatic region (~6.3 –7.6 ppm). The relatively broad and thus unresolved aliphatic hydrogen signals representing the different CH_2 and CH_3 groups were observed for all six samples at chemical shifts approximately 1 ppm lower than the predicted average of 1.2 ppm and 1.3 ppm. This tendency is likely caused by ring-current effects. Aliphatic hydrogens positioned above or below aromatic π-systems which are frequently present in the samples are more shielded by the magnetic field produced by the π-electron cloud, resulting in lower chemical shift values. The minor, very narrow signals observed at ca. 0.10 ppm in samples **P2**, **P3**, and **pGNR 2** are attributed to residual organic solvents and silicone grease.[10] After the planarization, the aliphatic signal of **P3**, originally found at 0.4 ppm splits into two relatively narrow signals (each less than 300 Hz wide) at -0.8 ppm and 1.6 ppm (**npGNR**; Figure S16c). This splitting into two separate, sharper peaks may indicate a more ordered structural state for the aliphatic hydrogens. The aliphatic peak is then resolved into two components, possibly corresponding to the secondary and tertiary hydrogens of the 3,7-dimethyloctyl groups (based on the predicted spectra of a single repeating unit of the material, generated using the ACD/Labs NMR Predictor program).

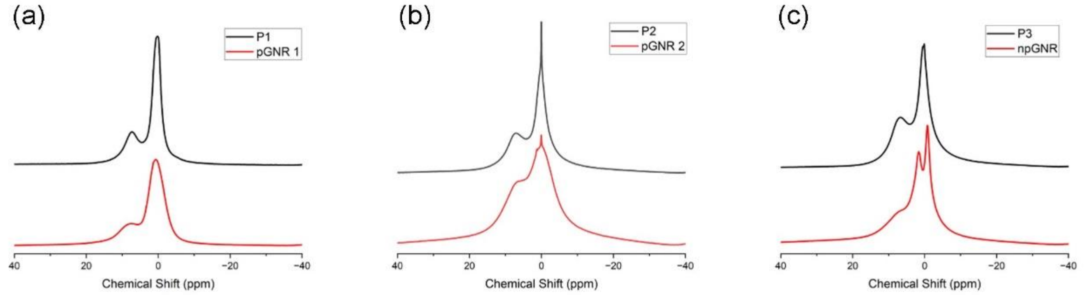

Figure S16. ^{1}H MAS NMR spectra of (a) **P1** and **pGNR 1**, (b) **P2** and **pGNR 2**, and (c) **P3** and **npGNR**.

Table S13. Summary of ^{1}H NMR chemical shifts.

Sample	type	δ (ppm)	Calculated δ (ppm)
**P1**	aliphatic	0.4	1.3
	aromatic	7.3	8.0
**P2**	aliphatic	0.2	1.2
	aromatic	7.0	8.8
**P3**	aliphatic	0.4	1.2
	aromatic	6.8	7.5
**pGNR 1**	aliphatic	0.6	1.3
	aromatic	7.6	7.7
**pGNR 2**	aliphatic	0.4	1.2
	aromatic	6.3	N/A
**npGNR**	aliphatic	-0.8<br>1.6	1.2
	aromatic	6.6	N/A

^{13}C CP and DE MAS NMR

Figure S17 shows the CP spectra (blue) and directly excited (DE) spectra (orange) for the precursors **P1**, **P2** and **P3** (Figures S17a, S17b and S17c, respectively), as well as for the GNRs **pGNR 1, pGNR 2** and **npGNR** (Figures S17d, S17e and S17f, respectively). Aliphatic signals corresponding to the isobutyl and 3,7-dimethyloctyl groups were observed between 14 and 45 ppm. The relatively high relative intensity of these signals in the CP spectra suggests that the aliphatic carbons in the precursors **P1**, **P2** and **P3**, but also in the GNRs, are rather immobile.

Aromatic signals in the CP spectra appear between 110 and 155 ppm. This chemical shift range is consistent with the predicted spectra generated by the ACD/Labs NMR Predictor program. In the DE spectra, an additional signal was observed between ca. 90 and 125 ppm (marked in light orange). This signal is attributed to non-protonated aromatic carbons located within the inner benzene rings of the π-system, which are absent in the CP spectra due to their distance from polarization-transferring hydrogens. Generally, non-protonated carbons within the inner π-system tend to contribute to lower-frequency signals, while other (peripheral) non-protonated aromatic carbons (ca. 133–180 ppm; marked in purple) tend to appear farther downfield. Protonated aromatic carbons (110-140 ppm; marked in green) tend to contribute to higher-frequency signals. Furthermore, the two relatively distinct peaks in the aromatic region of the precursors **P2** and **P3** spectra, centered at 128 ppm and 142 ppm, suggest a higher degree of order/crystallinity in these materials. In general, increased substitution reduces electron density and shielding (through the reduction of the conjugation in the π-system and prevention of efficient delocalization of electrons), resulting in higher chemical shifts. Therefore, any additional peaks around 150 ppm are attributed to the aromatic carbons bonded to the tert-butyl groups (distinctively observed in the **P1** and **pGNR 1** spectra, where number of tert-butyl groups in the structure is larger). Lastly, the ratios between aliphatic and aromatic carbons were calculated for the expected structures and found to agree with the measured intensities obtained from the ^{13}C DE MAS NMR spectra. This observation further corroborates the suggested structures.

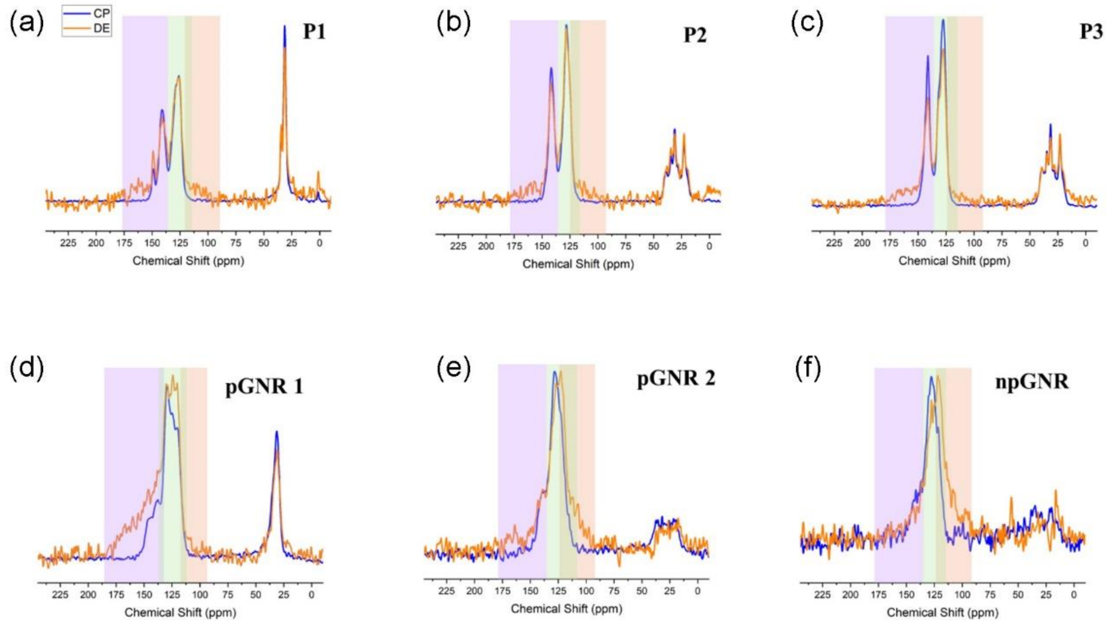

Figure S17. ^{13}C CP MAS NMR (blue spectra) and directly excited ^{13}C DE MAS NMR (orange) spectra of the synthesized precursors **P1** (a), **P2** (b) and **P3** (c), and their corresponding GNRs, **pGNR 1** (d), **pGNR 2** (e) and **npGNR** (f).

7. Theoretical calculations

The structural optimization and electronic property calculations of all nanographene systems were carried out using density functional theory (DFT) as implemented in the Vienna ab initio Simulation Package (VASP 5.4.4).[11] The projector augmented-wave (PAW)[12] method was employed in conjunction with the Perdew–Burke–Ernzerhof (PBE)[13] exchange-correlation functional. To account for dispersion interactions, Grimme's D3 dispersion correction[14] was applied.

Given the well-known underestimation of band gaps by the PBE functional, the hybrid Heyd–Scuseria–Ernzerhof (HSE06)[15] functional was further employed to recalculate the band structures and more accurately determine the band gaps. A Monkhorst-Pack k-point mesh of 8 × 1 × 1 was used for the optimization of atomic coordinates and lattice parameters, while a finer mesh of 10 × 1 × 1 was used to obtain converged charge densities.

For the model compounds, geometry optimizations were performed at the PBE0/def2-TZVP level of theory using the Gaussian16 package.[7] Time-dependent DFT (TD-DFT) calculations were carried out with 20 excited states (nstates = 20) to simulate the UV-vis absorption spectra.

Table S14. HOMO-LUMO gaps of model compounds calculated at PBE0/def2tzvp level.

model compounds	HOMO-LUMO gap (eV)
**1**	3.16
**2**	2.85
**3**	2.31

Table S15. Electronic band gaps for graphene nanoribbons calculated at HSE06 level and their effective masses.

	Band gap (eV)	Effective mass (m_e)		m^*
		hole	electron	
**pGNR 1**	2.39	7.43	1.09	0.951
**pGNR 2**	2.01	0.33	0.35	0.169
**npGNR**	1.56	0.22	0.24	0.115

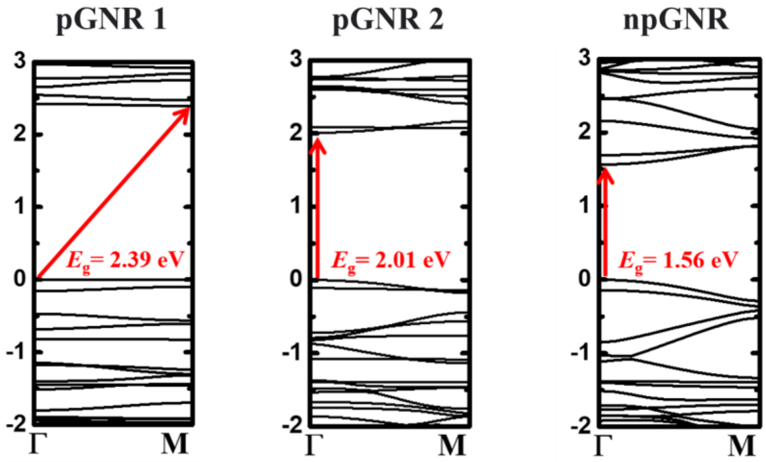

Figure S18. Electronic band structures of graphene nanoribbons calculated at HSE06 level. The band gaps are noted in the figure.

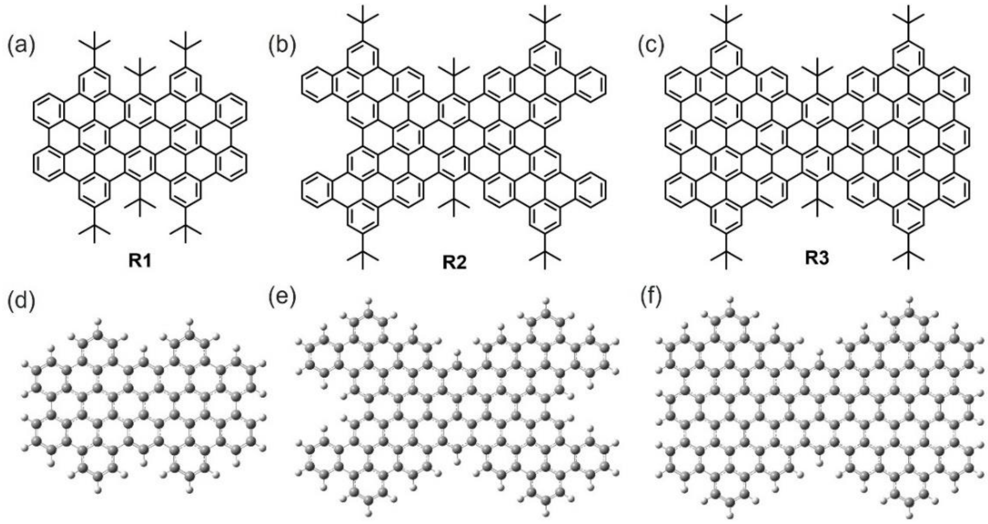

Figure S19. (a-c) Chemical structures of suggested molecular references **R1**, **R2**, and **R3**. (d-f) Optimized geometries of **R1**, **R2**, and **R3**, with *t*Bu groups removed to simplify the calculations.

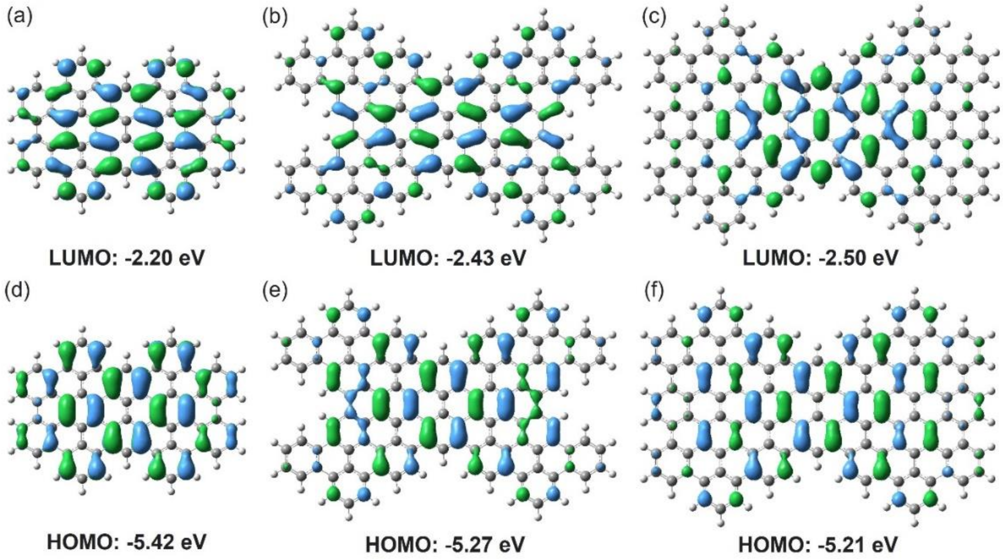

Figure S20. Molecular orbitals of molecular references **R1**, **R2**, and **R3**, respectively.

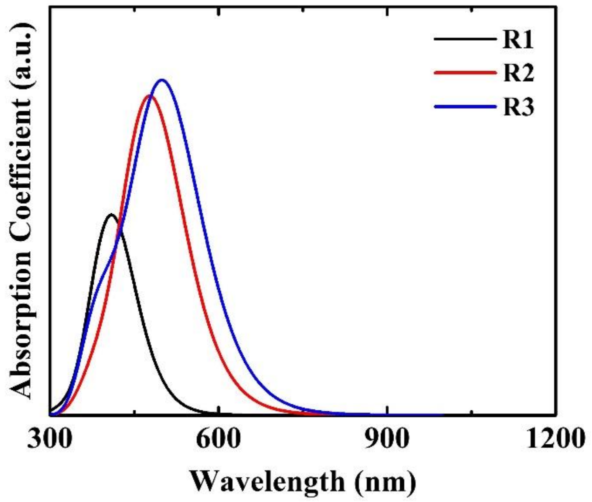

Figure S21. Calculated UV-vis absorption spectra of molecular references **R1**, **R2**, and **R3**, respectively.

8. STM experiments at the solid/liquid interface

Scanning tunneling microscopy (STM) experiments were carried-out using a Molecular Imaging stm (Agilent Technologies) at room temperature operating in constant-current mode. Experimental STM parameters, I_{set} and U_{bias}, are indicated in each figure and correspond to the tunnelling current and the applied sample potential bias, respectively. Tips were cut mechanically from a Pt/Ir wire (80/20, diameter 0.25 mm, Advent Research Materials). High-resolution STM images were calibrated (when indicated) using the underlying graphite lattice and processed using Scanning Probe Image Processor (SPIP, Image Metrology) software while the molecular models were fabricated using HyperChem™ software (version 8.0.1). STM imaging at the solid liquid interface started immediately after drop-casting the corresponding solution on the solid substrate. The substrate was always highly oriented pyrolytic graphite (HOPG) (grade ZYB, Momentive Performance Material Quartz Inc., Strongsville, OH, USA), which was freshly cleaved using Scotch tape prior every experiment. Solutions were prepared using as-received 1,2,4-trichlorobenzene (TCB, Merck) as solvent. Prior to drop-casting, the compounds were firstly dissolved (1mg/mL), sonicated and then diluted 10 times (v/v) and sonicated further for 15 min.

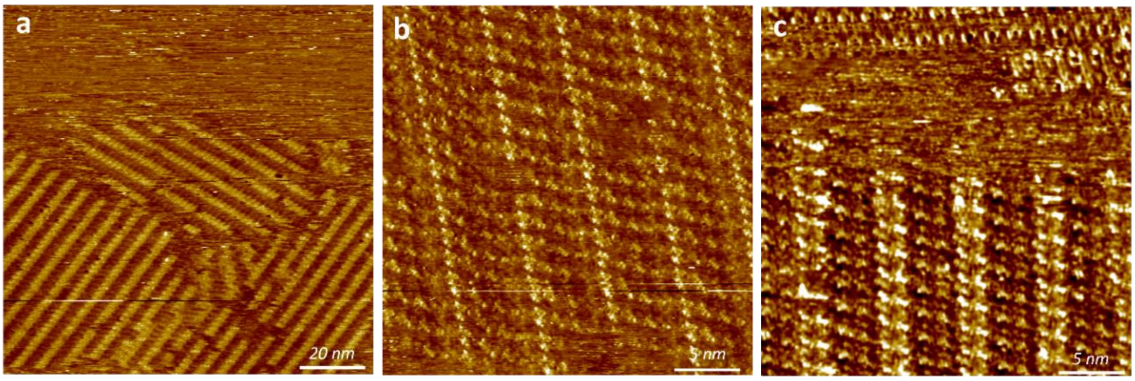

Figure S22. (a) Large-scale and (b-c) small-scale STM images of **1** at the **TCB/HOPG** interface. I_{set} = 0.18 nA and U_{bias} =-0.50V.

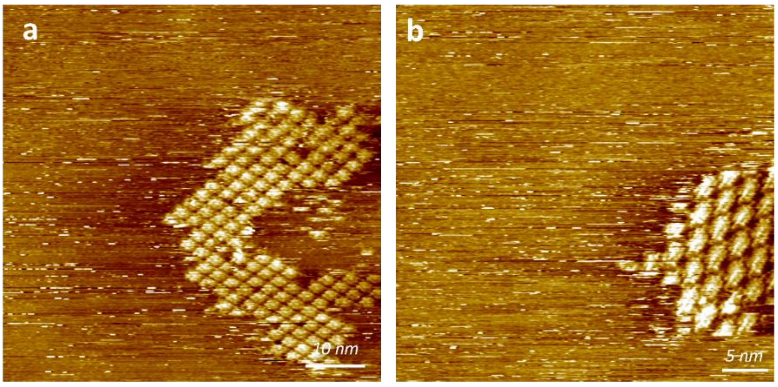

Figure S23. (a-b) Typical STM images of **2** at the **TCB/HOPG** interface featuring small islands of **2**. I_{set} = 0.12 nA and U_{bias} =-0.75V.

9. Terahertz spectroscopic study of pGNR 1

Optical-pump THz-probe spectroscopy is employed to investigate the charge carrier dynamics of **pGNR 1**. The time-resolved photoconductivity data following 3.1 eV excitation (Figure S24) reveals that the real part of the photoconductivity for **pGNR 1** remains essentially zero, while the imaginary part shows a finite negative response. This is a hallmark of bound excitons, not free carriers, as the latter would yield a finite positive real component.[16] Since the optical bandgap of **pGNR 1** is ~2.4 eV, our results indicate that excitations at 3.1 eV do not produce free charges, meaning the exciton binding energy E_b must be much larger than the 700 meV of excess energy. This is not a surprising result, as it is in line with the large exciton effects (on the order of several 100s of meV) predicted by theory,[17,18] and confirmed by experimental studies[16,19].

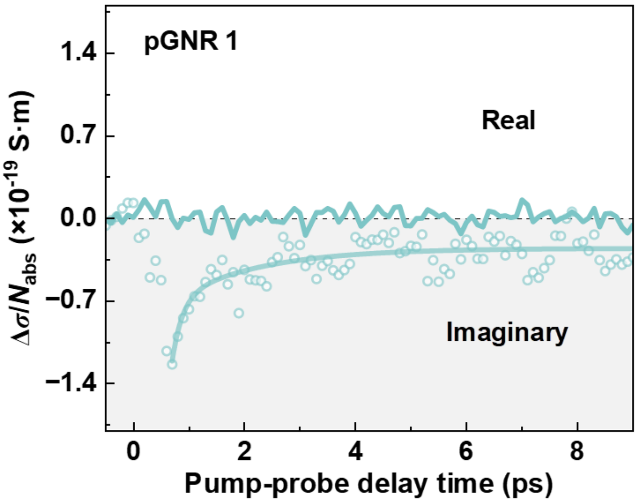

Figure S24. Time-resolved complex terahertz photoconductivity normalized to the absorbed photon density ($\Delta\sigma/N_{abs}$) of **pGNR 1** following photoexcitation at 3.1 eV. The solid line in the imaginary part of photoconductivity is a guide to the eye to illustrate the overall trend.

10. NMR spectra

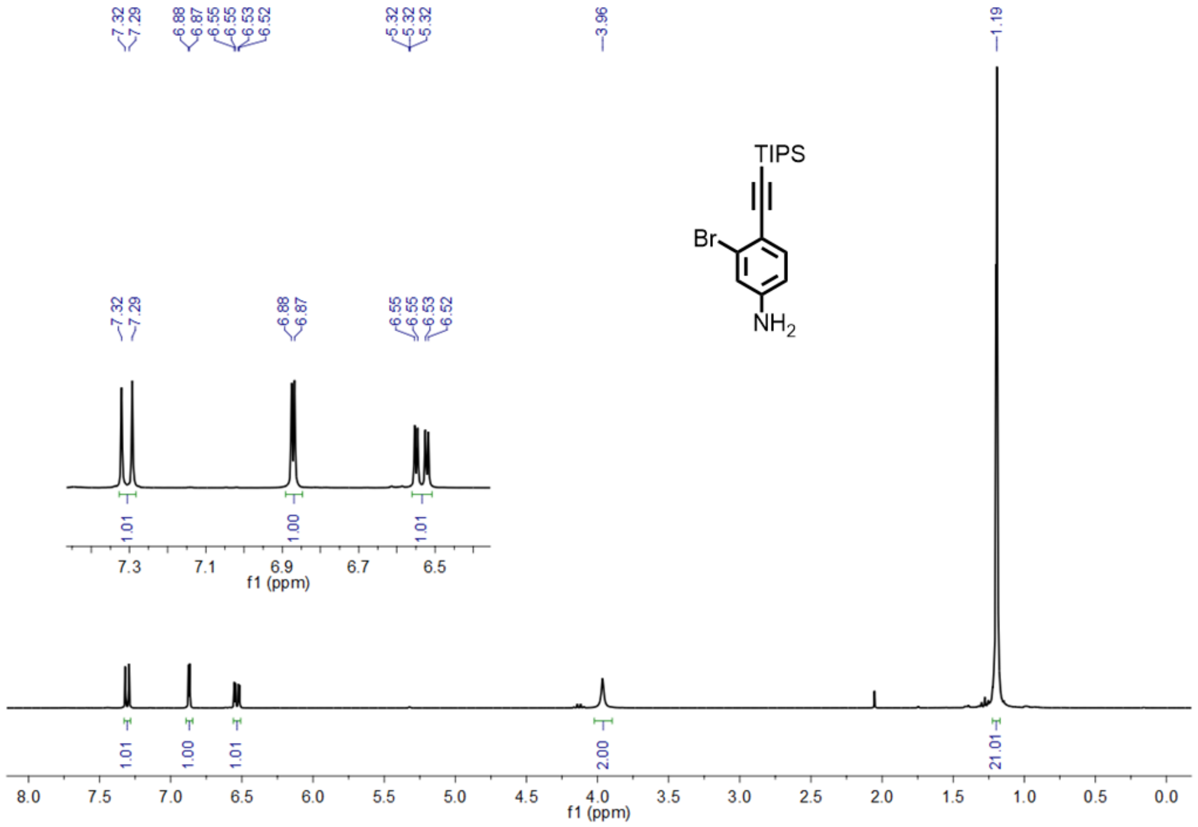

Figure S25. ^{1}H NMR spectrum (300 MHz) of **4** in CD_2Cl_2 at room temperature.

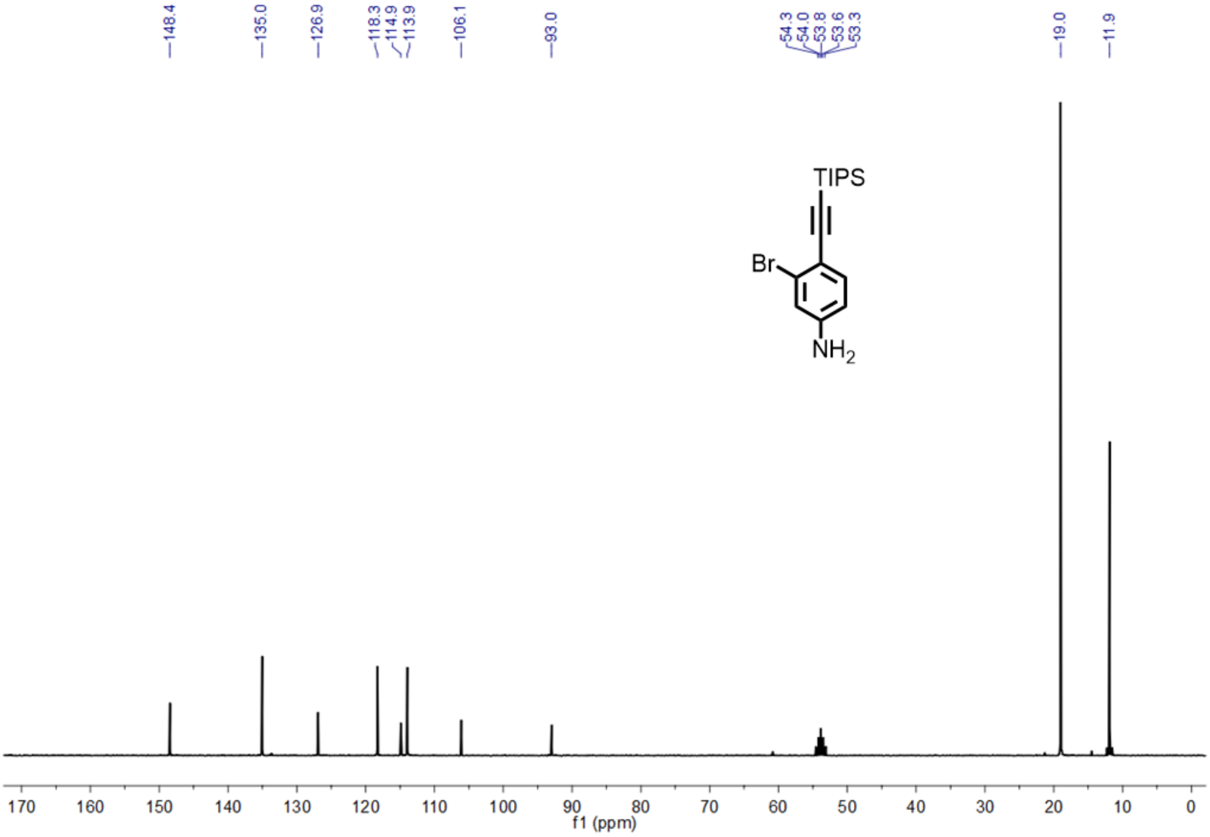

Figure S26. ^{13}C NMR spectrum (75 MHz) of **4** in CD_2Cl_2 at room temperature.

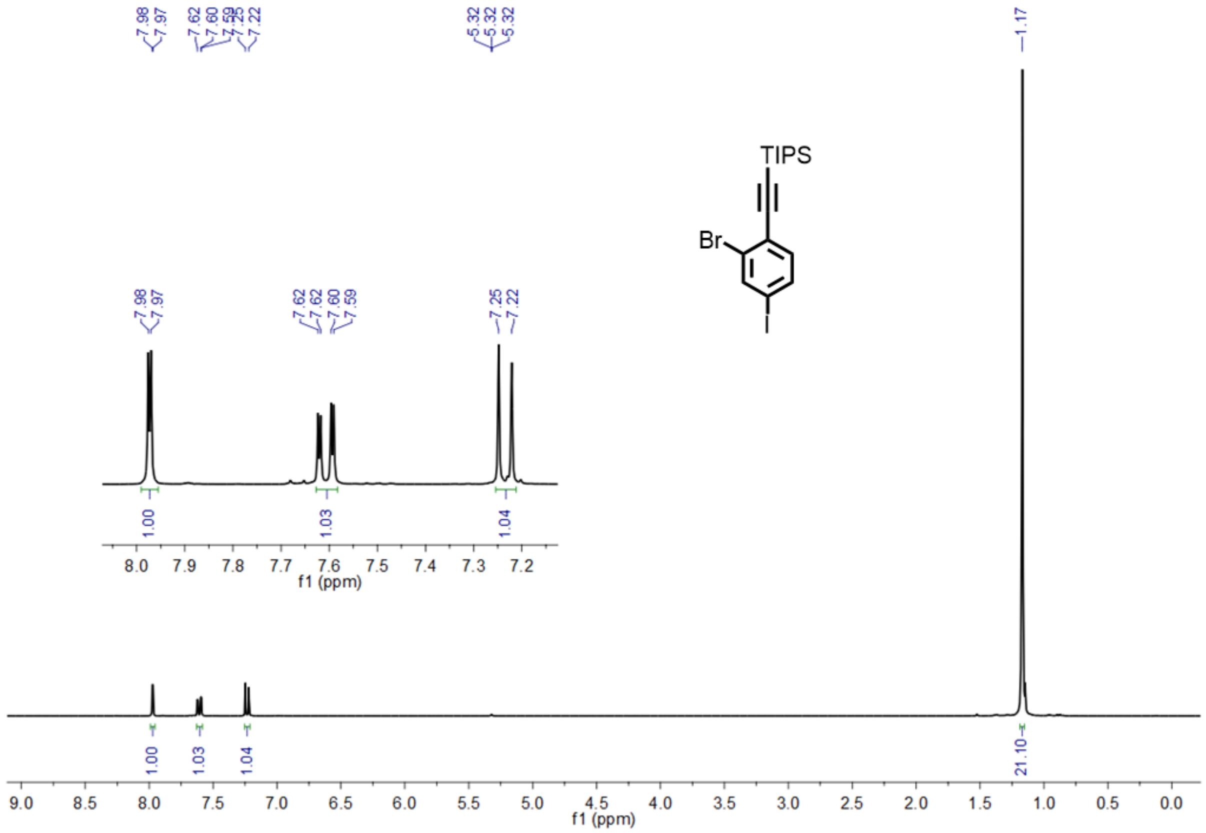

Figure S27. ^{1}H NMR spectrum (300 MHz) of **5** in CD_2Cl_2 at room temperature.

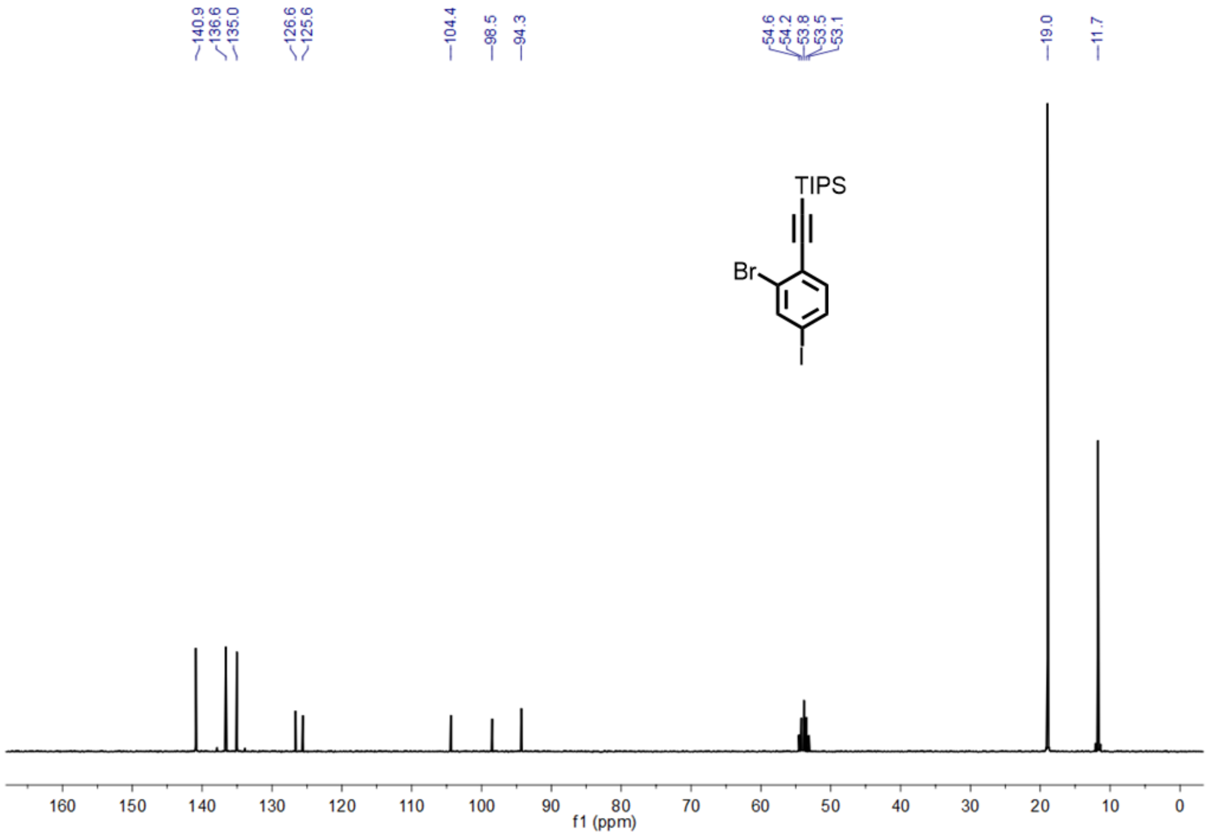

Figure S28. ^{13}C NMR spectrum (75 MHz) of **5** in CD_2Cl_2 at room temperature.

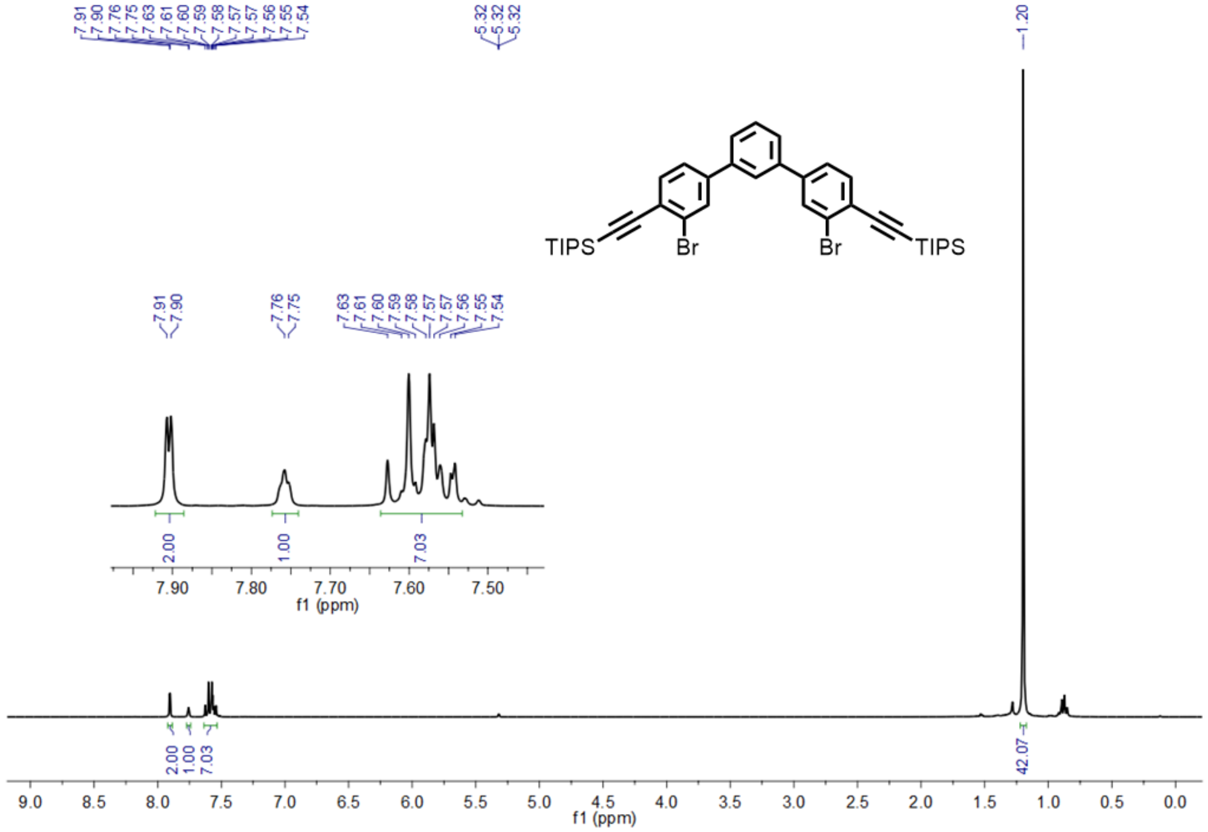

Figure S29. ^{1}H NMR spectrum (300 MHz) of **6** in CD_2Cl_2 at room temperature.

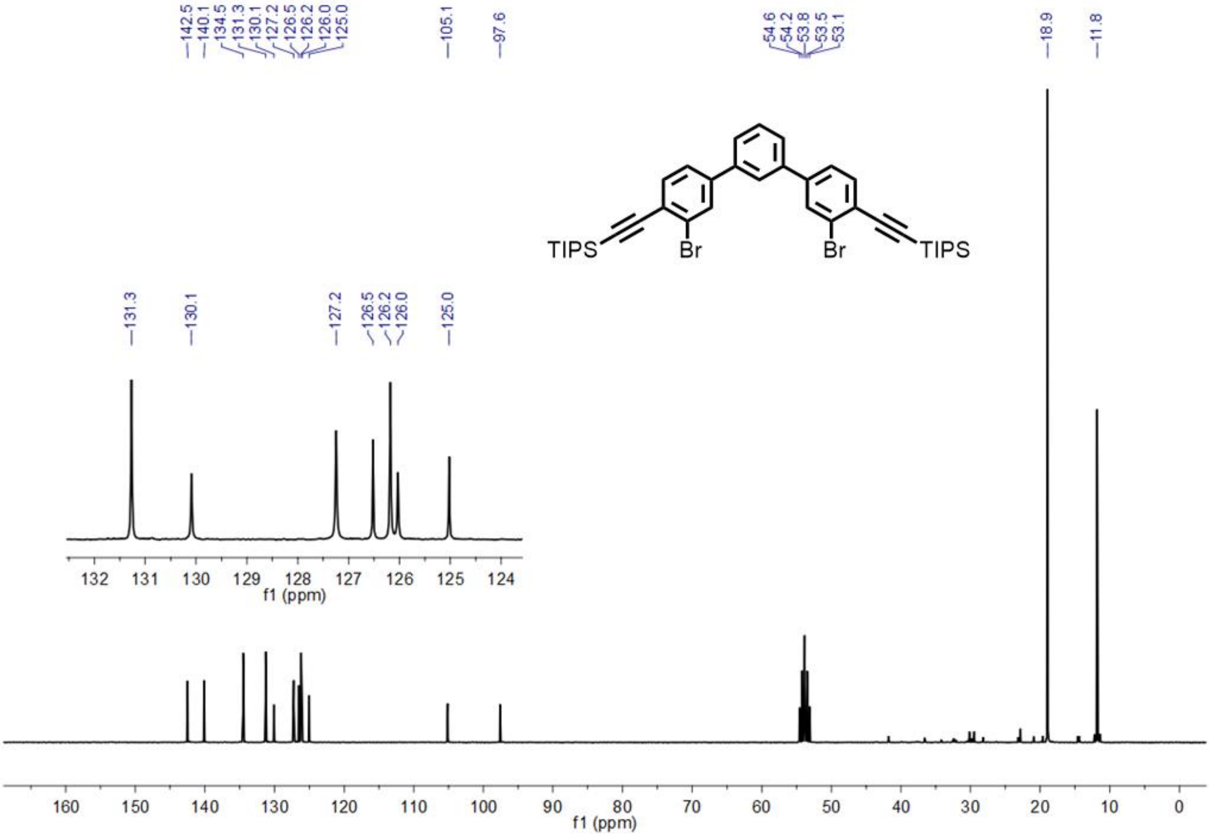

Figure S30. ^{13}C NMR spectrum (75 MHz) of **6** in CD_2Cl_2 at room temperature.

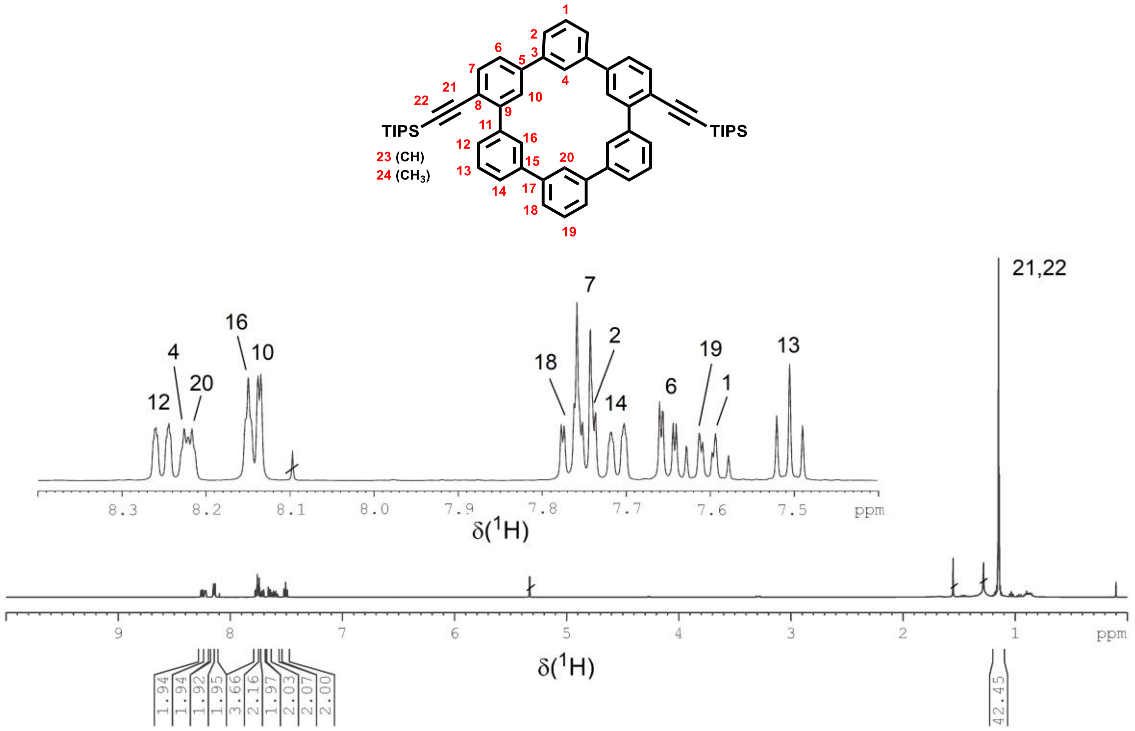

Figure S31. ^{1}H NMR spectrum (500 MHz) of **9-1** in CD_2Cl_2 at 30°C.

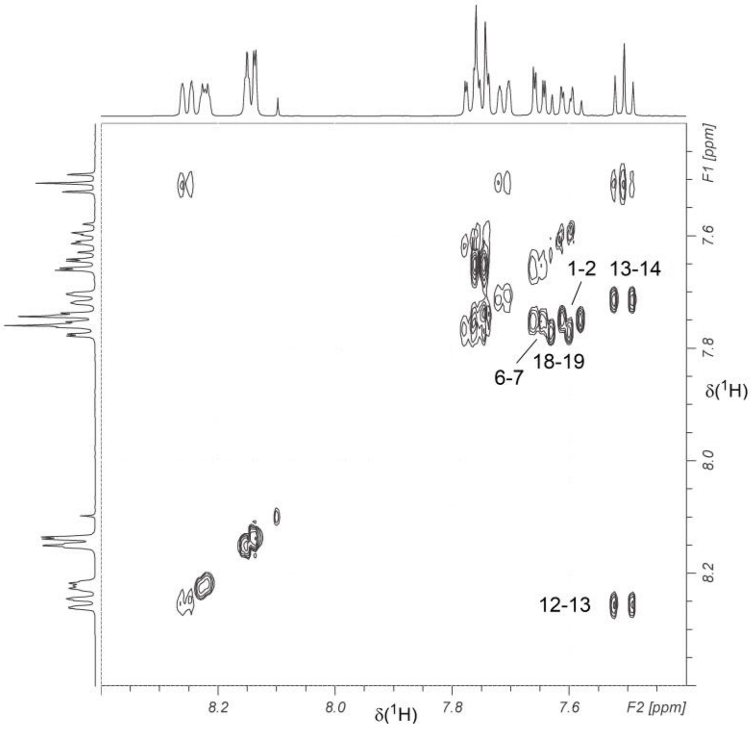

Figure S32. COSY spectrum of **9-1** in CD_2Cl_2 at 30°C showing $^3J_{HH}$ correlations.

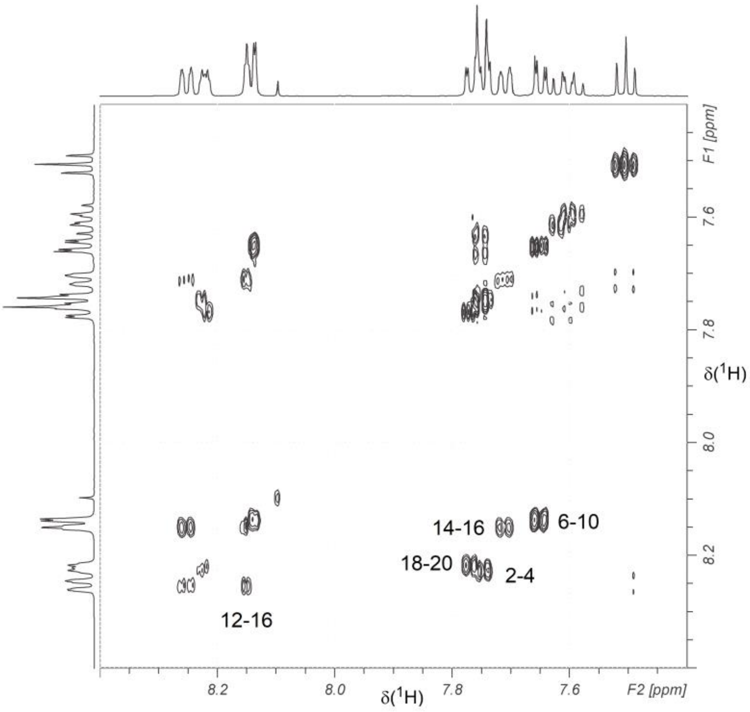

Figure S33. Long-range COSY spectrum of **9-1** in CD_2Cl_2 at 30°C showing $^4J_{HH}$ correlations.

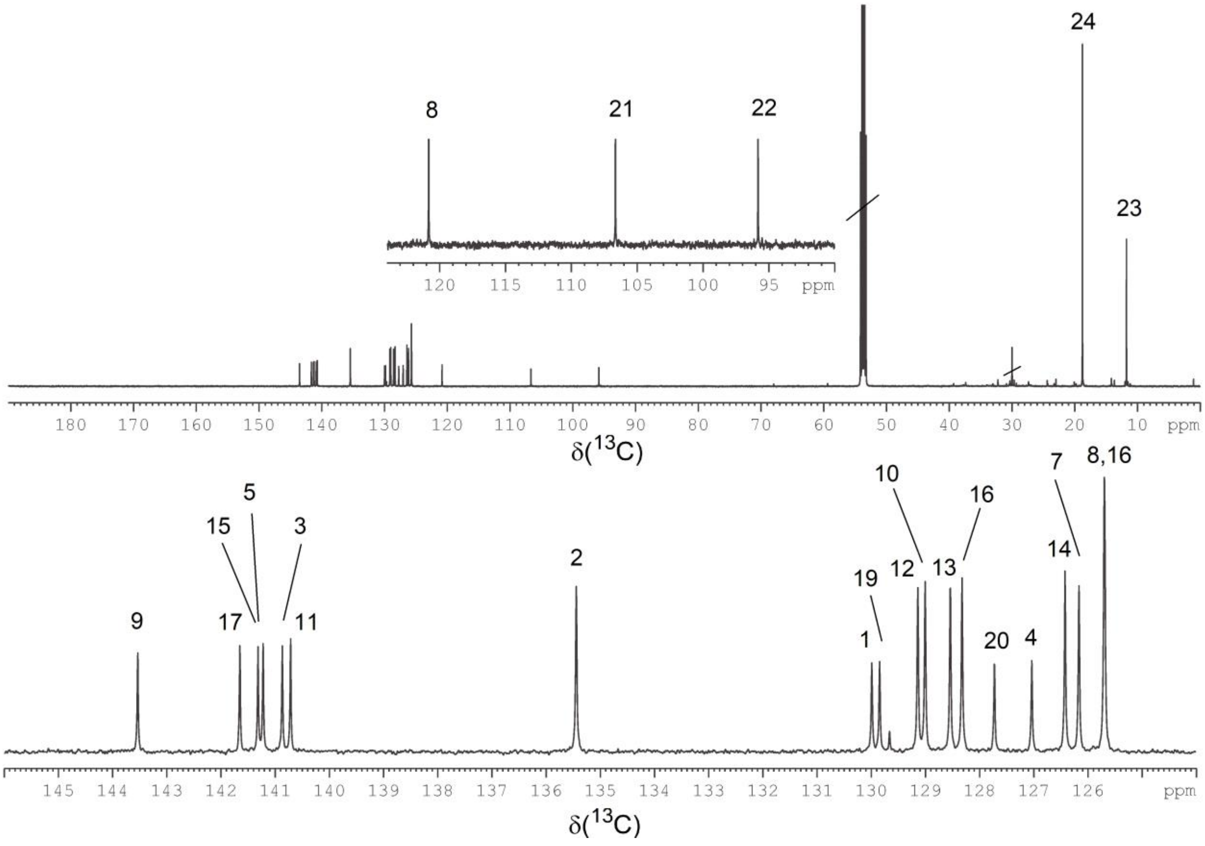

Figure S34. ^{13}C NMR spectrum (125 MHz) of **9-1** in CD_2Cl_2 at 30°C.

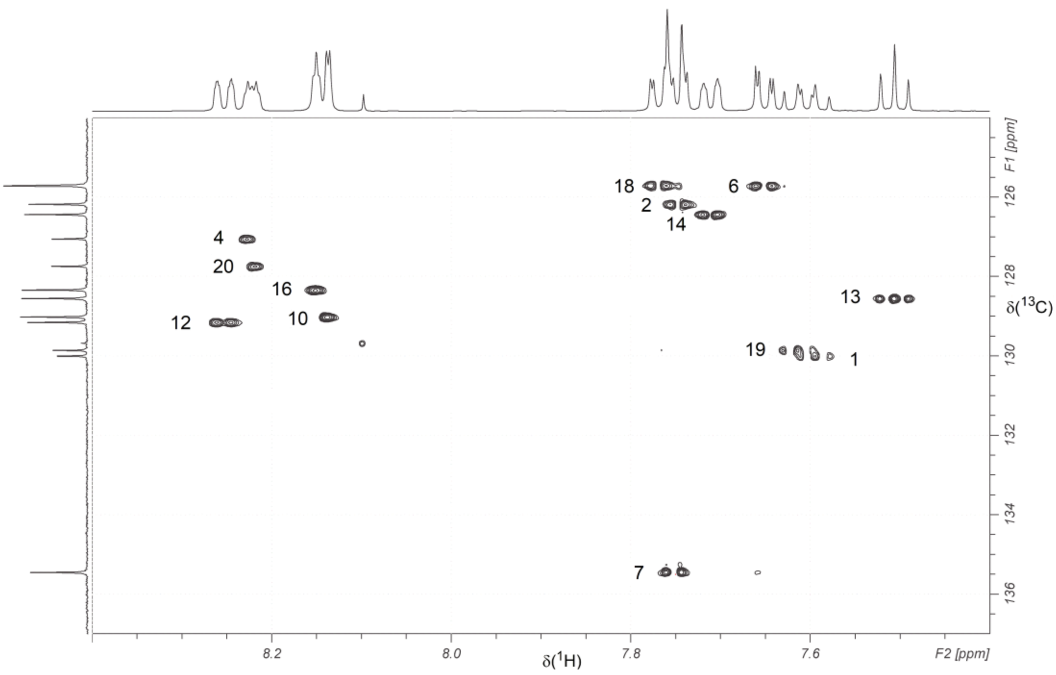

Figure S35. HSQC spectrum (region of aromatic CH) of **9-1** in CD_2Cl_2 at 30°C. The F1 trace is the DEPT135 spectrum.

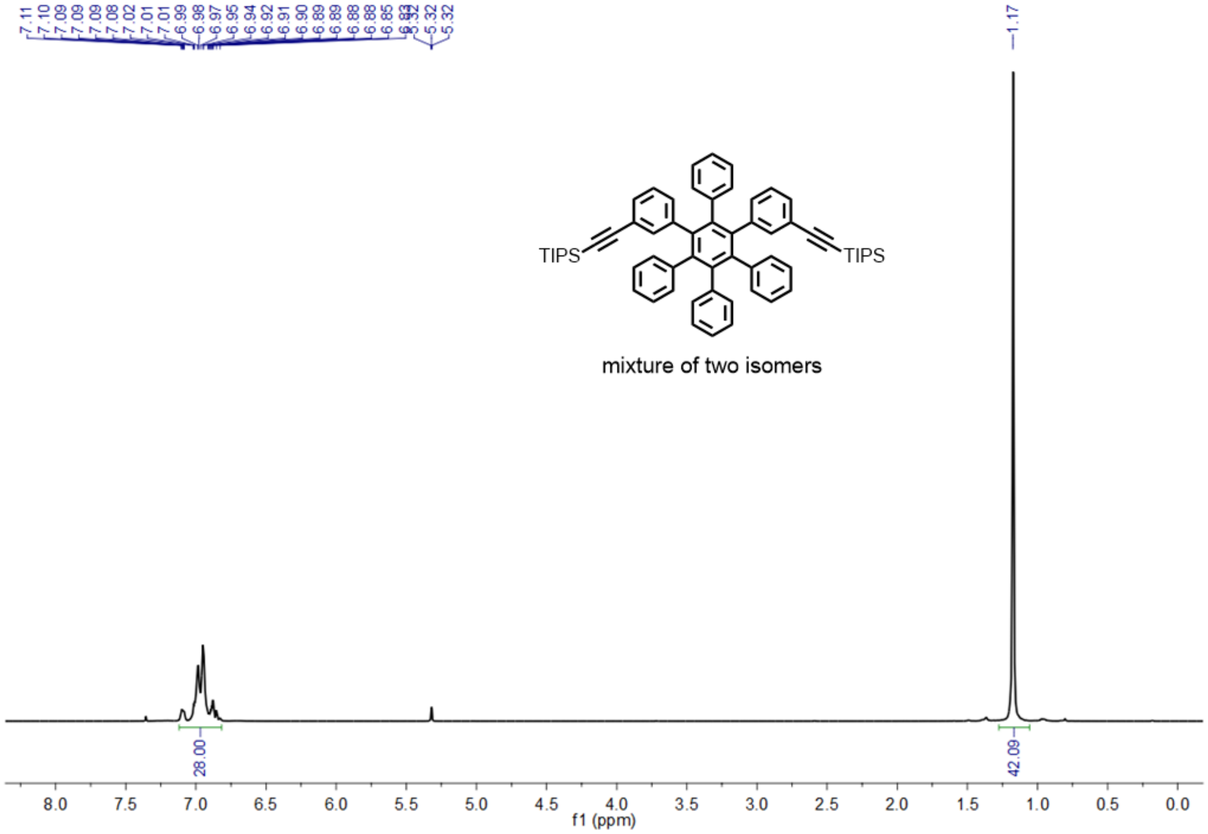

Figure S36. ^{1}H NMR spectrum (300 MHz) of **10-1** in CD_2Cl_2 at room temperature.

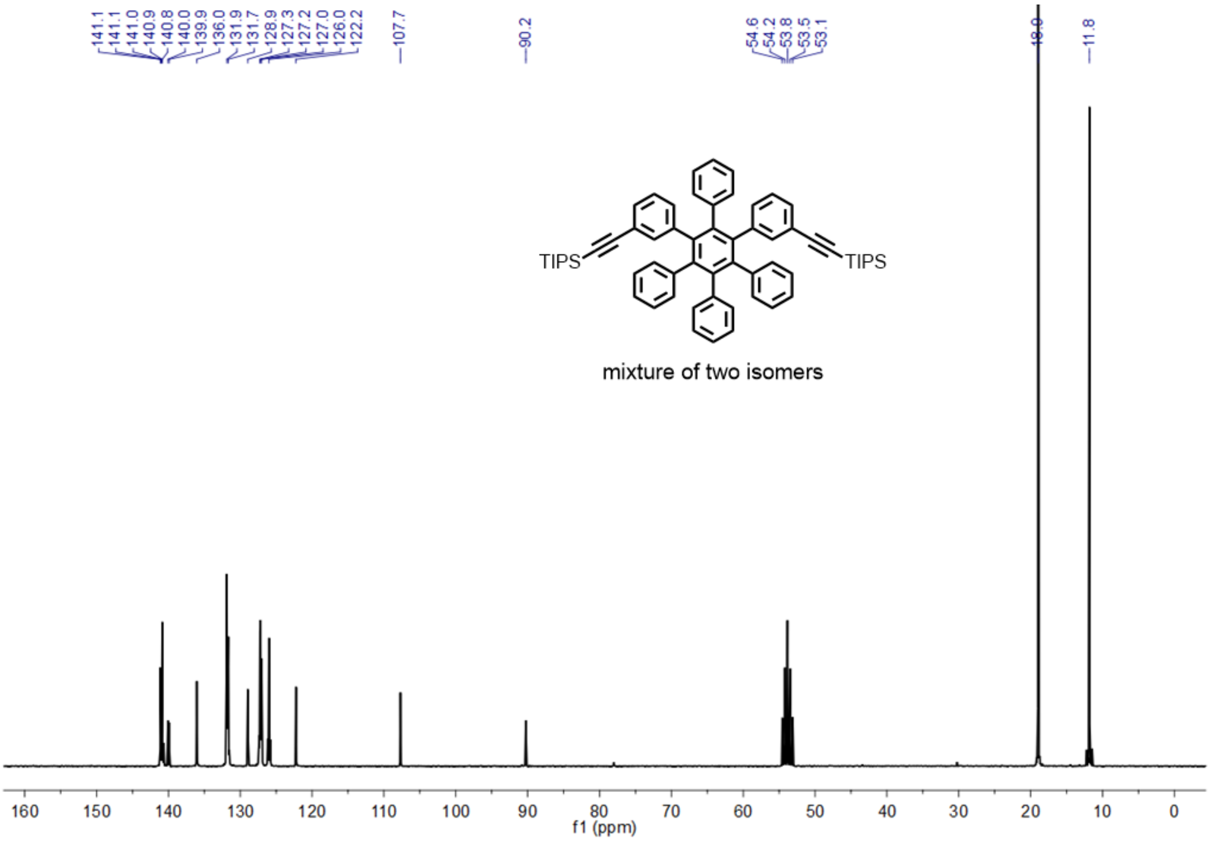

Figure S37. ^{13}C NMR spectrum (75 MHz) of **10-1** in CD_2Cl_2 at room temperature.

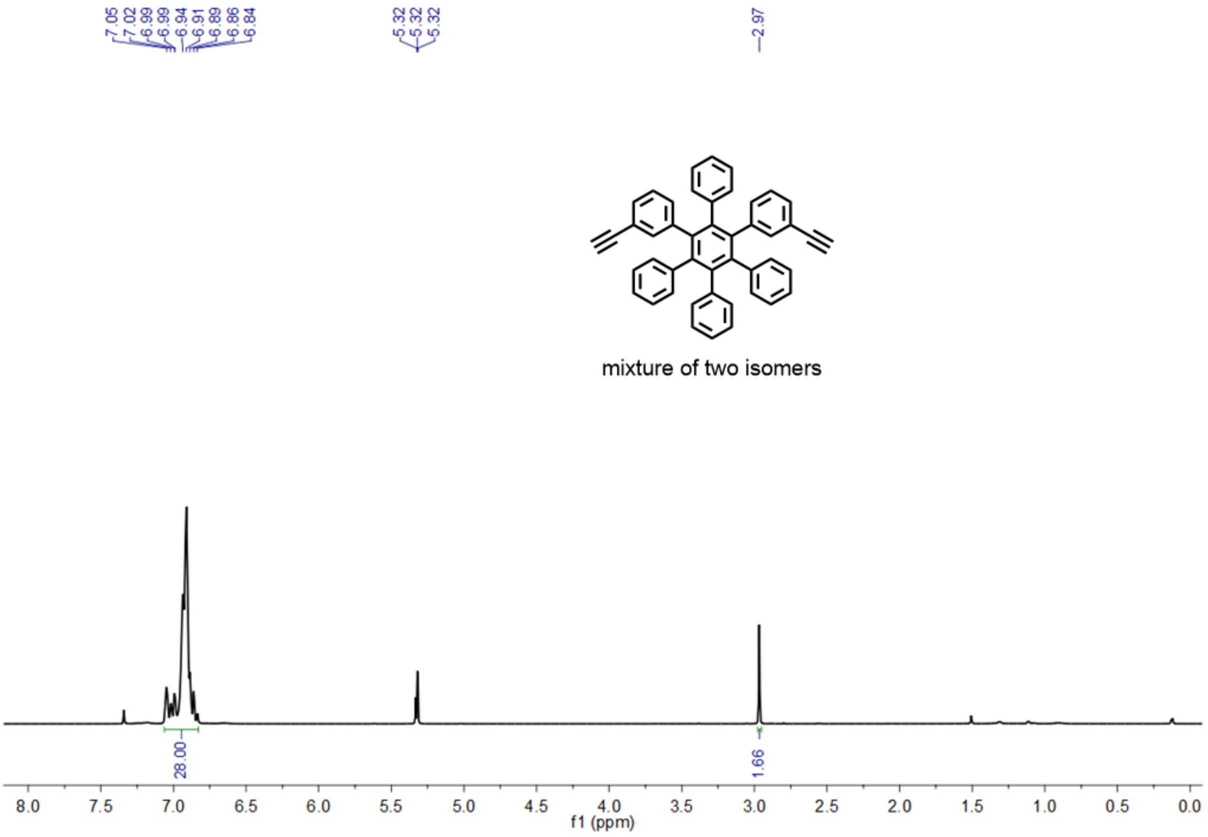

Figure S38. ^{1}H NMR spectrum (300 MHz) of **10** in CD_2Cl_2 at room temperature.

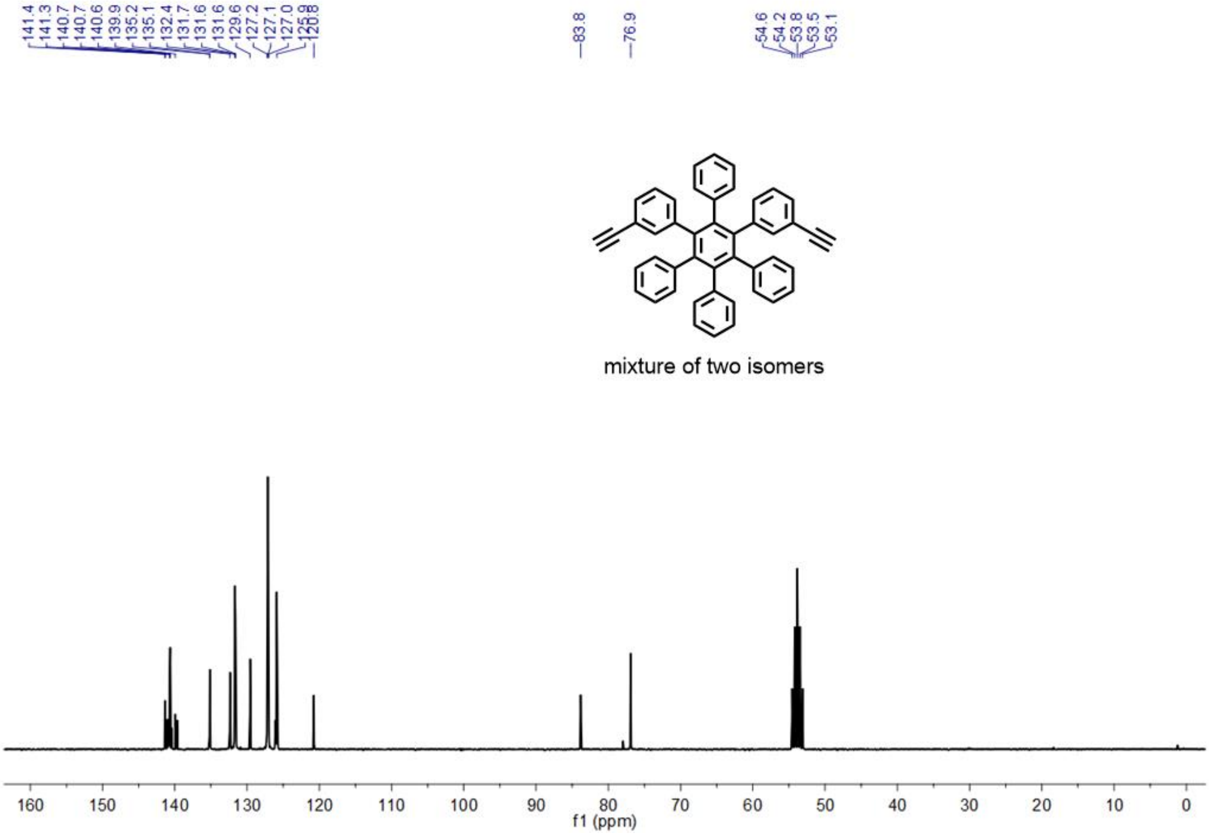

Figure S39. ^{13}C NMR spectrum (75 MHz) of **10** in CD_2Cl_2 at room temperature.

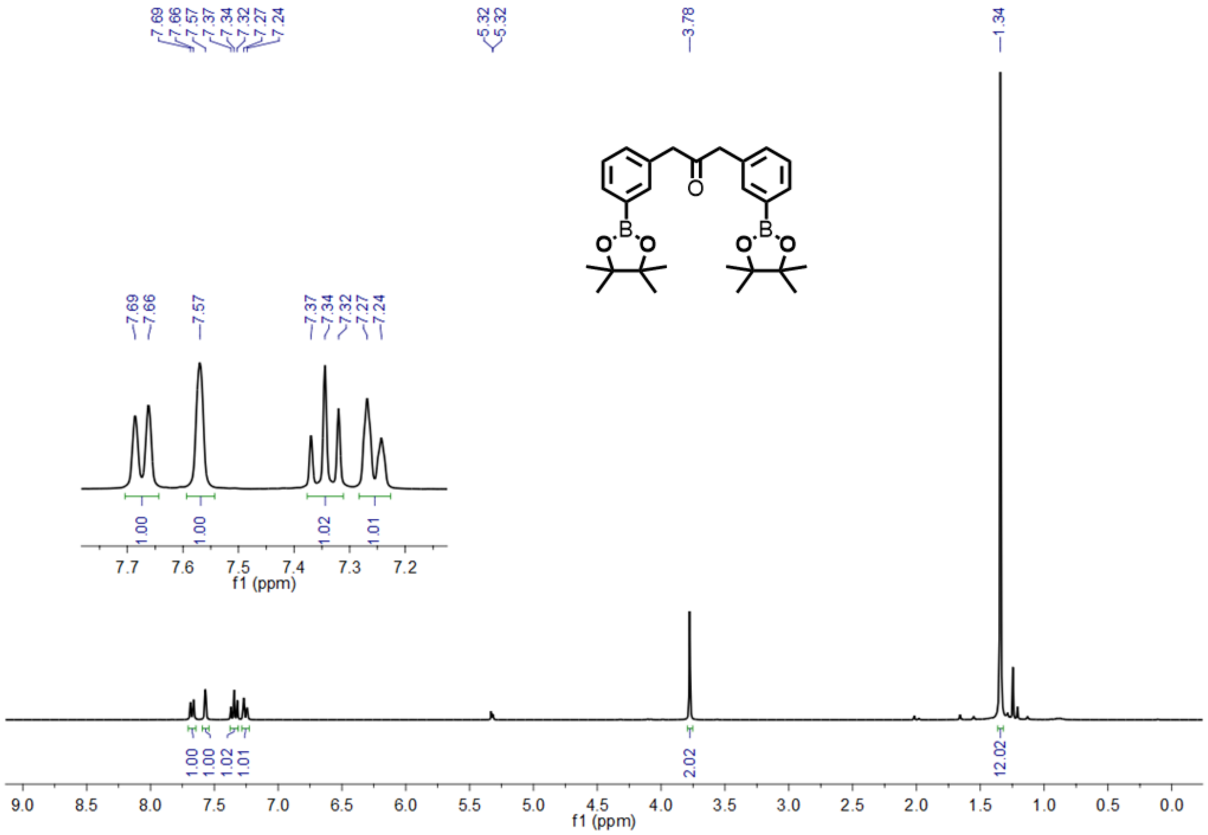

Figure S40. ^{1}H NMR spectrum (300 MHz) of **21** in CD_2Cl_2 at room temperature.

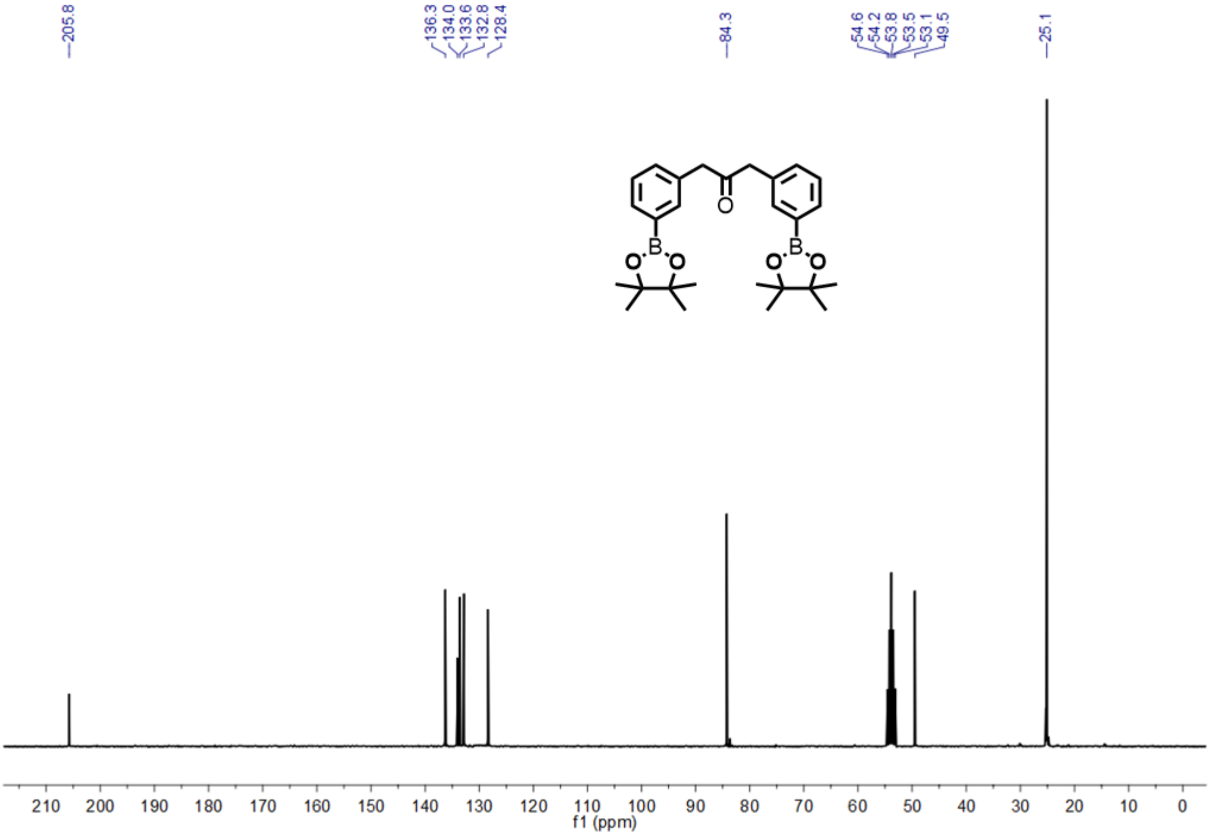

Figure S41. ^{13}C NMR spectrum (75 MHz) of **21** in CD_2Cl_2 at room temperature.

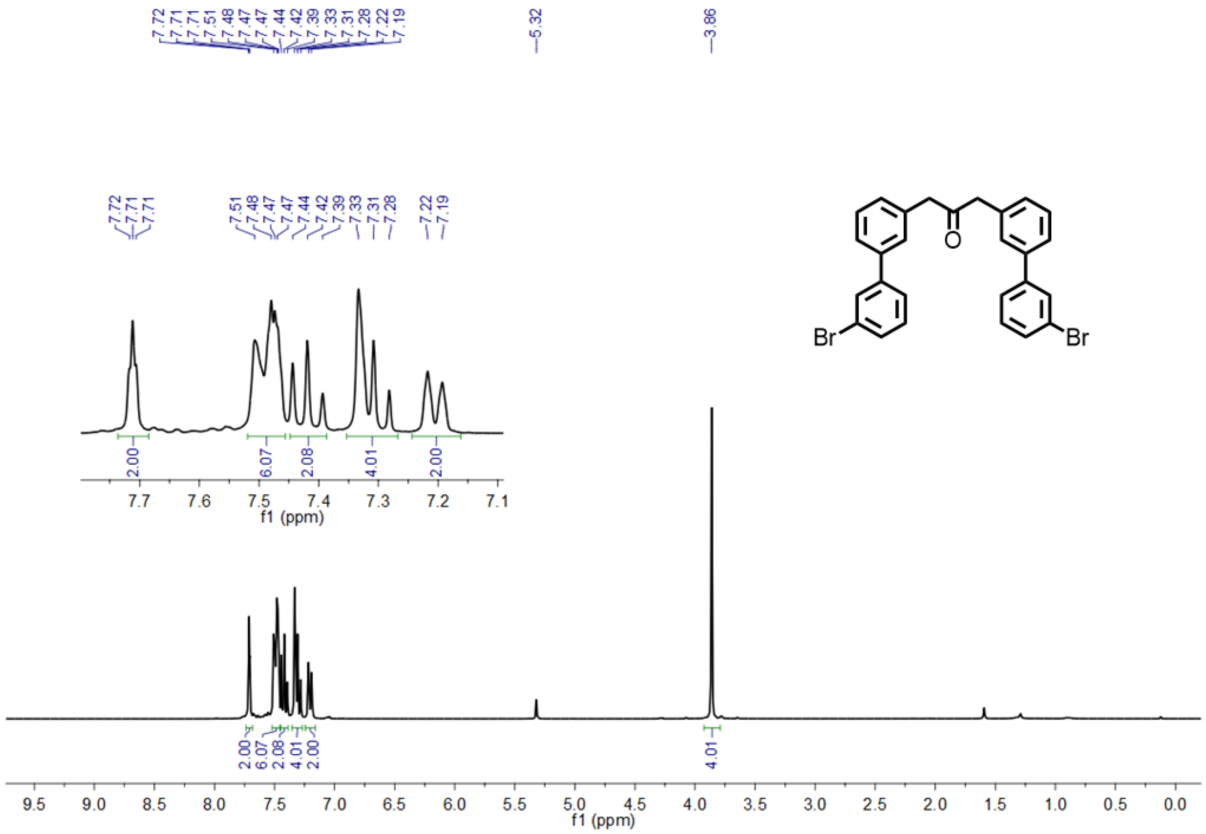

Figure S42. ^{1}H NMR spectrum (300 MHz) of **22** in CD_2Cl_2 at room temperature.

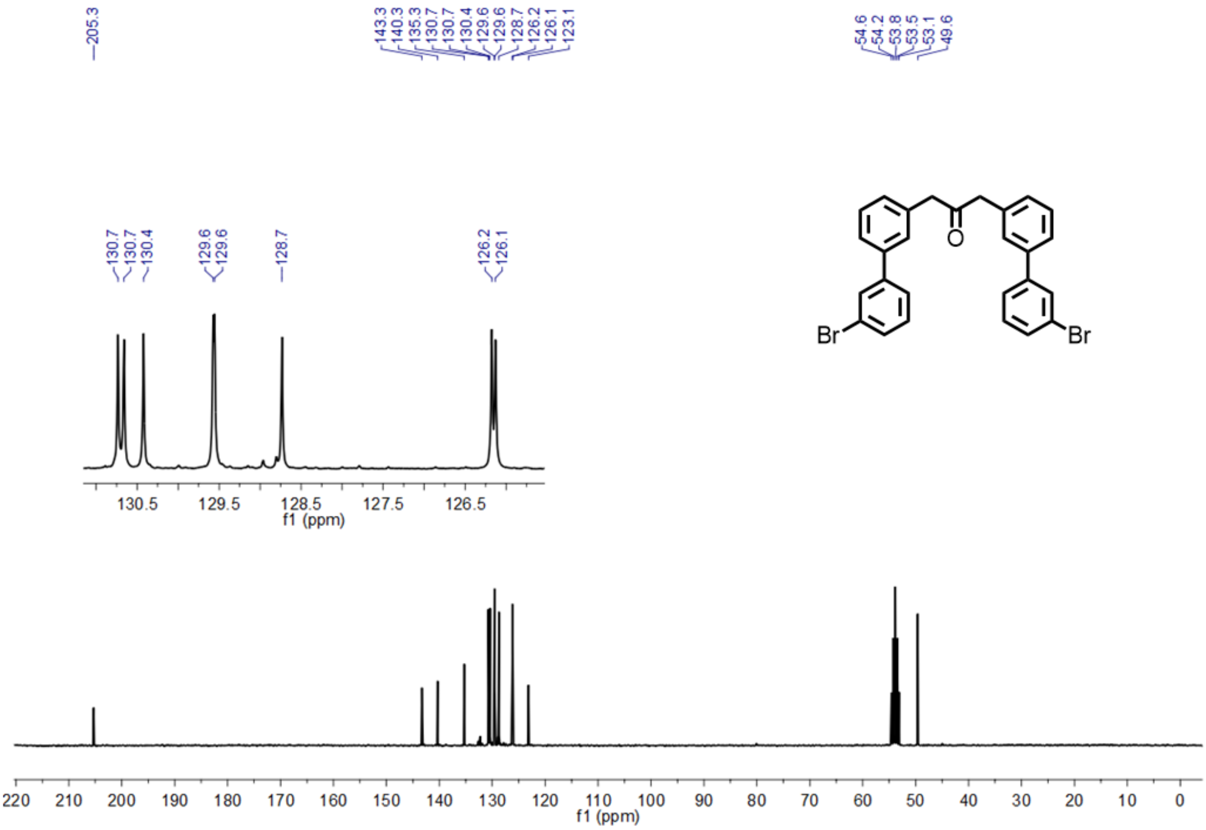

Figure S43. ^{13}C NMR spectrum (75 MHz) of **22** in CD_2Cl_2 at room temperature.

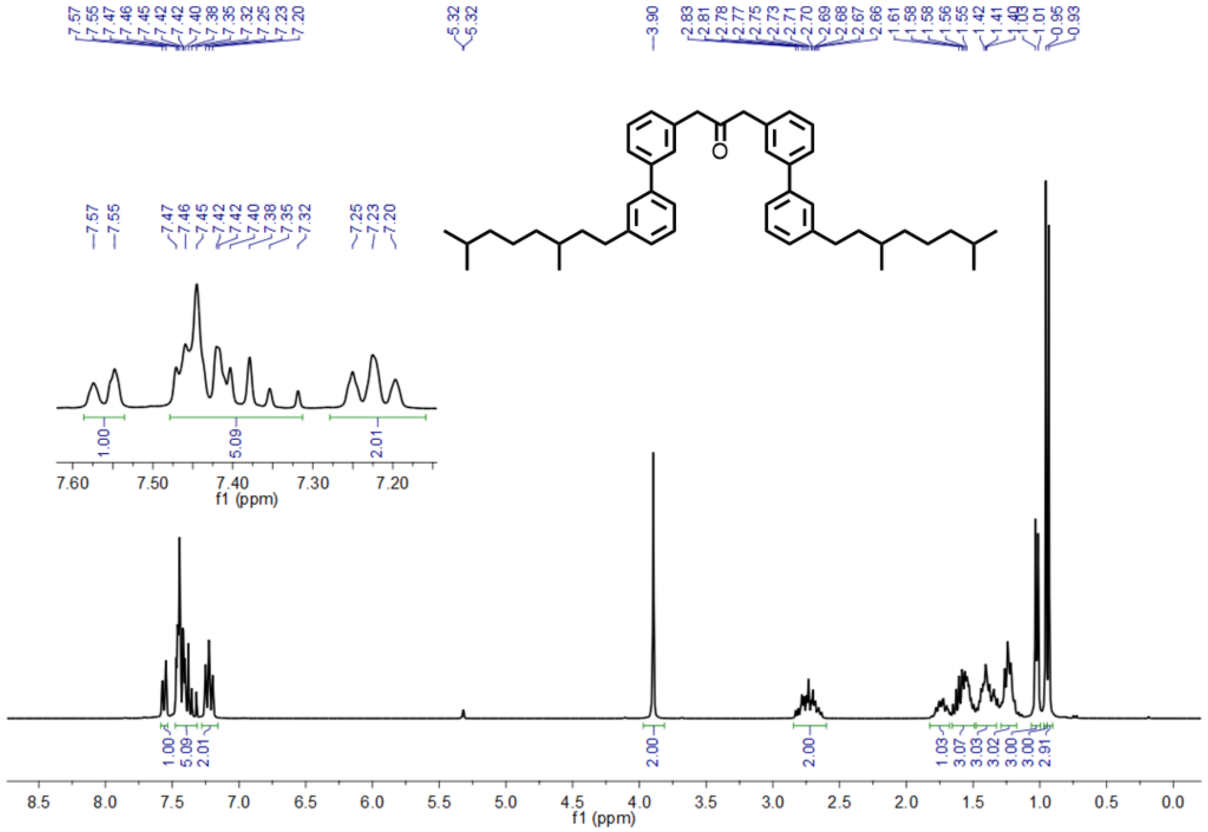

Figure S44. ^{1}H NMR spectrum (300 MHz) of **23** in CD_2Cl_2 at room temperature.

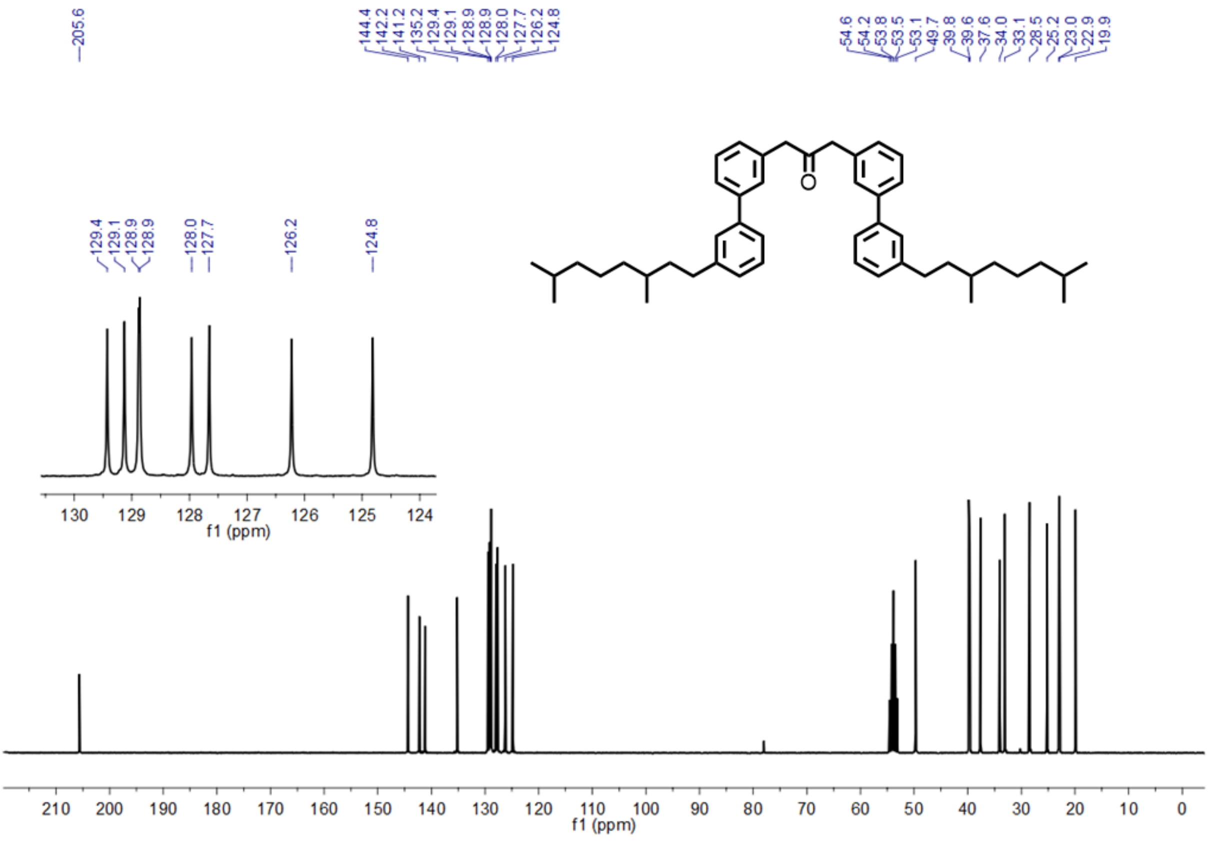

Figure S45. ^{13}C NMR spectrum (75 MHz) of **23** in CD_2Cl_2 at room temperature.

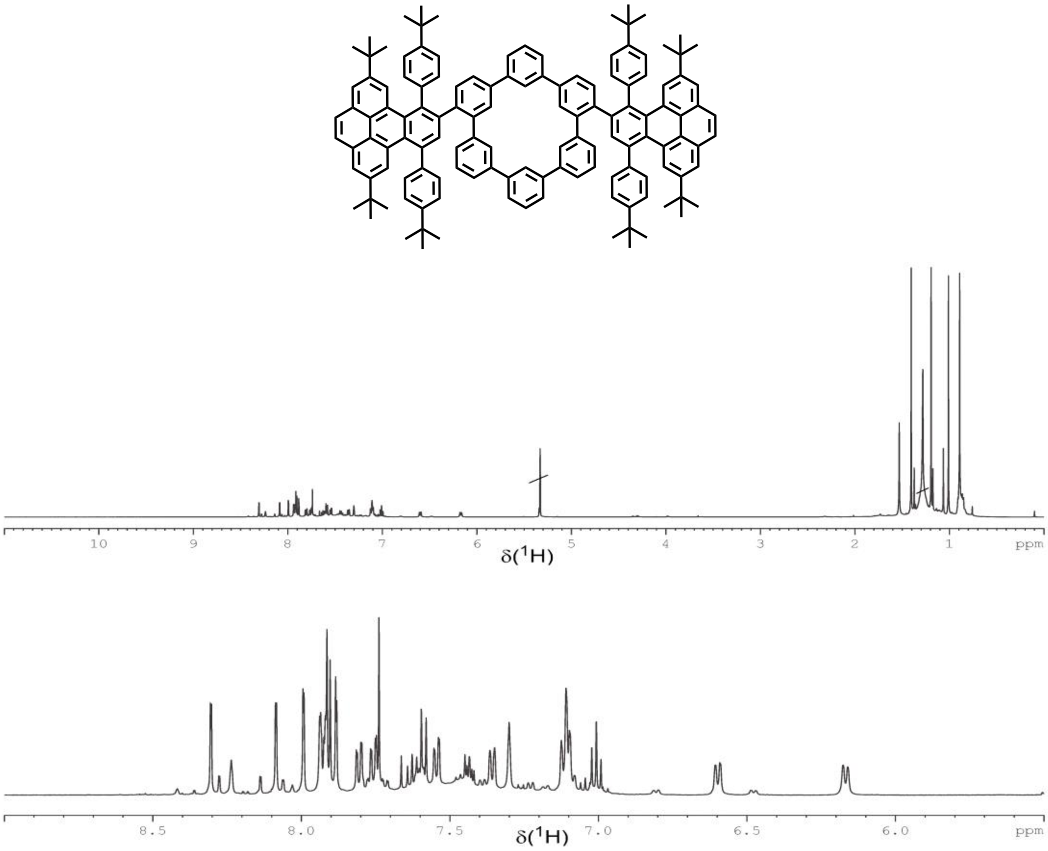

Figure S46. ^{1}H NMR spectrum (500 MHz) of **12** in CD_2Cl_2 at 30°C.

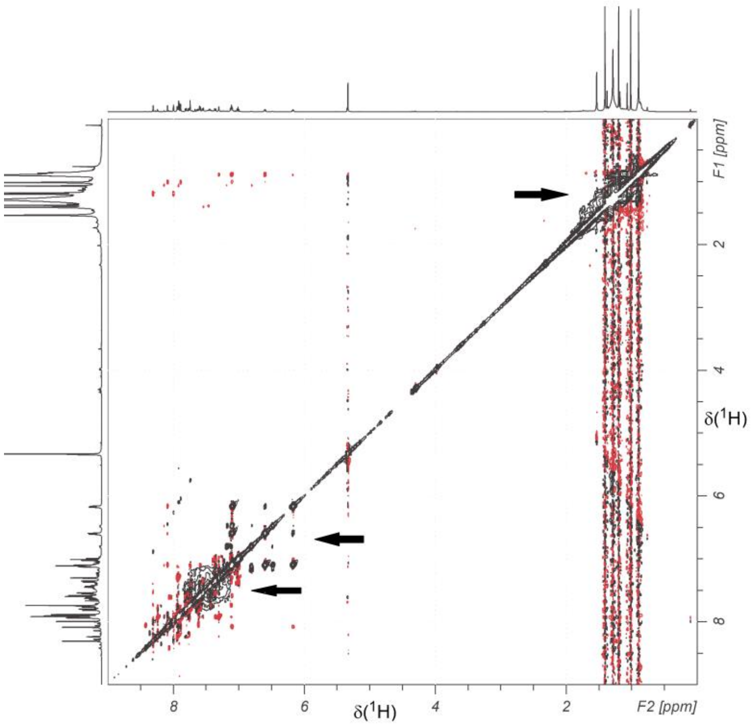

Figure S47. EXSY (black correlations, regions marked with arrows)/ROESY (red correlations) spectrum of **12** (CD_2Cl_2).

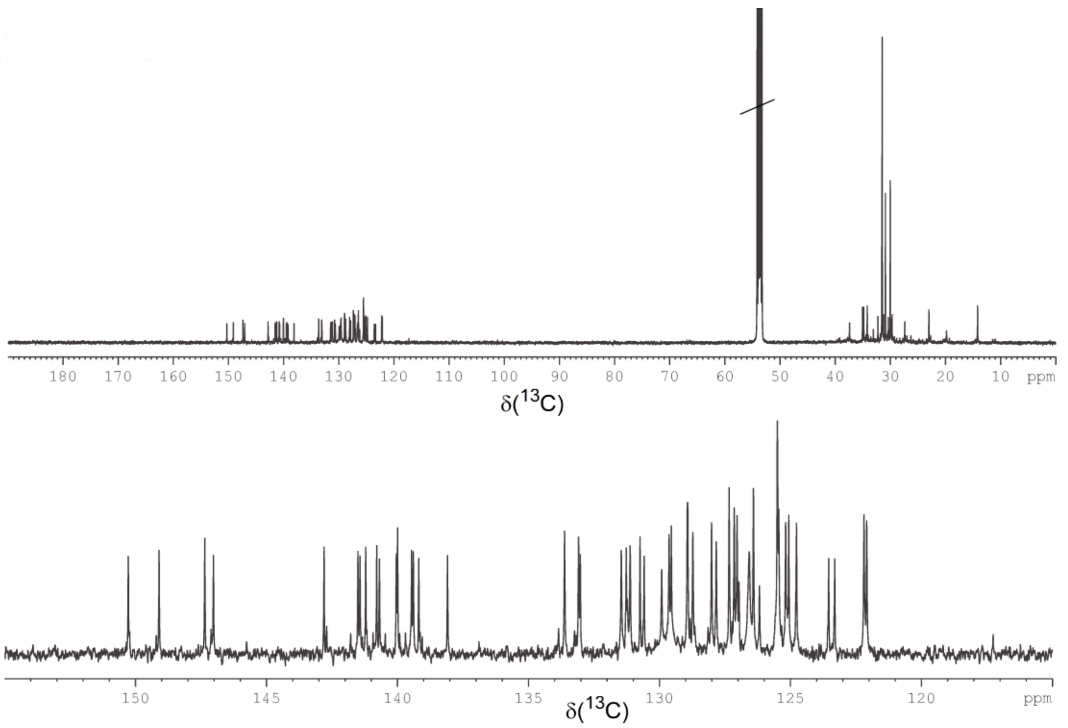

Figure S48. ^{13}C NMR spectrum (125 MHz) of **12** in CD_2Cl_2 at 30°C.

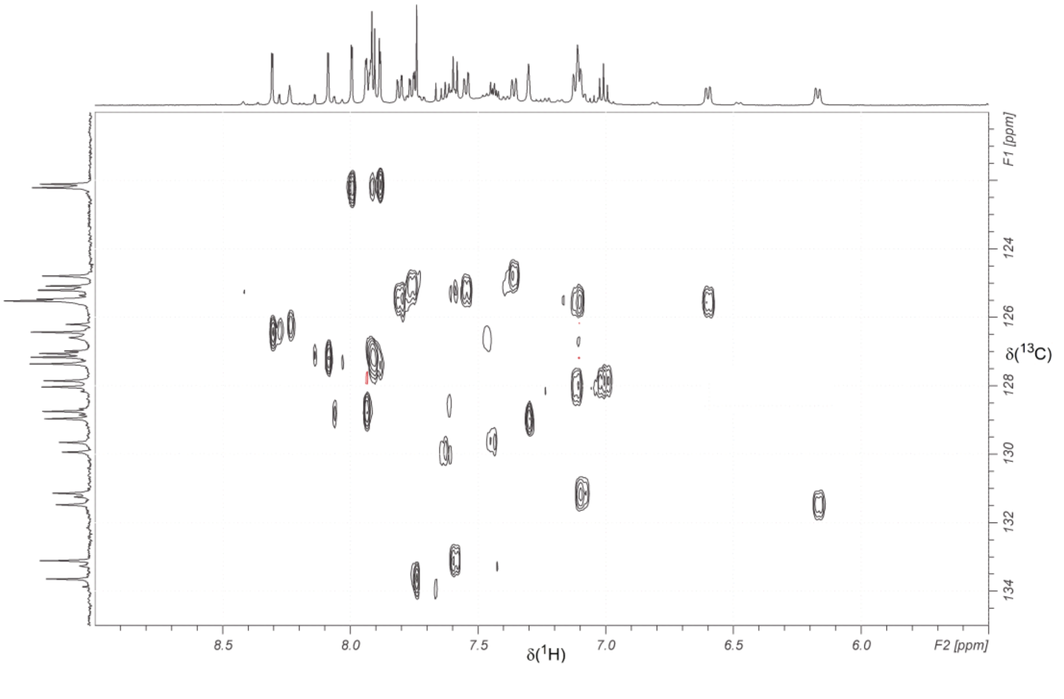

Figure S49. HSQC spectrum (region of aromatic CH) of **12** in CD_2Cl_2 at 30°C. The F1 trace is the DEPT135 spectrum.

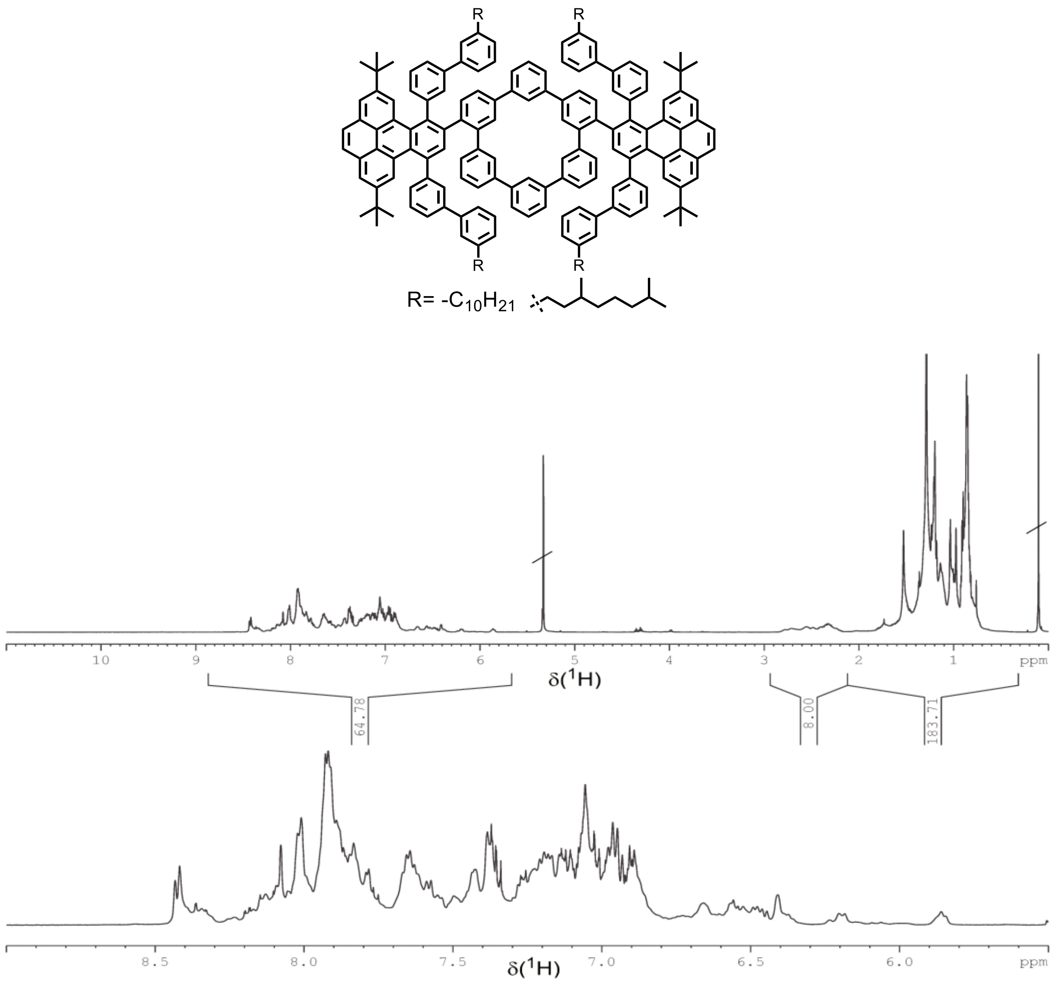

Figure S50. ^{1}H NMR spectrum (500 MHz) of **14** in CD_2Cl_2 at 30°C.

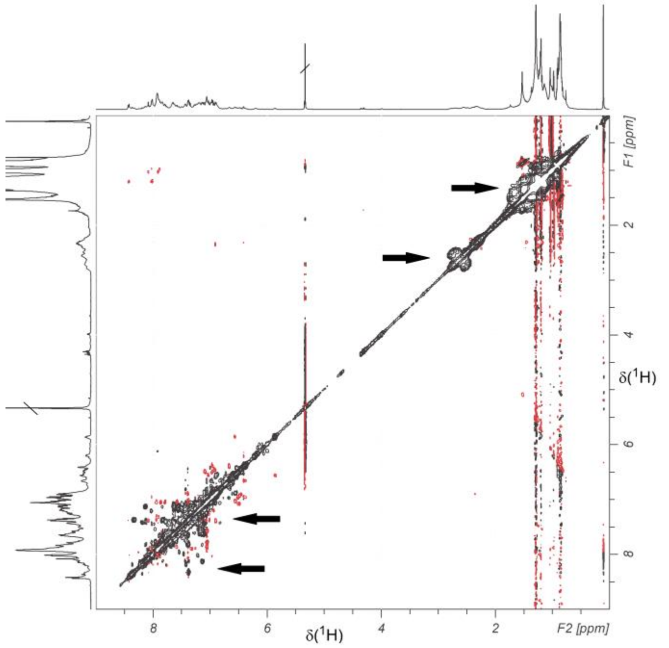

Figure S51. EXSY (black correlations, regions marked with arrows)/ROESY (red correlations) spectrum of **14** (CD_2Cl_2).

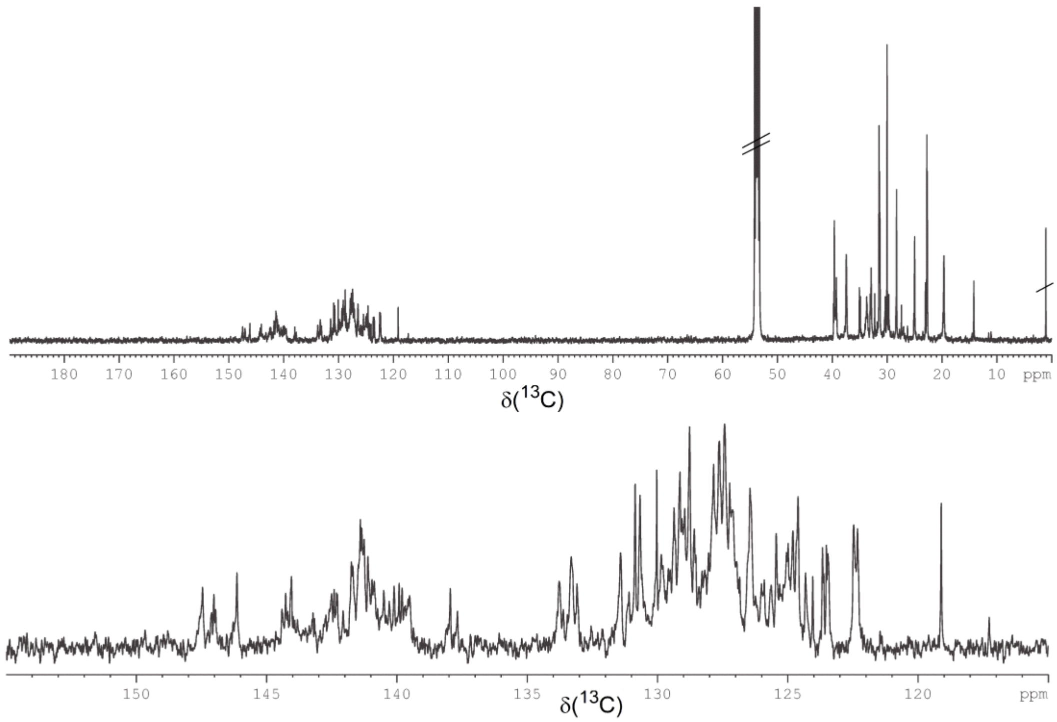

Figure S52. ^{13}C NMR spectrum (125 MHz) of **14** in CD_2Cl_2 at 30°C.

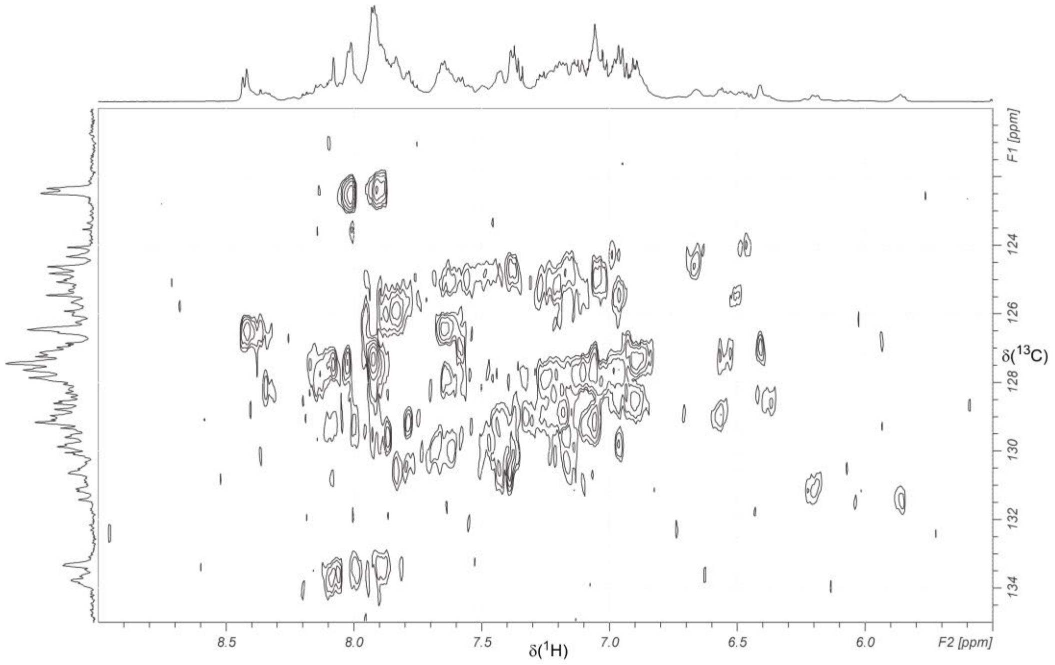

Figure S53. HSQC spectrum (region of aromatic CH) of **14** in CD_2Cl_2 at 30°C. The F1 trace is the DEPT135 spectrum.

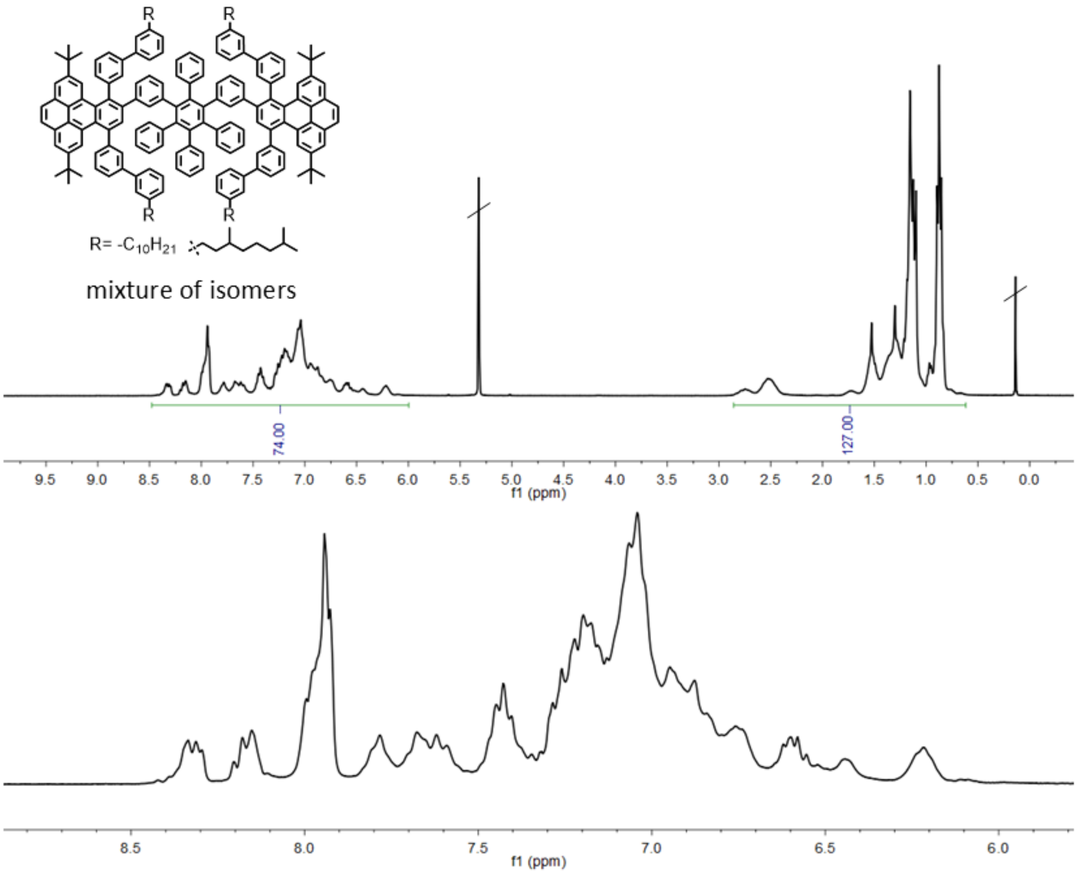

Figure S54. ^{1}H NMR spectrum (300 MHz) of **15** in CD_2Cl_2 at room temperature.

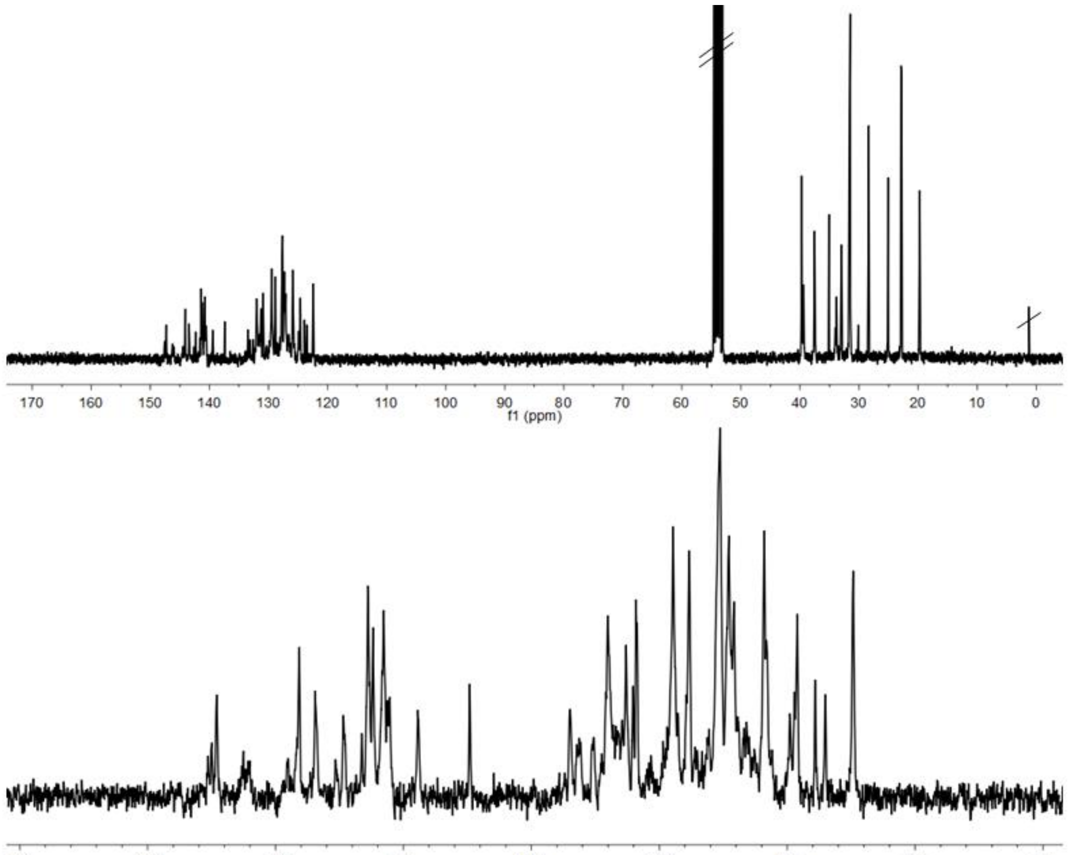

Figure S55. ^{13}C NMR spectrum (75 MHz) of **15** in CD_2Cl_2 at room temperature.

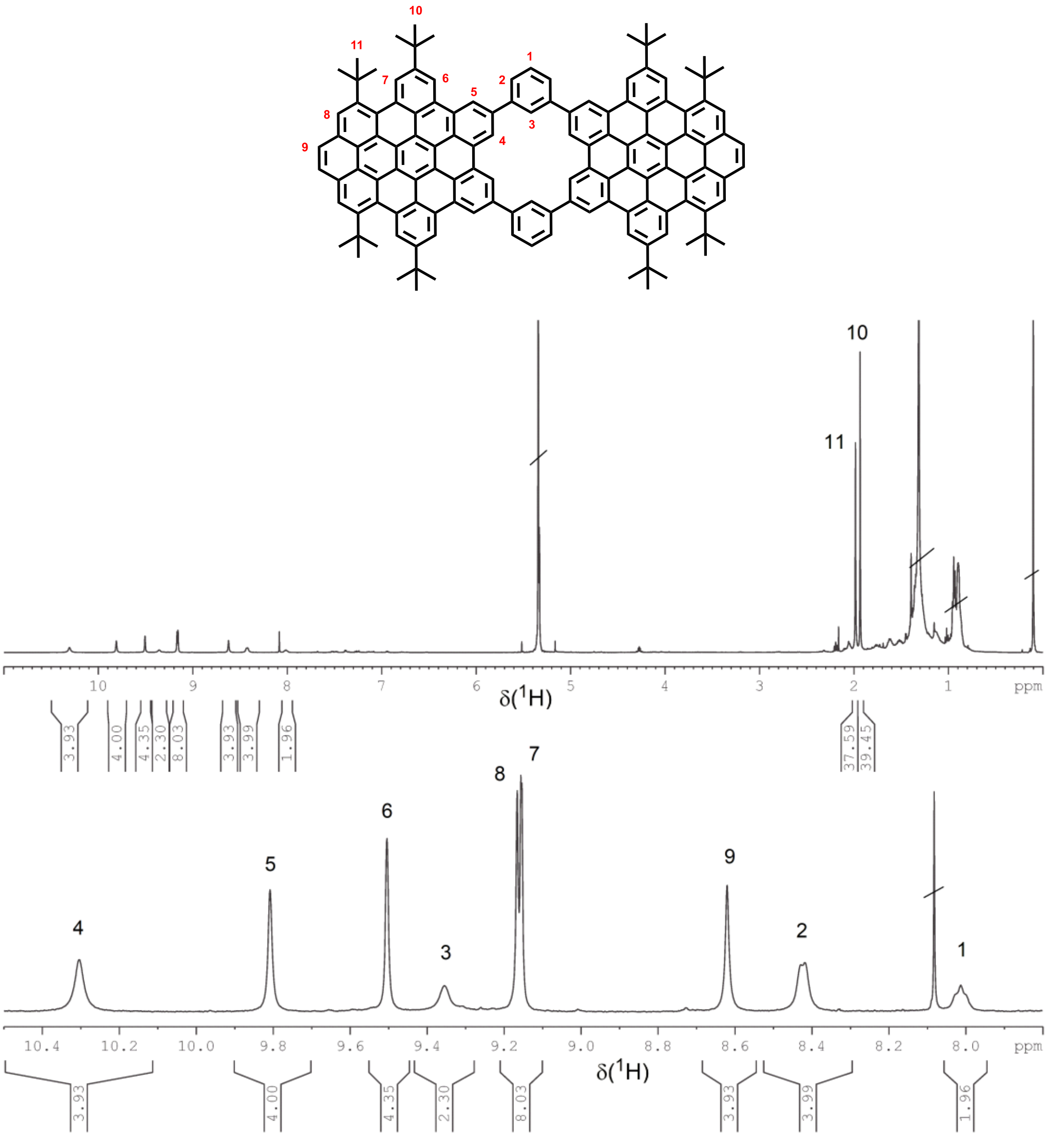

Figure S56. ^{1}H NMR spectrum (500 MHz) of **1** in CD_2Cl_2/CS_2 1/1 v/v at 30°C.

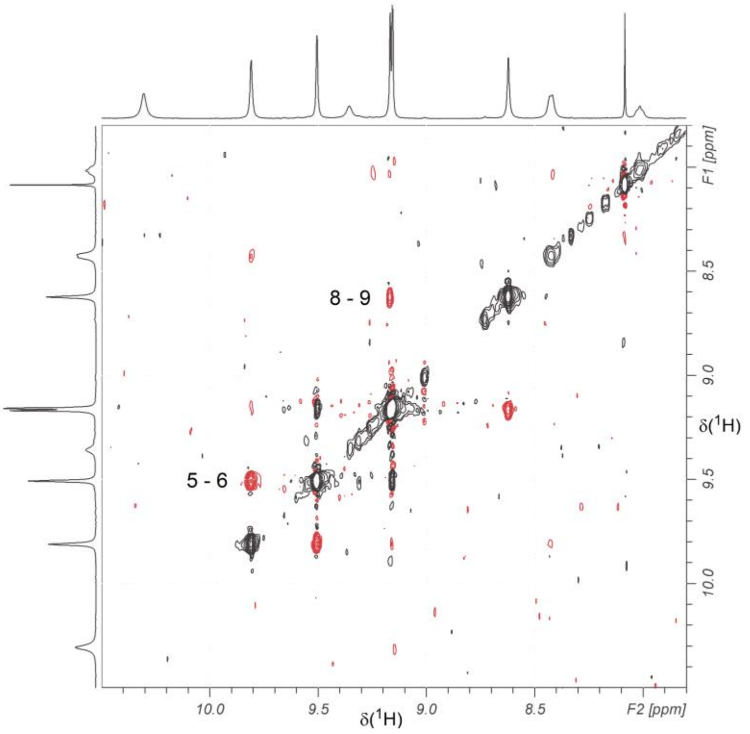

Figure S57. ROESY spectrum (region of aromatic CH) of **1** in in CD_2Cl_2/CS_2 1/1 v/v at 30°C.

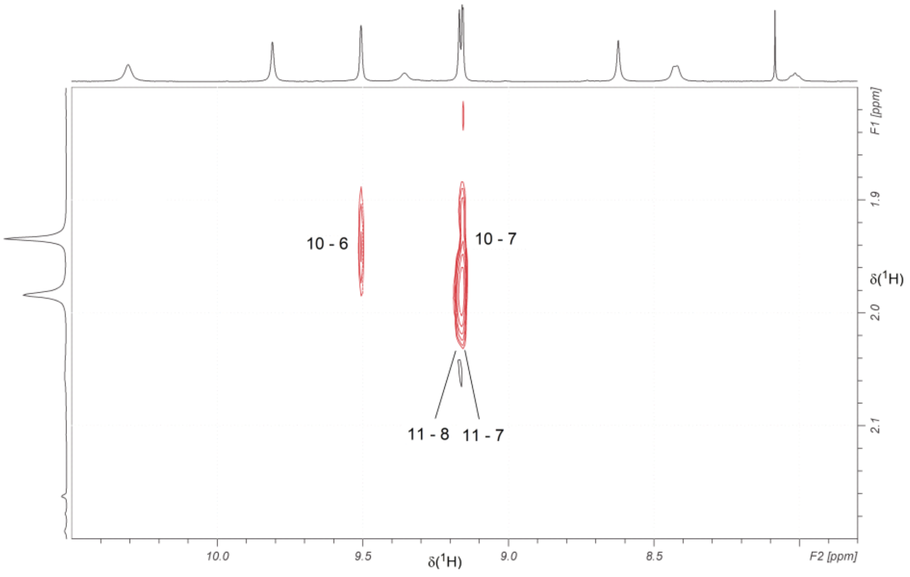

Figure S58. ROESY spectrum (region shows the correlations between tBu protons and aromatic CH) of **1** in in CD_2Cl_2/CS_2 1/1 v/v at 30°C.

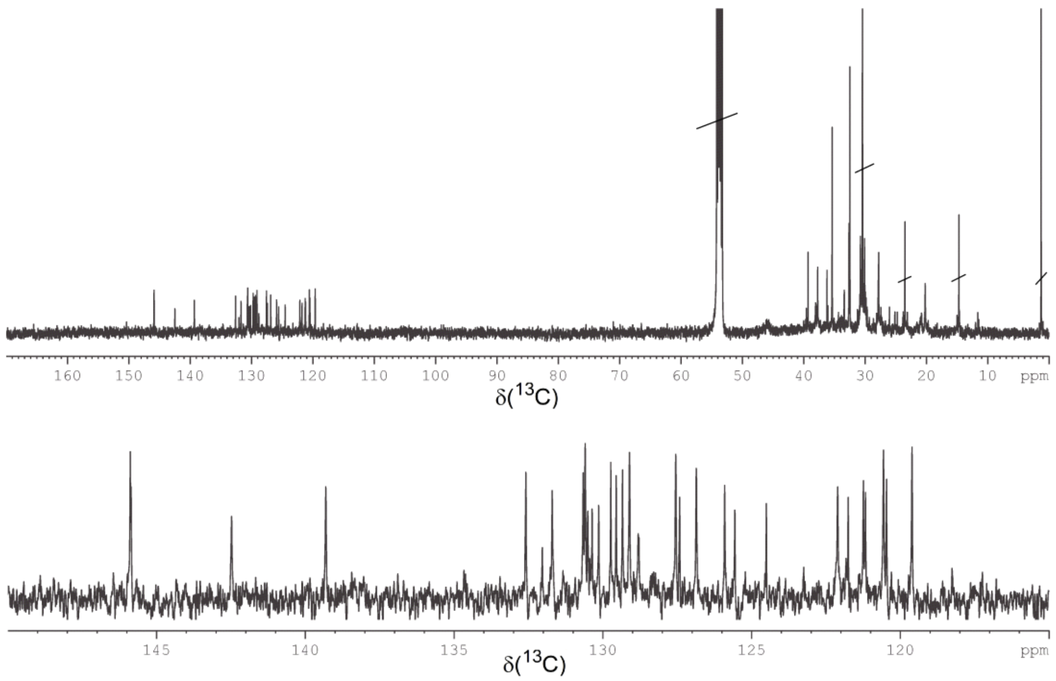

Figure S59. ^{13}C NMR spectrum (125 MHz) of **1** in in CD_2Cl_2/CS_2 1/1 v/v at 30°C.

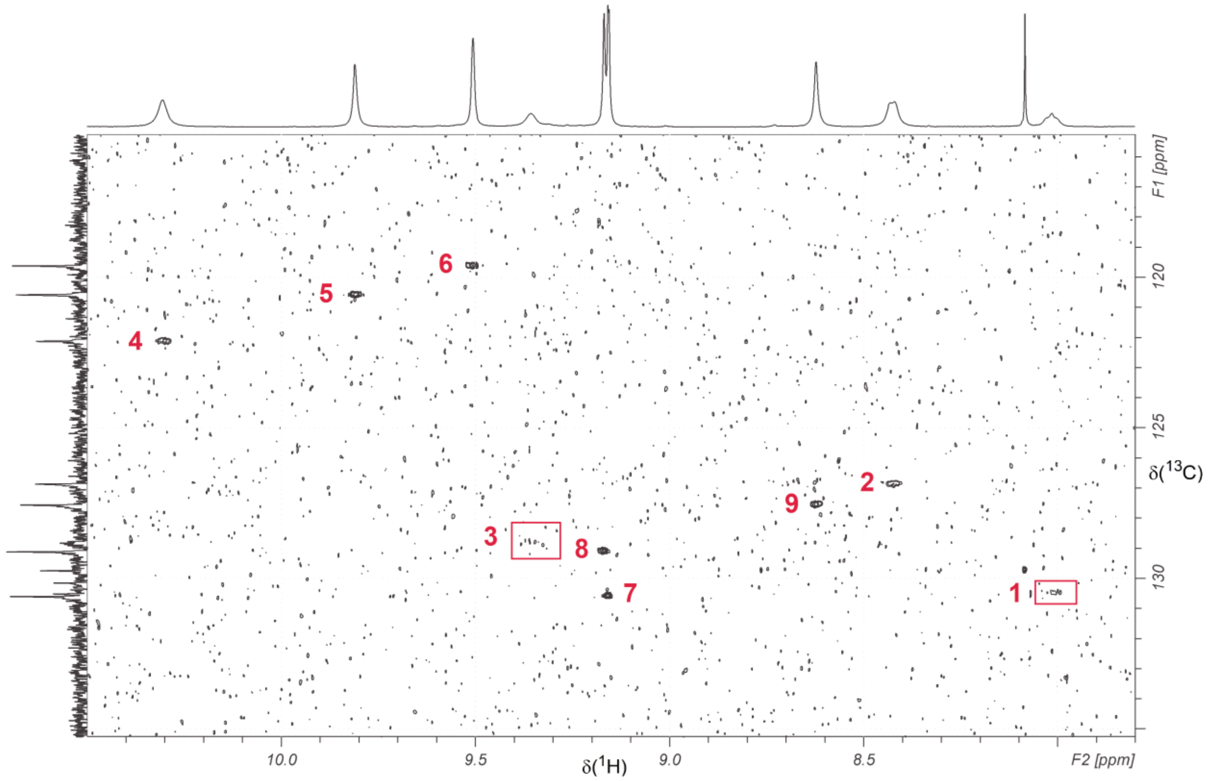

Figure S60. HSQC spectrum (region of aromatic CH) of **1** in in CD_2Cl_2/CS_2 1/1 v/v at 30°C. The F1 trace is the DEPT135 spectrum.

11. References

(1) Qiu, Z.-L.; Chen, D.; Deng, Z.; Chu, K.-S.; Tan, Y.-Z.; Zhu, J. Isolation of a Carbon Nanohoop with Möbius Topology. *Sci. China Chem.* **2021**, *64*, 1004–1008.

(2) Baumgärtner, K.; Meza Chincha, A. L.; Dreuw, A.; Rominger, F.; Mastalerz, M. A Conformationally Stable Contorted Hexabenzoovalene. *Angew. Chem. Int. Ed.* **2016**, *55*, 15594–15598.

(3) Chen, Y.-Y.; Wang, H.; Zhang, D.-W.; Hou, J.-L.; Li, Z.-T. Organogels Formed by Substituent-Free Pyrene-Appended Oligo(m-Phenylene Ethynylene)s. *Chem. Commun.* **2015**, *51*, 12088–12091.

(4) Hou, I. C.; Hinaut, A.; Scherb, S.; Meyer, E.; Narita, A.; Müllen, K. Synthesis of Giant Dendritic Polyphenylenes with 366 and 546 Carbon Atoms and Their High-vacuum Electrospray Deposition. *Chem. – Asian J.* **2022**, *17*, e202200220.

(5) Sauriat-Dorizon, H.; Maris, T.; Wuest, J. D.; Enright, G. D. Molecular Tectonics. Construction of Porous Hydrogen-Bonded Networks from Bisketals of Pentaerythritol. *J. Org. Chem.* **2003**, *68*, 240–246.

(6) Han, H.; Hu, S.; Zhang, S.; Li, X.; Sun, H.; Chen, J.; Liu, B.; Liu, C.; Chen, W.; Zhang, Q. Achieving Solution-Processed Non-Doped Single-EmittingLayer White Organic Light-Emitting Diodes through Adjusting Pyrene-Based Polyaromatic Hydrocarbon. *Chem. Eur. J.* **2022**, *28*, e202201741.

(7) Frisch, M. J.; Trucks, G. W.; Schlegel, H. B.; Scuseria, G. E.; Robb, M. A.; Cheeseman, J. R.; Scalmani, G.; Barone, V.; Petersson, G. A.; Nakatsuji, H.; Li, X.; Caricato, M.; Marenich, A. V.; Bloino, J.; Janesko, B. G.; Gomperts, R.; Mennucci, B.; Hratchian, H. P.; Ortiz, J. V.; Izmaylov, A. F.; Sonnenberg, J. L.; Williams; Ding, F.; Lipparini, F.; Egidi, F.; Goings, J.; Peng, B.; Petrone, A.; Henderson, T.; Ranasinghe, D.; Zakrzewski, V. G.; Gao, J.; Rega, N.; Zheng, G.; Liang, W.; Hada, M.; Ehara, M.; Toyota, K.; Fukuda, R.; Hasegawa, J.; Ishida, M.; Nakajima, T.; Honda, Y.; Kitao, O.; Nakai, H.; Vreven, T.; Throssell, K.; Montgomery, Jr., J. A.; Peralta, J. E.; Ogliaro, F.; Bearpark, M. J.; Heyd, J. J.; Brothers, E. N.; Kudin, K. N.; Staroverov, V. N.; Keith, T. A.; Kobayashi, R.; Normand, J.; Raghavachari, K.; Rendell, A. P.; Burant, J. C.; Iyengar, S. S.; Tomasi, J.; Cossi, M.; Millam, J. M.; Klene, M.; Adamo, C.; Cammi, R.; Ochterski, J. W.; Martin, R. L.; Morokuma, K.; Farkas, O.; Foresman, J. B.; Fox, D. J. *Gaussian 16, Revision C.01*; Gaussian Inc., Wallingford CT, 2016.

(8) Dovesi, R.; Erba, A.; Orlando, R.; Zicovich-Wilson, C. M.; Civalleri, B.; Maschio, L.; Rérat, M.; Casassa, S.; Baima, J.; Salustro, S.; Kirtman, B. Quantum-Mechanical Condensed Matter Simulations with CRYSTAL. *WIREs Comput. Mol. Sci.* **2018**, *8* (4), e1360.

(9) Tommasini, M.; Lucotti, A.; Alfè, M.; Ciajolo, A.; Zerbi, G. Fingerprints of Polycyclic Aromatic Hydrocarbons (PAHs) in Infrared Absorption Spectroscopy. *Spectrochim. Acta. A. Mol. Biomol. Spectrosc.* **2016**, *152*, 134–148.

(10) Fulmer, G. R.; Miller, A. J. M.; Sherden, N. H.; Gottlieb, H. E.; Nudelman, A.; Stoltz, B. M.; Bercaw, J. E.; Goldberg, K. I. NMR Chemical Shifts of Trace Impurities: Common Laboratory Solvents, Organics, and Gases in Deuterated Solvents Relevant to the Organometallic Chemist. *Organometallics* **2010**, *29* (9), 2176–2179.

(11) Kresse, G.; Furthmüller, J. Efficient Iterative Schemes for *Ab Initio* Total-Energy Calculations Using a Plane-Wave Basis Set. *Phys. Rev. B* **1996**, *54* (16), 11169–11186.

(12) Blöchl, P. E. Projector Augmented-Wave Method. *Phys. Rev. B* **1994**, *50* (24), 17953–17979.

(13) Perdew, J. P.; Burke, K.; Ernzerhof, M. Generalized Gradient Approximation Made Simple. *Phys. Rev. Lett.* **1996**, *77* (18), 3865–3868.

(14) Grimme, S.; Antony, J.; Ehrlich, S.; Krieg, H. A Consistent and Accurate *Ab Initio* Parametrization of Density Functional Dispersion Correction (DFT-D) for the 94 Elements H-Pu. *J. Chem. Phys.* **2010**, *132* (15), 154104.

(15) Heyd, J.; Scuseria, G. E.; Ernzerhof, M. Hybrid Functionals Based on a Screened Coulomb Potential. *J. Chem. Phys.* **2003**, *118* (18), 8207–8215.

(16) Tries, A.; Osella, S.; Zhang, P.; Xu, F.; Ramanan, C.; Kläui, M.; Mai, Y.; Beljonne, D.; Wang, H. I. Experimental Observation of Strong Exciton Effects in Graphene Nanoribbons. *Nano Lett.* **2020**, *20*, 2993–3002.

(17) Yang, L.; Cohen, M. L.; Louie, S. G. Excitonic Effects in the Optical Spectra of Graphene Nanoribbons. *Nano Lett.* **2007**, *7* (10), 3112–3115.
(18) Prezzi, D.; Varsano, D.; Ruini, A.; Marini, A.; Molinari, E. Optical Properties of Graphene Nanoribbons: The Role of Many-Body Effects. *Phys. Rev. B* **2008**, *77* (4), 041404.
(19) Denk, R.; Hohage, M.; Zeppenfeld, P.; Cai, J.; Pignedoli, C. A.; Söde, H.; Fasel, R.; Feng, X.; Müllen, K.; Wang, S.; Prezzi, D.; Ferretti, A.; Ruini, A.; Molinari, E.; Ruffieux, P. Exciton-Dominated Optical Response of Ultra-Narrow Graphene Nanoribbons. *Nat. Commun.* **2014**, *5* (1), 4253.
